# Green Accelerated Synthesis, Antimicrobial Activity and Seed Germination Test of Quaternary Ammonium Salts of 1,2-bis(4-pyridyl)ethane

**DOI:** 10.3390/molecules24132424

**Published:** 2019-07-01

**Authors:** Aurel Tăbăcaru, Andreea Veronica Dediu Botezatu, Georgiana Horincar, Bianca Furdui, Rodica Mihaela Dinică

**Affiliations:** 1 Faculty of Sciences and Environment, Department of Chemistry, Physics and Environment, “Dunarea de Jos” University of Galati, 111 Domneasca Street, 800201 Galati, Romania; 2 Faculty of Food Science and Engineering, Department of Food Science, Food Engineering and Applied Biotechnology, “Dunarea de Jos” University of Galati, 111 Domneasca Street, 800201 Galati, Romania

**Keywords:** quaternary ammonium salts, 1,2-bis(4-pyridyl)ethane, green synthesis, antimicrobial activity, seed germination

## Abstract

A family of fifteen quaternary ammonium salts (QAs), bearing the 1,2-bis(4-pyridyl)ethane core, were obtained using for the first time two different green methods, such as microwave (MW) and ultrasounds (US) irradiation, with very good yields and in much shorter times compared to the classical method, and an assay on their antimicrobial action against *Escherichia coli* (*E. coli*) was carried out. While 12 to 24 h were required for complete alkylation of 1,2-bis(4-pyridyl)ethane by reactive halogenated derivatives in anhydrous solvent under reflux conditions, MW and US irradiation reduced the reaction time and the desired products were achieved in a few min. One of the aims of this study was to evaluate the antibacterial potential of the synthesized QAs against pathogenic bacteria, along with their impact on germination activity of wheat seeds (*Triticum aestivum* L.). The antibacterial activity of the QAs against *Escherichia coli* was explored by determining the minimum inhibitory concentration (MIC). The MIC values varied from 0.312 to 2.5 mg/mL, highlighting the lowest values attained for the derivatives containing methoxy, chlorine and benzofurane functional groups. The viability of aerobic bacteria was determined with the Tetrazolium/Formazan Test, a method that was found to be the best alternative approach with respect to the difuzimetric method. Seeds of *Triticum aestivum* L. were used for the evaluation of the germination indicators, such as seed germination (SG), the relative seed germination (RSG), the relative radicle growth (RRG), and the seed germination index (GI). The toxicity studies of QAs **1**, **4** and **7**, at two different concentrations, showed no inhibitory effect on seed germination.

## 1. Introduction

“Green chemistry” is amongst the fast-growing approaches in the different fields of science and technology that involves the reduction or the elimination of use and production of toxic materials through proper invention, design, and application [1]. Recently, microwave assisted reactions has arisen as one of the most convenient and reproducible methods for synthesis of various heterocyclic compounds due to its green and sustainable nature. In view of this, a number of authors have reported the microwave-assisted synthesis of various organic compounds, which have been widely applied in the drug discovery field, due to enhanced reaction rates, high yields, improved purity, and greener reaction condition [2,3,4,5,6].

Numerous reports have shown that microwave heating have the capacity to accelerate chemical reactions, to increase reaction yield and to enhance product’s purity and material’s properties as compared to conventional experiments in which heating by convection or conduction is used. Microwave heating has been used, e.g., in chemical functionalization of chitosan or cellulose, to obtain such as modified polysaccharides, at lower reaction time as compared to conventional methods and without any degradation of the biopolymer chain [7]. The microwave assisted organic synthesis, with green advantages like eco-friendly, good yields and reducing reaction times, is the need of today for organic synthetic chemists. The change of the counter anions to appreciate their synergetic effects in the microwave-assisted of ionic liquids is also a great interest subject [8].

The use of ultrasound irradiation to promote organic synthesis has also attracted the interest of the scientific community [2,9,10]. Various chemical and physical effects occur due to the formation of the acoustic cavitation from ultrasound waves in a liquid medium or solid mixture and this brings many advantages such as time-saving, easy work-up procedure, enhanced reaction rates, and eco-friendly reaction conditions [11].

The quaternary ammonium salts (QAs) are known as one of the most visible and effective classes of disinfectants for nearly a century. However, it has been demonstrated that pathogenic microorganisms were resistant to the action of some disinfectants and antibiotics [12]. Thus, it was followed by identifying a new source of antimicrobial compounds. Generally, the mechanism of action of QAs is the disruption of the cell membrane, the one of the most fundamental structures in bacteria, which leads to cell lysis and bacterial death [13]. The antimicrobial effect of QAs against bacteria, fungi, and viruses was investigated [12,14,15,16,17]. Also, the inhibitory potential of some of these compounds (e.g., quaternary ammonium salts of bis(pyridinium)) has been reported by Furdui and coworkers [17]. QAs are regarded as surfactants that are widely used for the control of bacterial growth in medical, food, and industrial environments [18,19]. For this reason, phytotoxicity studies should be carried out on plants.

The pathogenic microorganisms have been a long threat to human health and social development, because they can develop infections and diseases in animals, plants, and humans. One of the leading causes of death worldwide is the outbreak infectious disease induced by bacteria, fungi, and viruses, which triggers the death of over a quarter of the world population annually [20,21]. To reduce the number of infections with pathogens and to combat them, different classes of antimicrobial agents have developed very fast. However, the widespread use of these products has induced the emergence of new strains of antimicrobial resistant microorganisms, which caused increased difficulties in combating infections [22,23].

The present paper is focused on the improved synthesis of fifteen quaternary ammonium compounds of 1,2-bis(4-pyridyl)ethane by green methods (microwave and ultrasounds irradiation), along with the first results regarding the evaluation of antibacterial potential against *Escherichia coli*. The effects of quaternary ammonium salts (QAs) of bis(pyridinium) on seed germination of wheat (*Triticum aestivum* L.) is also presented and discussed.

## 2. Results and Discussion

### 2.1. Synthesis

**Scheme 1:**
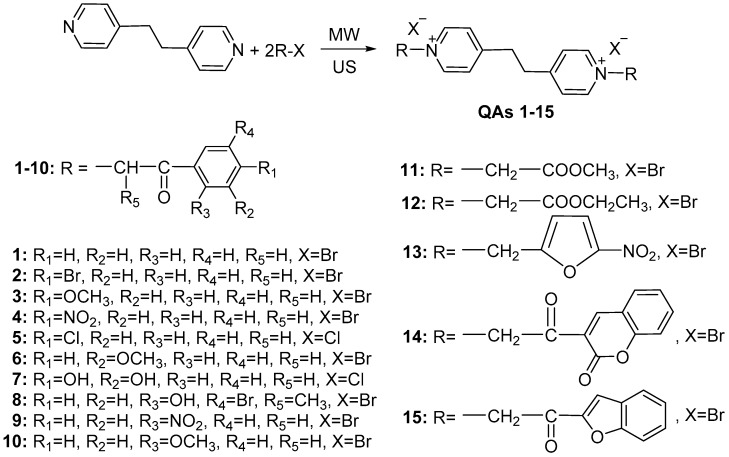
Synthesis of quaternary ammonium salts of 1,2-bis(4-pyridyl)ethane (QAs **1**–**15**).

All fifteen quaternary ammonium salts (QAs) were obtained by an improved methodology with respect to the already reported classical solution-synthesis by Furdui et al. [17,24], that is, the green alternatives using microwave (MW) and ultrasounds (US) irradiation. Generally, the most organic reactions have been heated by traditional heating equipment which are rather slow, and local overheating can lead to decomposition of product reagent or substrate. In contrast, accelerations by MW and US have been observed for a wide range of organic reactions offering a lot of advantages connected to safety, enhancement in reactivity and selectivity, cost saving, and energy and pollution prevention [25,26,27,28]. According to this new strategy, a mixture of 1,2-bis(4-pyridyl)ethane and halide derivative in the 1:2 molar ratio, in the presence of 0.5 equivalents of anhydrous acetonitrile was subjected to both MW and US irradiation (Scheme 1). The extremely fast procedure resulted in successfully obtaining all fifteen QAs with very good yields that were very close to those of the classical method, where much higher solvent volumes are used (150 equivalents of anhydrous acetonitrile).

In Table 1, a comparison between the reaction conditions and yields among the classical solution-synthesis and the two green methods is given, thus making obvious the remarkable advantage of these green alternatives in terms of time saving and yields. In the case of reactions with the yield of the synthesis lower than 90%, unreacted reagents identified by thin layer chromatography were observed, but due to their solubility in the reaction medium they were removed by washing the solid powder with acetonitrile. Reactions were monitored after 5, 10, and 15 min. The increase in reaction time for the MW-assisted reaction was found to no longer increase the yield of the reaction. In Table 1 the time interval of 5–10 min, which is the best approach for the synthesis of such QAs, was thus reported. It is important to highlight the fact that MW irradiation was particularly efficient, that is, only a few min were necessary to obtain the desired products in excellent yields, whereas hours of conventional heating under reflux conditions could give the same results. The US irradiation was also effective, the yields being excellent in all cases and the reaction times were reduced from 12–24 h under conventional heating to just one hour, these results also being similar to those reported in the literature for other classes of compounds [4]. Reactions were performed on two ultrasonic baths, one of 30 Hz and another one of 35 Hz. The use of the US bath of 35 Hz, presented in the manuscript, was more efficient with a maximum reaction time of 60 min, while the other one requires an hour and 30 min. Taking into account the need to obtain faster synthesis methods in medicine, it is essential to report the most effective methods of synthesis. To the best of our knowledge, this is the first time when MW- and US-promoted reactions for the synthesis of QAs are employed, thus opening the way towards the generation of many other types of QAs derivatives.

### 2.2. Evaluation of Antibacterial Activity of QAs Against E. coli

The antibacterial activity of QAs is a hot research topic and there are several papers that study the synthesis, characterization, and antimicrobial properties of the QAs and their derivatives [29,30,31]. The class of tetrazolium salts contain a large group of organic compounds which have the ability to form highly colored products, well known as formazans [32,33]. This trait can be used to detect and to measure the cell viability [34,35,36,37]. Nowadays, the tetrazolium salts are widely used in different chemical and biological applications [32,37,38].

In the present study, the evaluation of antibacterial activity of QAs was performed by using the TTC test as a qualitative assay and the broth microdilution method as a quantitative method. We investigated the effects of selected QAs, with different electron-withdrawing/releasing effects, which may give different antimicrobial properties to the QAs. The minimum inhibitory concentrations (MICs) were determined as the lowest concentrations of QAs that inhibit the growth of bacteria.

#### 2.2.1. Evaluation of Inhibition Rate of QAs by the TTC Colorimetric Test

**Figure 1:**
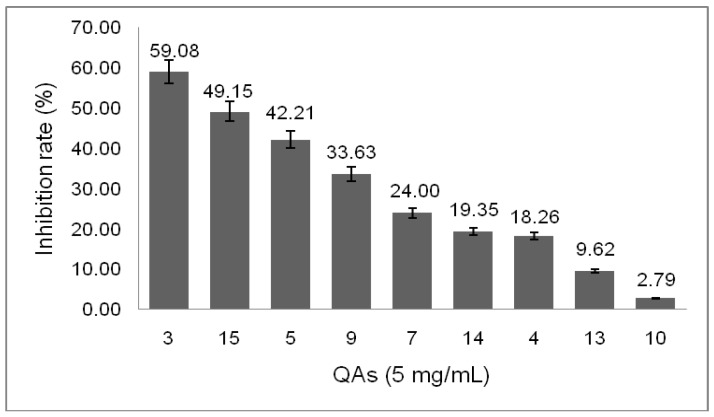
Inhibition rate (%) of *Escherichia coli* (*E. coli*) by QAs determined by Tetrazolium/Formazan Test.

In the present study, the TTC test proved to be an efficient colorimetric method for antibacterial evaluation. The results revealed the antibacterial potential of QAs expressed as inhibition rate of cell bacteria. The presence of vital cells of bacteria was evidenced by an intense red color from the transformed formazan in growth media. After the addition of QAs, the reduction of color intensity to colorless could be observed. After testing all the synthesized QAs against *E. coli*, only nine compounds have shown antibacterial activity. From Figure 1 it can be observed that the inhibition rate of bacteria varied from 3% to 59%, depending on the composition of salts. Thus, a strong inhibitory effect (59%) against *E. coli* was shown by QAs **3**, followed by QAs **15** (50%), QAs **5** (42%) and QAs **9** (33%). Also, it was observed that the vital cells of bacteria were inhibited by QAs **4**, **7** and **14**, with an average inhibition rate not higher than 20%. A reduced inhibitory potential against the tested bacteria was reached by QAs **13**, with an inhibition rate of 10%, while QAs **10** had the lowest inhibitory effect (3%).

Few investigations have also been carried out on the antibacterial properties of QAs using TTC test. In agreement with these data, the TTC application as indicator for the antibacterial activity proved to be an efficient evaluation method of bacteria viability [38,39]. In few papers, the antimicrobial activity of QAs and their derivatives have also been suggested [24,38,40]. The antibacterial action of QAs consisted disruption of lipid membrane through association of the positively charged quaternary nitrogen with the polar head groups of acidic phospholipids [31,41].

The physical and chemical properties, and also the biologic activities of the QAs are dictated by various factors and counterions that are used. The understanding of the properties of the salts with different structures and counterions is important in the selection of a candidate for potentially active QAs [42]. We also demonstrated previously that there was a good relationship between structural descriptors (LogP, polarizability, polar surface area (2D), and Van der Waals surface area (3D)) and antibacterial properties of these kinds of QAs [16].

#### 2.2.2. Minimum Inhibitory Concentrations (MIC)

The MICs of QAs were determined as a quantitative evaluation of their antibacterial activity which exerts different ranges of sensitivity against Gram negative bacteria. The MIC values varied from 0.312 to 2.5 mg/mL (Table 2). The lowest MIC value (0.312 mg/mL) was shown by QAs **3**, **5** and **15** against *E. coli*. Some differences in antibacterial activities were observed for some tested compounds. Thus, QAs **7**, **9** and **14** showed higher MIC values, that is 0.625 mg/mL, while QAs **10** and **13** showed the highest MIC value, that is 2.5 mg/mL.

The results are in agreement with those determined by Tetrazolium/Formazan Test (Figure 1). Also, the results showed a higher antibacterial potential of our QAs compared to, e.g., dimethylaminoethyl methacrylate quaternized by hexadecyl bromide against *E. coli* [43]. Moreover, the MIC values of other QAs, such as benzylidenehydrazinyl pyridinium salts were investigated by Alptüzün et al., who showed that *E. coli* was inhibited with variable concentrations from 2 to 128 µg/mL [38]. Some papers have also reported an antimicrobial performance of polymeric film with QAs-containing coatings against *E. coli*, e.g., about 99% of biocidal efficiency even after 96 h [44].

### 2.3. Germination Activity

The impact of different types of compounds on terrestrial plants has been studied extensively [45,46]. When growing, plants absorb relatively large amounts of essential and non-essential elements, which can be toxic to certain concentrations, so seed germination is a very sensitive test to measure the toxicity and the influence of different external factors. The Organisation for Economic Cooperation and Development (OECD) is an international organization that works to build better policies for better lives. OECD Guidelines for the Testing of Chemicals are periodically reviewed in the light of scientific progress and current regulatory procedures. The selection of species should be based on the ecological relevance of species, species specific life-cycle characteristics, region of natural occurrence, etc. The response of an organism to a chemical depends on the dose. Nonlinear dose-response relationships occur across a broad range of research fields and are a well-established tool to describe the basic mechanisms of phytotoxicity [47]. Wheat (*Triticum aestivum* L.) has been extensively studied for its allelopathic potential. The allelopathic activity of wheat has been attributed to hydroxamic acids and related compounds, and phenolic acids [48]. The literature data, focused on toxicity studies on wheat germination seeds, determined us to choose them as model of study due to their low cost and handiness, as well as one of the most important food for humans.

Considering the wide spectrum of potential applications of QAs as surfactants that are widely used for the control of bacterial growth in medical, food, and industrial environments, we investigated the effects of three selected QAs, with electronic withdrawing effects (-I and/or -E), on wheat seed germination, as the first step towards estimating the potential hazard of these chemical substances to the enviroment. QAs **1**, **4,** and **7** were selected also to see if various functional groups can lead to toxic effects, considering that, for instance, the nitro group is shown to induce toxicity. Taking into account the literature data, throughout this test, we evaluated the most sensitive physiological parameters in plants.

**Figure 2:**
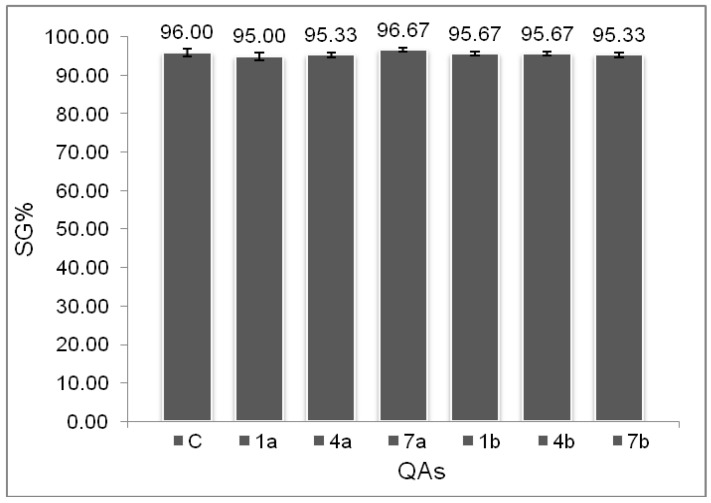
Seed germination (SG%) of *Triticum aestivum* L. exposed to different concentrations of quaternary ammonium salts (QAs) **1**, **4**, and **7**. Error bars represent ± standard deviation of 3 replicates.

**Figure 3:**
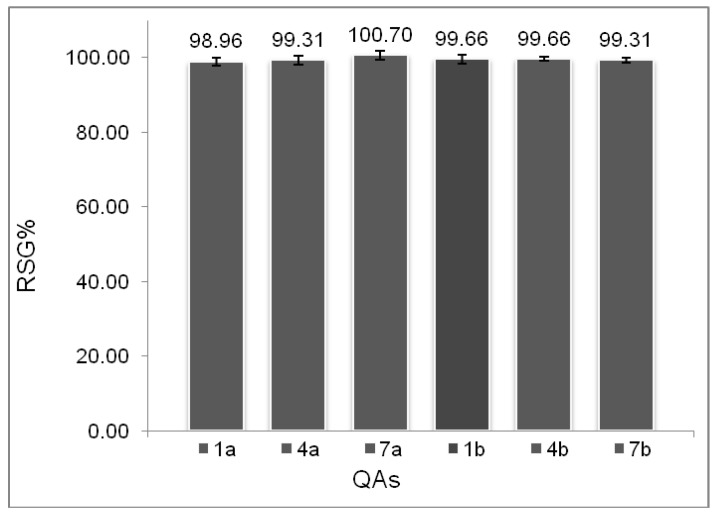
Relative seed germination (RSG%) of *Triticum aestivum* L. exposed to different concentrations of QAs **1**, **4**, and **7**. Error bars represent ± standard deviation of 3 replicates.

**Figure 4:**
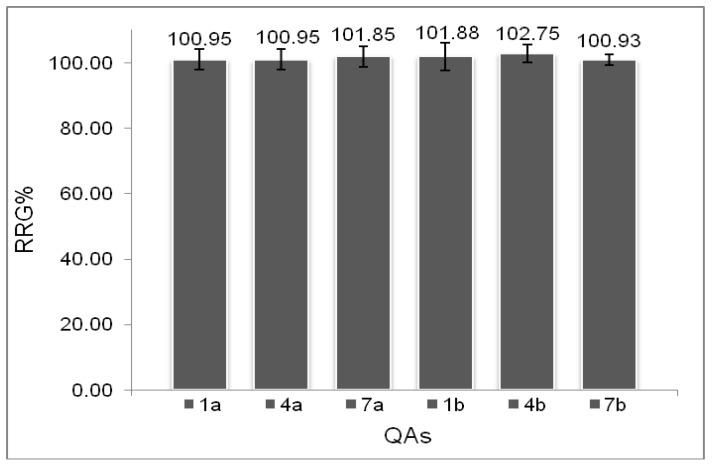
Relative radicle growth (RRG%) of *Triticum aestivum* L. exposed to different concentrations of QAs **1**, **4**, and **7**. Error bars represent ± standard deviation of 3 replicates.

**Figure 5:**
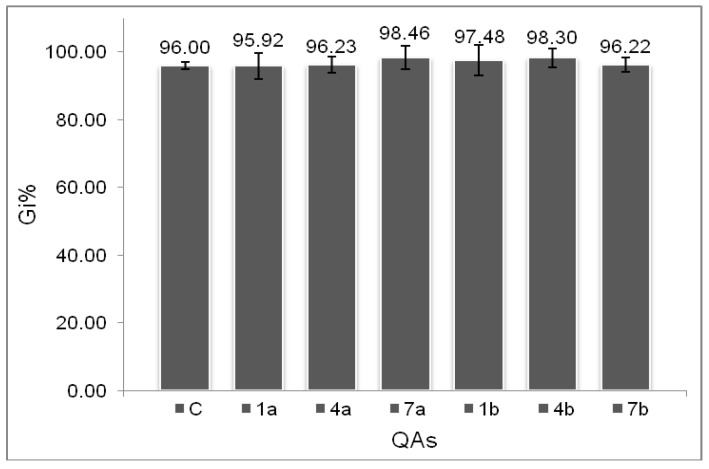
Germination index (Gi%) of *Triticum aestivum* L. exposed to different concentrations of QAs **1**, **4**, and **7**. Error bars represent ± standard deviation of 3 replicates.

The SG values, not less than 95% and not less than those of the control sample (Figure 2), and the relative seed germination percentages (RSG%), ranging from 98.96 to 100.70% for treated seeds (Figure 3), showed that the tested QAs had no influence on seed germination. Moreover, the relative radicle growth values (RRG) were not negatively influenced by the presence of tested compounds presenting values over 100% (Figure 4) [49]. The Gi values, greater than 95% and not very different from those of the control sample (Figure 5), means that the tested QAs have no phytotoxicity [50].

The toxicity studies of QAs **1**, **4,** and **7** at two different concentrations (a: 10^−5^ M and b: 10^−6^ M), exhibited no inhibitory effect on seed germination.

## 3. Materials and Methods 

### 3.1. General

All the reagents and organic solvents used for synthesis were purchased from Fluka (Grenoble, France) and Merck (Darmstadt, Germany). Seeds of wheat, recommended as model crops, were used for seed germination toxicity test and were supplied by a local seed supplier in Galati city, Romania.

Melting points were recorded with a Stuart SMP10 instrument (Keison Products, Essex, UK). FTIR spectra were recorded from 4000 to 650 cm^−1^ with a Perkin-Elmer Spectrum 100 instrument (Perkin-Elmer, Shelton, CT, USA) by ATR (Attenuated Total Reflectance) technique on a CdSe crystal. ^1^H-NMR and ^13^C-NMR spectra were recorded with a Bruker 400 Ultrashield spectrometer (Bruker Daltonics, Hamburg, Germany) (at 400 MHz for ^1^H-NMR and at 75 MHz for ^13^C-NMR) operating at room temperature (298 K), using DMSO-*d_6_* as the solvent and TMS as the internal standard. For the NMR data analysis, MestReNova software (Thermo Scientific, Waltham, USA) was used. Elemental analyses (C, H, N) were performed with a Fisons Instruments 1108 CHNS-O elemental analyzer (Thermo Scientific, Waltham, MA, USA).

### 3.2. Synthesis of QAs Under Microwave and Ultrasounds Irradiation

A typical procedure involves irradiation of a mixture of 1 equiv of 1,2-bis(4-pyridyl)ethane and 2 equivalents of halide derivative, using acetonitrile as assisting solvent (0.5 equivalents), under microwave (MW) irradiation for 10 min in a multimode microwave reactor (300 W, domestic MW oven) and ultrasounds (US) for 1h in an ultrasonic bath (Bandelin Sonorex Digitec) (Bandelin, Berlin, Germany) (operating frequency 35 Hz, with a digital timer (30 s to 30 min) and a heater, allowing solution heating to be set from 20 to 60 °C). The reactions irradiated by MW were carried out in sealed vessels under controlled conditions (temperature, time). The temperature in the ultrasonic bath was controlled by adding ice periodically. The increase in reaction time for the MW-assisted reaction was found to no longer increase the yield of the reaction, ten min being the best approach. In the end of the reaction, after cooling to room temperature, the isolated solid powders were washed with anhydrous acetonitrile and dried under vacuum at 60 °C and then characterized by NMR (Figures S16–S30 from the Supplementary Materials). In the end of the reaction, after cooling to room temperature, the salts recovery was done by simply adding them into acetonitrile to allow their filtration. Characterization by NMR spectroscopy and elemental analysis indicated their purity. The structure of the products was confirmed by spectral data and comparison with authentic samples prepared according to the literature methods [29,32].

*N,N’-Diphenacyl-1,2-bis-(4-pyridinium)ethane dibromide(QAs **1**)*: white-beige crystals; m.p. 290–292 °C (dec.). IR (ATR, cm^−1^): 3009; 2948; 1691; 1637; 1594, 1570, 1518; 1228; 995. ^1^H NMR (DMSO-d_6_) δ/ppm: 8.96 (d, *J* = 6.8 Hz, 4H), 8.27 (d, *J* = 6.8 Hz, 4H), 8.09 (d, *J* = 8.8 Hz, 4H), 7.82 (t, *J* = 7.43 Hz, 2H), 7.69 (t, *J* = 7.8 Hz, 4H), 6.48 (s, 4H), 3.50 (s, 4H). ^13^C NMR (DMSO-*d_6_*) δ ppm: 190.78 (2 C=O); 161.03 (2C); 145.72 (4C); 134.77 (2C); 133.49 (2C); 129.17 (4C); 128.23 (4C); 127.49 (4C); 65.64 (2CH_2_); 33.74 (2CH_2_). Anal. Calcd. for C_28_H_26_Br_2_N_2_O_2_ (M_r_ = 582.33 g/mol): C, 57.75; H, 4.50; N, 4.81. Found: C, 57.53; H, 4.68; N, 4.91.

*N,N’-Di(p-bromophenacyl)-1,2-bis-(4-pyridinium)ethane dibromide(QAs **2**)*: white crystals; m.p. > 350 °C (dec.). IR (ATR, cm^−1^): 3011; 2933, 2885; 1696; 1642; 1585, 1586; 1235; 1180; 990. ^1^H NMR (DMSO-d_6_) δ/ppm: 8.912 (d, *J* = 6.4 Hz, 4H), 8.24 (d, *J* = 6.4 Hz, 4H), 8.01 (d, *J* = 8 Hz, 4H), 7.93 (d, *J* = 8 Hz, 4H), 6.41 (s, 4H: 2CH_2_), 3.48 (s, 4H: 2CH_2_). ^13^C NMR (DMSO-d_6_) δ/ppm: 193.52 (2 C=O); 152.87 (2C); 148.44 (4C); 134.07 (2C); 131.76 (4C); 131.5 (4C); 128.973 (2C); 125.28 (4C); 65.79 (2CH_2_); 33.53 (2CH_2_). Anal. Calcd. for C_28_H_24_Br_4_N_2_O_2_ (M_r_ = 740.12 g/mol): C, 45.44; H, 3.27; N, 3.78. Found: C, 45.22; H, 3.22; N, 3.73.

*N,N’-Di(p-methoxyphenacyl)-1,2-bis-(4-pyridinium)ethane dibromide(QAs **3**)*: white crystals; m.p. 300–302 °C (dec.). IR (ATR, cm^−1^): 3004; 2958, 2835; 1674; 1636; 1599, 1571, 1556; 1230, 1028; 1170; 998. ^1^H NMR (DMSO-d_6_) δ/ppm: 8.95 (d, *J* = 6.8 Hz, 4H), 8.26 (d, *J* = 6.8 Hz, 4H), 8.05 (d, *J* = 8.93 Hz, 4H), 7.21 (d, *J* = 8.93 Hz, 4H), 6.37 (s, 4H: 2 CH_2_), 3.88 (s, 6H: 2OCH_3_), 3.54 (s, 4H: 2 CH_2_). ^13^C NMR (DMSO-d_6_) δ/ppm: 190.64 (2 C=O); 161.05 (2C); 159.58 (2C), 145.70 (4C); 134.81 (2C); 130.45 (4C); 127.5 (4C); 123.66 (4C); 65.72 (2CH_2_); 55.59 (CH_3_: OCH_3_); 33.73 (2CH_2_). Anal. Calcd. for C_30_H_30_Br_2_N_2_O_4_ (M_r_ = 642.38 g/mol): C, 56.09; H, 4.71; N, 4.36. Found. C, 55.73; H, 4.67; N, 4.20.

*N,N’-Di(p-nitrophenacyl)-1,2-bis-(4-pyridinium)ethane dibromide(QAs **4**)*: beige crystals; m.p. 307–309 °C (dec.). IR (ATR, cm^−1^): 3030; 2886, 2822; 1703; 1642, 1601; 1523, 1346; 1222, 1199–1181; 994. ^1^H NMR (DMSO-d_6_, TMS) δ/ ppm: 8.96 (d, *J* = 6.8 Hz, 4H), 8.495 (d, *J* = 6.8 Hz, 4H), 8.32–8.28 (m, 8H), 6.53 (s, 4H: 2CH_2_), 3.51 (s, 4H: 2CH_2_). ^13^C NMR (DMSO-d_6_) δ/ppm: 190.21 (2 C=O); 161.26 (2C); 150.63 (2C), 145.71 (4C); 138.20 (2C); 129.73 (4C); 127.57 (4C); 124.19 (4C), 65.89 (2CH_2_); 33.77 (2CH_2_). Anal. Calcd. for C_28_H_24_Br_2_N_4_O_6_ (M_r_ = 672.32 g/mol): C, 50.02; H, 3.60; N, 8.33. Found. C, 49.86; H, 3.67; N. 8.25.

*N,N’-Di(p-chlorophenacyl)-1,2-bis-(4-pyridinium)ethane dichloride(QAs **5**)*: white crystals, m.p. 302–304 °C (dec.). IR (ATR, cm^−1^): 3011–3048; 2986–2887; 1694; 1643; 1589–1586, 1521; 1240, 1177, 1093; 991. ^1^H NMR (DMSO-d_6_) δ/ppm: 8.93 (d, *J* = 6 Hz, 4H), 8.24 (d, *J* = 6 Hz, 4H), 8.08 (d, *J* = 8Hz, 4H), 7.77 (d, *J* = 8Hz, 4H), 6.45 (s, 4H: 2 CH_2_), 3.47 (s, 4H: 2 CH_2_). ^13^C NMR (DMSO-d_6_) δ/ppm: 190.58 (2 C=O); 161.13 (2C); 157.20 (2C), 147.78 (4C); 141.20 (2C), 132.17 (4C); 131.37 (4C); 129.53 (4C); 65.74 (2CH_2_); 33.78 (2CH_2_). Anal. Calcd. for C_28_H_24_Cl_4_N_2_O_2_ (M_r_ = 562.31 g/mol): C, 59.81; H, 4.30; N, 4.98. Found: C, 59.45; H, 4.32; N, 5.09.

*N,N’-Di(m-methoxyphenacyl)-1,2-bis-(4-pyridinium)ethane dibromide(QAs **6**)*: white crystals; m.p. 302–303 °C (dec.). IR (ATR, cm^−1^): 3008; 2951; 1690; 1639; 1597–1581; 1263, 1194–1220; 998. ^1^H NMR (DMSO-d_6_) δ/ppm: 8.95 (d, *J* = 6.8 Hz, 4H), 8.26 (d, *J* = 6.8 Hz, 4H), 7.69–7.55 (m, 6H), 7.41–7.38 (m, 2H), 6.46 (s, 4H: 2CH_2_), 3.88 (s, 6H: 2OCH_3_), 3.54 (s, 4H: 2CH_2_). ^13^C NMR (DMSO-d_6_) δ /ppm: 190.64 (2 C=O); 161.05 (2C); 159.58 (2C), 145.70 (4C); 134.81 (2C); 130.45 (2C); 127.5 (4C); 120.66 (2C); 120.49 (2C), 112.94 (2C); 65.72 (2CH_2_); 55.59 (CH_3_: OCH_3_) 33.73 (2CH_2_). Anal. Calcd. for C_30_H_30_Br_2_N_2_O_4_ (M_r_ = 642.38 g/mol): C, 56.09; H, 4.71; N, 4.36. Found: C, 55.93; H, 4.88; N. 4.31.

*N,N’-Di(3,4-dihydroxyphenacyl)-1,2-bis-(4-pyridinium)ethane dichloride(QAs **7**)*: white crystals; m.p. 330–332 °C (dec.). IR (ATR, cm^−1^): 3059–2737; 1681; 1638; 1606–1593, 1518; 1342; 1291, 1167; 1131.39. ^1^H NMR (DMSO-d_6_) δ/ppm: 10.38 (s, 2H: 2OH), 9.67 (s, 2H: 2OH), 8.91(d, *J* = 6 Hz; 4H), 8.20 (d, *J* = 6 Hz; 4H), 7.47–7.43 (m, 4H), 6.98 (d, *J* = 8 Hz, 2H), 6.31 (s, 4H: 2CH_2_), 3.45 (s, 4H: 2CH_2_). ^13^C NMR (DMSO-d_6_) δ/ppm: 188.64 (2 C=O); 160.77 (2C); 152.30 (2C), 149.40 (2C), 145.68 (2C); 145.53 (2Ct); 127.33 (4C); 123.98 (4C); 115.51 (2C), 114.95 (2C), 65.12 (2CH_2_); 33.37 (2CH_2_). Anal. Calcd. for C_28_H_26_Cl_2_N_2_O_6_ (M_r_ = 557.42 g/mol): C, 60.33; H, 4.70; N, 5.03. Found: C, 59.99; H, 4.74; N, 4.93.

*N,N’-Di(5-bromo-2-hydroxy-propiophenacyl)-1,2-bis-(4-pyridinium)ethane dibromide (QAs **8**)*: white-rose crystals; m.p. 262–263 °C (dec.). IR (ATR, cm^−1^): 3116–2903; 1667; 1635; 1591, 1474–1466 1407; 1285; 1169; 988. ^1^H NMR (DMSO-d_6_) δ/ppm: 11.57 (s, 2H: 2OH), 9,11 (d, *J* = 8Hz, 4H), 8.21 (d, *J* = 8Hz, 4H), 7.82 (s, 2H), 7.70 (m, 2H), 7.09 (d, *J* = 8 Hz, 2H), 6.66 (q, 2H: 2CH), 3.45 (s, 4H: 2CH_2_), 1.95 (d, 6H: 2CH_3_). ^13^C NMR (DMSO-d_6_) δ/ppm: 193.03 (2 C=O); 161.40 (2C); 157.22 (2C), 145.00 (4C); 138.12 (2C); 132.68 (2C); 126.97 (4C); 122.51 (2C), 120.08 (2C), 110.74 (2C), 73.18 (2CH); 33.817 (2CH_2_); 16.87 (2CH_3_). Anal. Calcd. for C_30_H_28_Br_4_N_2_O_4_ (M_r_ = 800.17 g/mol): C, 45.03; H, 3.53; N, 3.50. Found: C, 44.92; H, 3.65; N, 3.42.

*N,N’-Di(o-nitrophenacyl)-1,2-bis-(4-pyridinium)ethane dibromide(QAs **9**)*: beige crystals; m.p. > 250 °C (dec.). IR (cm^−1^): 3051, 2895, 2820, 2356, 1715, 1637, 1570, 1519, 1473, 1342, 1220, 1196, 996. ^1^H-NMR (DMSO-d_6_) δ/ppm: 8.99 (d, *J* = 6.8 Hz, 4H, H_ortho/N+_), 8.34 (d, *J* = 6.8 Hz, 4H, H_meta/N+_), 8.31–8.29 (m, 2H, Ph), 8.12–8.05 (m, 4H, Ph), 7.98–7.94 (m, 2H, Ph), 6.39 (s, 4H, CH_2_/N^+^), 3.52 (s, 4H, CH_2_-CH_2_). ^13^C-NMR (DMSO-*d*_6_) δ ppm: 192.83 (C=O), 161.61 (C), 145.96 (C), 145.45 (CH), 134.68 (CH), 133.25 (C), 131.24 (CH), 128.88 (CH), 127.84 (CH), 124.68 (CH), 66.70 (CH_2_), 33.81 (CH_2_). Anal. Calcd. for C_28_H_24_Br_2_N_4_O_6_ (M = 672.32 g/mol): C, 50.02; H, 3.60; N, 8.33. Found: C, 49.92; H, 3.71; N, 8.25.

*N,N’-Di(o-methoxyphenacyl)-1,2-bis-(4-pyridinium)ethane dibromide (QAs **10**)*: white crystals; m.p. 241–243 °C (dec.). IR (cm^−1^): 3001, 2945, 2876, 2358, 1667, 1639, 1594, 1520, 1484, 1466, 1435, 1337, 1285, 1241, 1207, 1188, 1162, 1112, 1010, 987. ^1^H-NMR (DMSO-*d*_6_) δ/ppm: 8.95 (d, *J* =6.8 Hz, 4H, H_ortho/N+_), 8.23 (d, *J* = 6.8 Hz, 4H, H_meta/N+_), 7.90 (dd, *J* = 7.8, 1.6 Hz, 2H, Ph), 7.79–7.74 (m, 2H, Ph), 7.38 (d, *J* = 8.0Hz, 2H, Ph), 7.19–7.15 (m, 2H, Ph), 6.22 (s, 4H, 2CH_2_/N^+^), 4.06 (s, 6H, OCH_3_), 3.48 (s, 4H, CH_2_-CH_2_). ^13^C-NMR (DMSO-*d*_6_) δ ppm: 190.19 (C=O), 160.76 (C), 160.08 (CH), 145.68 (CH), 136.46 (C), 130.40 (CH), 127.24 (CH), 122.86 (C), 120.97 (CH), 113.15 (CH), 69.29 (CH_2_), 56.33 (OCH_3_), 33.74 (CH_2_). Anal. Calcd. for C_30_H_30_Br_2_N_2_O_4_ (M = 642.38 g/mol): C, 56.09; H, 4.71; N, 4.36. Found: C, 55.99; H, 4.86; N, 4.21.

*N,N’-Di(carbomethoxy-methyl)-1,2-bis-(4-pyridinium)ethane dibromide (QAs **11**)*: white crystals; m.p. 181–182 °C (dec.). IR (ATR, cm^−1^): 3007; 2951; 1737; 1641; 1570, 1521; 1230–1201; 994. ^1^H NMR (DMSO-d_6_) δ/ppm: 9.00 (d, *J* = 6.8Hz, 4H), 8.23 (d, *J* = 6.8Hz, 4H’), 5.68 (s, 4H: 2CH_2_), 3.79 (s, 6H: 2 OCH_3_), 3.45 (s, 4H: 2 CH_2_). ^13^C NMR (DMSO-d_6_) δ/ppm: 166.97 (2 C=Oester); 161.50 (2C); 145.67 (4C); 127.48 (4C); 59.63 (2CH_2_); 53.14 (2CH_3_: OCH_3_); 33.66 (2CH_2_). Anal. Calcd. for C_18_H_22_Br_2_N_2_O_4_ (M_r_ = 490.19 g/mol): C, 44.10; H, 4.52; N, 5.71. Found: C, 43.93; H, 4.60; N, 5.66.

*N,N’-Di(carboethoxy-methyl)-1,2-bis-(4-pyridinium)ethane dibromide (QAs **12**)*: white-rose crystals; m.p. 191–192 °C (dec.). IR (ATR, cm^−1^): 3006; 2941; 1725; 1639; 1571, 1521; 1234–1197; 1010. ^1^H NMR (DMSO-d_6_) δ/ppm: 9.01 (d, *J* = 6.8Hz, 4H), 8.24 (d, *J* = 6.8Hz, 4H), 5.67 (s, 4H: 2CH_2_), 4.25 (q, *J* = 7.1 Hz, 4H: 2 CH_2_), 3.45 (s, 4H: 2 CH_2_), 1.27 (t, *J* = 7.1 Hz, 6H: 2 CH_3_). ^13^C NMR (DMSO-d_6_) δ/ppm: 166.47 (2 C=Oester); 161.45 (2C); 145.66 (4C); 127.47 (4C); 62.30 (2CH_2_); 59.66 (2CH_2_: OCH_2_); 33.67 (2CH_2_); 13.94 (2CH_3_). Anal. Calcd. for C_20_H_26_Br_2_N_2_O_4_ (FW=518.24 g/mol): C, 46.35; H, 5.06; N, 5.41. Found: C, 46.23; H, 5.19; N, 5.34.

*N,N’-Di(5’-nitrofuran-methylen)-1,2-bis-(4-pyridinium)ethane dibromide (QAs **13**):* beige crystals; m.p. 250–251 °C (dec.). IR (ATR, cm^−1^): 3033–3013; 2952–2920; 1636; 1536; 1501, 1351; 1234, 1161; 1015. ^1^H NMR (DMSO-d_6_) δ/ppm: 9.10 (d, *J* = 6.4 Hz; 4H), 8.17 (d, *J* = 6.4Hz; 4H), 7.75 (d, *J* = 3.6Hz, 2H), 7.18 (d, *J* = 3.6 Hz; 2H), 6.05 (s, 4H: 2 CH_2_), 3.39 (s, 4H: 2 CH_2_). ^13^C NMR (DMSO-d_6_) δ/ppm: 152.05 (2C); 150.45 (2C); 146.57 (4C); 130.24 (4C); 117.94 (2C), 115.64 (2C), 56.94 (2CH_2_); 35.80 (2CH_2_). Anal. Calcd. for C_22_H_20_Br_2_N_4_O_6_ (M_r_ = 596.23 g/mol): C 44.30; H 3.38; N 9.40. Found C. 44.59; H. 3.35; N. 9.10.

*N,N’-Di(carbocoumaryl-methyl)-1,2-bis-(4-pyridinium)ethane dibromide (QAs **14**)*: white-beige crystals; m.p. > 300 °C (dec.). IR (cm^−1^): 3049, 3022, 2994, 2945, 2875, 2359, 2337, 1698, 1643, 1601, 1556, 1444, 1368, 1344, 1184, 979. ^1^H-NMR (DMSO-*d*_6_) δ/ppm: 8.96 (s, 2H, coumarin), 8.89 (d, *J* = 6.4 Hz, 4H, H_ortho/N+_), 8.21 (d, *J* = 6.4 Hz, 4H, H_meta/N+_), 8.07 (d, *J* = 7.4 Hz, 2H, coumarin), 7.85 (t, *J* = 8.4 Hz, 2H, coumarin), 7.57 (d, *J* = 8.4Hz, 2H, coumarin), 7.49 (t, *J* = 7.4Hz, 2H, coumarin), 6.27(s, 4H,CH_2_/N^+^), 3.47 (s, 4H, CH_2_-CH_2_). ^13^C-NMR (DMSO-*d*_6_) δ/ppm: 188.09 (C=O), 158.73(C=O), 154.84 (C), 149.52 (CH), 145.63 (C), 145.50 (CH), 131.39 (C), 128.32 (CH), 127.28 (C), 126.24 (CH), 125.37 (CH), 121.19 (CH), 113.42 (CH), 61.04 (CH_2_), 33.78 (CH_2_). Anal. Calcd. for C_34_H_26_Br_2_N_2_O_6_ (M = 718.38g/mol): C, 56.84; H, 3.65; N, 3.90. Found: C, 56.99; H, 3.75; N, 3.75.

*N,N’-Di(carbobenzofuranyl-methyl)-1,2-bis-(4-pyridinium)ethane dibromide (QAs **15**)*: yellow-beige crystals; m.p. > 300 °C (dec.). IR (cm^−1^): 3010, 2970, 2893, 2631, 1687, 1643, 1612, 1549, 1518, 1474, 1347, 1291, 1155, 1136, 1019, 928. ^1^H-NMR (DMSO-*d*_6_) δ/ppm: 9.02 (d, *J* = 6.8 Hz, 4H, H_ortho/N+_), 8.30 (d, *J* = 6.8 Hz, 4H, H_meta/N+_), 8.20 (s, 2H, benzofuran), 7.97 (dd, *J* = 8.0, 1.0 Hz, 2H, benzofuran), 7.85 (dd, *J* = 8.4, 1.0 Hz, 2H, benzofuran), 7.64–7.69 (m, 2H, benzofuran), 7.49–7.46 (m, 2H, benzofuran), 6.43 (s, 4H, CH_2_/N^+^), 3.52 (s, 4H, CH_2_-CH_2_). ^13^C-NMR (DMSO-*d*_6_) δ/ppm: 181.31 (C=O), 161.34 (C), 155.17 (C), 149.36 (C), 145.81 (CH), 129.46 (CH), 127.51 (CH), 126.46 (CH), 125.02 (C), 124.12 (CH), 115.69 (CH), 112.37 (CH), 64.86 (CH_2_), 33.75 (CH_2_). Anal. Calcd. for C_32_H_26_Br_2_N_2_O_4_ (M = 662.37 g/mol): C, 58.03; H, 3.96; N, 4.23. Found: C, 58.18; H, 4.14; N, 4.08.

### 3.3. Bacteria Strain and Growth Conditions

The pathogenic bacteria, *Escherichia coli* ATCC 25922 (clinical isolate), used as test microorganism for antibacterial activity study, were collected from the Microbial Culture Collection (Sf. Andrei Hospital of Galati, Romania). The cultivation medium for *E. coli* was Brain Heart Infusion Broth (BHI, Merck, Darmstadt, Germany). Bacterial culture for antibacterial activity was prepared by picking colony in BHI agar plates and incubated for 24 h, followed by suspension in an appropriate medium (9 mL). The culture was grown aerobically for 20 h at 37 °C. For antibacterial assay, 1 mL of bacterial culture was diluted in 9 mL BHI medium to 10^6^ CFU/mL.

### 3.4. Antibacterial Activity Evaluation

#### 3.4.1. Tetrazolium/Formazan Test (TTC)

TTC (2,3,5-triphenyl tetrazolium chloride, Merck) was prepared by dissolving the powder in sterile water (5 mg/mL) at room temperature. In the presence of bacteria, TTC is reduced to red formazan. The red formazan obtained is directly proportional to the activity and viability of the bacterial cells [48]. The TTC test was evaluated as a qualitative antibacterial method. For susceptibility test using TTC, 1 mL of QAs (dissolved in sterile water, 5 mg/mL) and 100 µL of bacteria suspension (10^6^ CFU/mL) was added into 40 mL of BHI broth. The control sample contained only bacterial suspension and BHI broth. All flasks were incubated with shaking at 37 °C at 200× *g* for 4 h. Then, 1 mL from each flask containing the treated and control cultures was transferred to sterile Eppendorf tubes, and 100 µL of TTC were then introduced. All tubes were incubated at 37 °C for 20 min. The samples were centrifuged at 4000× *g* for 3 min, followed by decantation of the supernatants. The resulted formazan crystals were resuspended in ethanol (70%) and centrifuged again. The red formazan solution obtained at the end was measured by a microplate reader (Tecan Infinite 200PRO, Männedorf, Switzerland) at 480 nm. All experiments were done in triplicate and the relative cell viability (%) was represented by a percentage relative to the untreated control cells.

#### 3.4.2. Minimum Inhibitory Concentration (MIC) Determination

The MIC was defined as the lowest concentration of QAs that inhibit the growth of bacteria in a microdilution well plate [51]. For the broth microdilution test, 100 µL of BHI broth was added into each well of a sterile 96-well microtiter plate. 100 µL from QAs stock solutions (5 mg/mL) were added into the first well of the microplate and serial dilutions (5, 2.5, 1.25, 0.625, 0.312, 0.156, 0.078, 0.039, 0.019, and 0.009 mg of chemical compound/mL medium) were prepared. Then, 100 µL of the test bacteria cell suspension were dispensed into each well. For positive controls, wells with 100 µL of bacterial culture and 100 µL of BHI were used, while for negative controls, wells with 100 µL of QAs and 100 µL of BHI were used. The contents of the wells were mixed prior to incubation at 37 °C for 24 h. The MICs values were determined by absorbance intensity measured by a microplate reader (Tecan Infinite 200PRO, Switzerland) at 620 nm. All measurements of MIC values were done in triplicate.

### 3.5. General Germination Procedure

In this study, the seed germination and root elongation of wheat (*Triticum aestivum* L.) were used to evaluate the toxicity of three selected QAs.

#### 3.5.1. Pretreatment of Wheat Seeds

Prior to germination, 100 healthy and mature wheat seeds, distributed into 4 dishes, were selected and their surface was sterilized using sodium hypochlorite (10%) with a ratio of 1:5 (*w/v*) for 15 min, to prevent fungal growth, rinsed five times with demineralized sterile water and transferred to 10 × 100 mm Petri dishes with a double layer of filter paper. The grains were incubated for germination in the presence and in the absence of two different concentrations of QAs **1**, **4**, and **7** (a: 10^−5^ M and b: 10^−6^ M), in a chamber with humidity (80%) and temperature control (23 ± 2 °C), in absence of light. The used test volume was 200 μL. We analyzed at these concentrations to see the toxicity at an order of magnitude at which these compounds could be used, according to other similar studies [52]. The test duration was 4 days until the primary root reached 4 mm. Control seeds (C) were treated only with distilled water. In total, three replicates of 100 seeds for each condition were used.

#### 3.5.2. Measurements

Treated and untreated wheat seeds were used for the estimation of the germination indicators, including the seed germination (SG), the relative seed germination (RSG), the relative radicle growth (RRG) and the seed germination index (GI) [50,51], as follows:

SG = (Number of germinated seeds after 4 days/Number of total seeds) × 100%

RSG = (Number of germinated seeds(sample)/Number of germinated seeds (control)) × 100%

RRG = (Total radicle length of germinated seeds (sample)/Total radicle length of germinated seeds (control)) × 100%

GI = (%SG) × (%RRG)/100%

### 3.6. Statistical Analysis

All the experiments were done in triplicate and the data presented here represents the mean of these replicates. Data related to the MIC values of QAs against *E. coli* were subjected to analysis of variance (one way ANOVA) in Duncan multiple range test using SPSS (version 10) statistical software. The differences with *p* < 0.05 were considered significant.

## 4. Conclusions

We have developed an operationally simple and efficient method for the synthesis of fifteen ammonium quaternary salts (QAs) of bis(pyridinium) by microwave and ultrasounds promoted reactions. The employed conditions furnished remarkably high yields and short reaction times compared to those attained from conventional heating methods. These results confirm the applicability of microwave and ultrasounds heating to the improvement of classical reactions in terms of yields and times. Evaluation of the antibacterial activity showed that the most QAs had reasonable activity against *E. coli*. The most active QAs against *E. coli* were 3, 5, and 15, having MIC value of 0.312 mg/mL. The use of simple tests of seed germination allows assessing the toxic effects of QAs on plants. The toxicity studies of QAs 1, 4, and 7 at two different concentrations (a: 10^−5^ M and b: 10^−6^ M), exhibited no inhibitory effect on wheat seed germination. Since few data are reported about the antimicrobial activity of quaternary ammonium salts of bis(pyridinium), and no study regarding their impact on seed germination of such species was reported so far, we are therefore encouraged to continue our research in this direction.

## Figures and Tables

**Table 1:** Comparative yields and reaction conditions.

QAs	Classical Heating			Microwave Irradiation (Power 300 W)			Ultrasounds Irradiation (35 Hz)		
	Time (min)	Temp. (°C)	Yield (%)	Time (min)	Temp. (°C)	Yield(%)	Time (min)	Temp. (°C)	Yield (%)
**1**	1440	80	92	5–10	95	95	60	60	90
**2**	1440	80	88	5–10	95	90	60	60	90
**3**	1440	80	94	5–10	95	95	60	60	95
**4**	1440	80	95	5–10	95	95	60	60	95
**5**	1440	80	94	5–10	95	94	60	60	95
**6**	1440	80	90	5–10	95	93	60	60	90
**7**	1440	80	82	5–10	95	85	60	60	85
**8**	1440	80	81	5–10	95	83	60	60	90
**9**	720	80	93	5–10	95	95	60	60	90
**10**	720	80	92	5–10	95	95	60	60	90
**11**	1440	80	98	5–10	95	98	60	60	98
**12**	1440	80	85	5–10	95	88	60	60	86
**13**	1440	80	85	5–10	95	87	60	60	90
**14**	720	80	90	5–10	95	92	60	60	93
**15**	720	80	76	5–10	95	77	60	60	80

**Table 2:** Antibacterial activity of QAs against *E. coli*, expressed as minimum inhibitory concentrations (MIC) (mg/mL) determined by the microdilution method.

QAs	Minimum Inhibitory Concentration (MIC) (mg/mL)
**3**	0.312
**5**	0.312
**15**	0.312
**7**	0.625
**9**	0.625
**14**	0.625
**4**	1.25
**10**	1.25
**13**	2.5

## Supplementary Materials

The following are available online, Figures S1–S15: ^1^H-NMR spectra of QAs **1**–**15**, Figures S16–S30, ^13^C-NMR of QAs **1**–**15**, Figures S31–S45, FTIR spectra of QAs **1**–**15**.

## References

[1] Anastas P.T., Kirchhoff M.M. (2002). Origin, current status, and future challenges of green chemistry. Acc. Chem. Res..

[2] Al-Matar H.M., Dawood K.M., Tohamy W.M., Shalaby M.A. (2019). Facile assembling of novel 2,3,6,7,9-pentaazabicyclo[3.3.1]nona-3,7-diene derivatives under microwave and ultrasounds platform. Molecules.

[3] Peng Y., Huang M., Hu Y., Li G., Xia L. (2019). Microwave-assisted synthesis of porphyrin conjugated microporous polymers for microextraction of volatile organic acids in tobaccos. J. Chromatogr. A.

[4] Garella D., Borretto E., Di Stilo A., Martina K., Cravotto G., Cintas P. (2013). Microwave-assisted synthesis of *N*-heterocyles in medicinal chemistry. Med. Chem. Commun..

[5] Verma C., Quraishi M.A., Ebenso E.E. (2018). Microwave and ultrasound irradiations for the synthesis of environmentally sustainable corrosion inhibitors: An overview. Sustain. Chem. Pharm..

[6] Khan S.A., Asiri A.M., Zayed M.E.M., Parveen H., Aqlan F.M.S., Sharma K. (2019). Microwave-assisted synthesis, characterization, and density functional theory study of biologically active ferrocenyl bis-pyrazoline and bispyrimidine as organometallic macromolecules. J. Heterocyclic Chem..

[7] Martins dos Santos D., De Lacerda Bukzem A., Campana-Filho P.S. (2016). Response surface methodology applied to the study of the microwave-assisted synthesis of quaternized chitosan. Carbohydr. Polym..

[8] Aljuhani A., Aouad M.R., Rezki N., Aljaldy O.A., Al-Sodies S.A., Messali M., Ali I. (2019). Novel pyridinium based ionic liquids with amide tethers: Microwave assisted synthesis, molecular docking and anticancer studies. J. Mol. Liq..

[9] Shetgaonkar S.E., Singh F.V. (2019). Ultrasound-assisted one pot synthesis of polysubstituted meta-terphenyls using ring transformation strategy. Synth. Commun..

[10] Madilla S.N., Madilla S., Khumalo M., Bhaskaruni S.V.H.S., Jonnalagadda S.B. (2019). An eco-friendly approach for synthesis of novel substituted 4H-chromenes in aqueous ethanol under ultra-sonication with 94% atom economy. J. Mol. Struct..

[11] Zaoui A., Cherifi Z., Belbachir M. (2019). Ultrasound-induced synthesis of an imidazolium based poly(ionic liquid) inan aqueous media: A structural, thermal and morphological study. Ultrason. Sonochem..

[12] Xue Y., Xiao H., Zhang Y. (2015). Antimicrobial polymeric materials with quaternary ammonium and phosphonium salts. Int. J. Mol. Sci..

[13] Jennings M.C., Minbiole K.P.C., Wuest W.M. (2015). Quaternary ammonium compounds: An antimicrobial mainstay and platform for innovation to address bacterial resistance. ACS Infect. Dis..

[14] Abel T., Cohen J.I., Engel R., Filshtinskaya M., Melkonian A., Melkonian K. (2002). Preparation and investigation of antibacterial carbohydrate-based surfaces. Carbohydr. Res..

[15] Dizman B., Elasri M.O., Mathias L.J. (2004). Synthesis and antimicrobial activities of new water-soluble bis-quaternary ammonium methacrylate polymers. J. Appl. Polym. Sci..

[16] Lenoir S., Pagnoulle C., Detrembleur C., Galleni M., Jérôme R. (2006). New antibacterial cationic surfactants prepared by atom transfer radical polymerization. J. Polym. Sci. A: Polym. Chem..

[17] Furdui B., Parfene G., Ghinea I.O., Dinica R.M., Bahrim G., Demeunynck M. (2014). Synthesis and in vitro antimicrobial evaluation of new N-heterocyclic diquaternary pyridinium compounds. Molecules.

[18] Brannan D.K. (1997). Cosmetic Microbiology: A Practical Handbook.

[19] McBain A.J., Ledder R.G., Moore L.E., Catrenich C.E., Gilbert P. (2004). Effects of Quaternary-ammonium-based formulations on bacterial community dynamics and antimicrobial susceptibility. Appl. Environ. Microbiol..

[20] Fauci A.S., Touchette N.A., Folkers G.K. (2005). Emerging infectious diseases: A 10-year perspective from the National Institute of Allergy and Infectious Diseases. Emerg. Infect. Dis..

[21] Fan Z., Senapati D., Khan S.A., Singh A.K., Hamme A., Yust B., Sardar D., Ray P.C. (2013). Popcorn-shaped magnetic core-plasmonic shell multifunctional nanoparticles for the targeted magnetic separation and enrichment, label-free SERS imaging, and photothermal destruction of multidrug-resistant bacteria. Chem. Eur. J..

[22] Boucher H.W., Talbot G.H., Bradley J.S., Edwards J.E., Gilbert D., Rice L.B., Scheld M., Spellberg B., Bartlett J. (2009). Bad bugs, no drugs: No ESKAPE! An update from the Infectious Diseases Society of America. Clin. Infect. Dis..

[23] Álvarez-Paino M., Muñoz-Bonilla A., López-Fabal F., Gómez-Garcés J.L., Heuts J.P.A., Fernández-García M. (2015). Effect of glycounits on the antimicrobial properties and toxicity behavior of polymers based on quaternized DMAEMA. Biomacromolecules.

[24] Furdui B., Dinica R.M., Tabacaru A., Pettinari C. (2012). Synthesis and physico-chemical properties of a novel series of aromatic electron acceptors based on *N*-heterocycles. Tetrahedron.

[25] Elgemeie G.H., Mohamed R.A. (2018). Microwave synthesis of fluorescent and luminescent dyes (1990–2017). J. Mol. Struct..

[26] Vera G., Diethelm B., Terraza C.A., Recabarren-Gajardo G. (2018). Suzuki-type cross-coupling reaction of unprotected 3-iodoindazoles with pinacol vinyl boronate: An expeditive C-3 vinylation of indazoles under microwave irradiation. Molecules.

[27] Banerjee B. (2017). Recent developments on ultrasound assisted catalyst-free organic synthesis. Ultrasonics Sonochemistry.

[28] Pawełczyk A., Sowa-Kasprzak K., Olender D., Zaprutko L. (2018). Microwave (MW), ultrasound (US) and combined synergic MW-US strategies for rapid functionalization of pharmaceutical use phenols. Molecules.

[29] Ohta Y., Kondo Y., Kawada K., Teranaka T., Yoshino N. (2008). Synthesis and antibacterial activity of quaternary ammonium salt-type antibacterial agents with a phosphate group. J. Oleo Sci..

[30] Alptüzün V., Parlar S., Taşlı H., Erciyas E. (2009). Synthesis and antimicrobial activity of some pyridinium salts. Molecules.

[31] Liang Z., Zhu M., Yang Y.-W., Gao H. (2014). Antimicrobial activities of polymeric quaternary ammonium salts from poly(glycidyl methacrylate)s. Polym. Adv. Technol..

[32] Ellof J.N. (1998). A sensitive and quick microplate method to determine the minimal inhibitory concentration of plant extracts for bacteria. Planta Med..

[33] Abate G., Aseffa A., Selassie A., Goshu S., Fekade B., WoldeMeskal D., Miörner H. (2004). Direct colorimetric assay for rapid detection of rifampin-resistant Mycobacterium tuberculosis. J. Clin. Microbiol..

[34] Altman F.P. (1976). Tetrazolium salts and formazans. Prog. Histochem. Cytochem..

[35] Thom S.M., Horobin R.W., Seidler E., Barer M.R. (1993). Factors affecting the selection and use of tetrazolium salts as cytochemical indicators of microbial viability and activity. J. Appl. Bacteriol..

[36] Caviedes L., Delgado J., Gilman R.H. (2002). Tetrazolium microplate assay as a rapid and inexpensive colorimetric method for determination of antibiotic susceptibility of Mycobacterium tuberculosis. J. Clin. Microbiol..

[37] Yamane N., Oiwa T., Kiyota T., Saitoh H., Sonoda T., Tosaka M., Nakashima M., Fukunaga H., Masaki T., Miyagawa K. (1996). Multicenter evaluation of a colorimetric microplate antimycobacterial susceptibility test: comparative study with the NCCLS M24-P. Rinsho Byori.

[38] Klančnik A., Piskernik S., Jeršek B., Možina S.S. (2010). Evaluation of diffusion and dillution methods to determine the antibacterial activity of plant extracts. J. Microbiol. Methods.

[39] Moussa S.H., Tayel A.A., Al-Hassan A.A., Farouk A. (2013). Tetrazolium/formazan test as an efficient method to determine fungal chitosan antimicrobial activity. J. Mycol..

[40] Ol’khovik V.K., Matveienko Y.V., Vasilevskii D.A., Kalechits G.V., Zheldakova R.A. (2003). Synthesis, antimicrobial and antifungal activity of double quaternary ammonium salts of biphenyls. Russ. J. Gen. Chem..

[41] Gilbert P., Al-taae A. (1985). Antimicrobial activity of some alkyltrimethylammonium bromides. Lett. Appl. Microbiol..

[42] Gupta D., Bhatia D., Dave V., Sutariya V., Gupta S.V. (2018). Salts of therapeutic agents: chemical, physicochemical, and biological considerations. Molecules.

[43] Mohammed M., Tahar B., Aicha D., Eddine H.D. (2010). Antibacterial activity of quaternary ammonium salt from diethylaminoethyl methacrylate. E-J. Chem..

[44] Marini M., Bondi M., Iseppi R., Toselli M., Pilati F. (2007). Preparation and antibacterial activity of hybrid materials containing quaternary ammonium salts via sol–gel process. Eur. Polym. J..

[45] Zhou L., Xia M., Wang L., Mao H. (2016). Toxic effect of perfluorooctanoic acid (PFOA) on germination and seedling growth of wheat (*Triticum aestivum* L.). Chemosphere.

[46] Izabela J., Patryk O., Ewa S. (2017). Toxicity of combined mixtures of nanoparticles to plants. J. Hazardous Mater..

[47] Belz R.G., Hurle K., Duke S.O. (2005). Dose-response—A challenge for allelopathy?. Nonlinearity Biol. Toxicol Med..

[48] Khaliq A., Matloob A.I., Aslam F., Bismillah Khan M. (2011). Influence of wheat straw and rhizosphere on seed germination, early seedling growth and bio-chemical attributes of *Trianthema portulacastrum*. Planta Daninha.

[49] Luo Y., Liang J., Zeng G., Chen M., Mo D., Li G., Zhang D. (2018). Seed germination test for toxicity evaluation of compost: Its roles, problems and prospects. Waste Management.

[50] Tiquia S.M., Tam N.F.Y., Hodgkiss I.J. (1996). Effects of composting on phytotoxicity of spent pig-manure sawdust litter. Environ. Pollut..

[51] Oke F., Aslim B., Ozturk S., Altundag S. (2009). Essential oil composition, antimicrobial and antioxidant activities of Satureja cuneifolia Ten. Food Chem..

[52] Pan M., Chu L.M. (2016). Phytotoxicity of veterinary antibiotics to seed germination and root elongation of crops. Ecotoxicol. Environ. Saf..

