# Supplementary material for: Green Accelerated Synthesis, Antimicrobial Activity and Seed Germination Test of Quaternary Ammonium Salts of 1,2-bis(4-pyridyl)ethane
Source: Molecules. 2019 Jul 1;24(13):2424. doi: 10.3390/molecules24132424 (PMC6651484; doi:10.3390/molecules24132424)

Supplementary information
for the manuscript

Green accelerated synthesis of quaternary ammonium salts with antimicrobial activity and an assessment on their impact on seed germination

Aurel Tăbăcaru, Andreea Veronica Dediu (Botezatu), Georgiana Horincar,
Bianca Furdui* and Rodica Mihaela Dinică*

Figure S1. ^{1}H-NMR spectrum of compound **1**

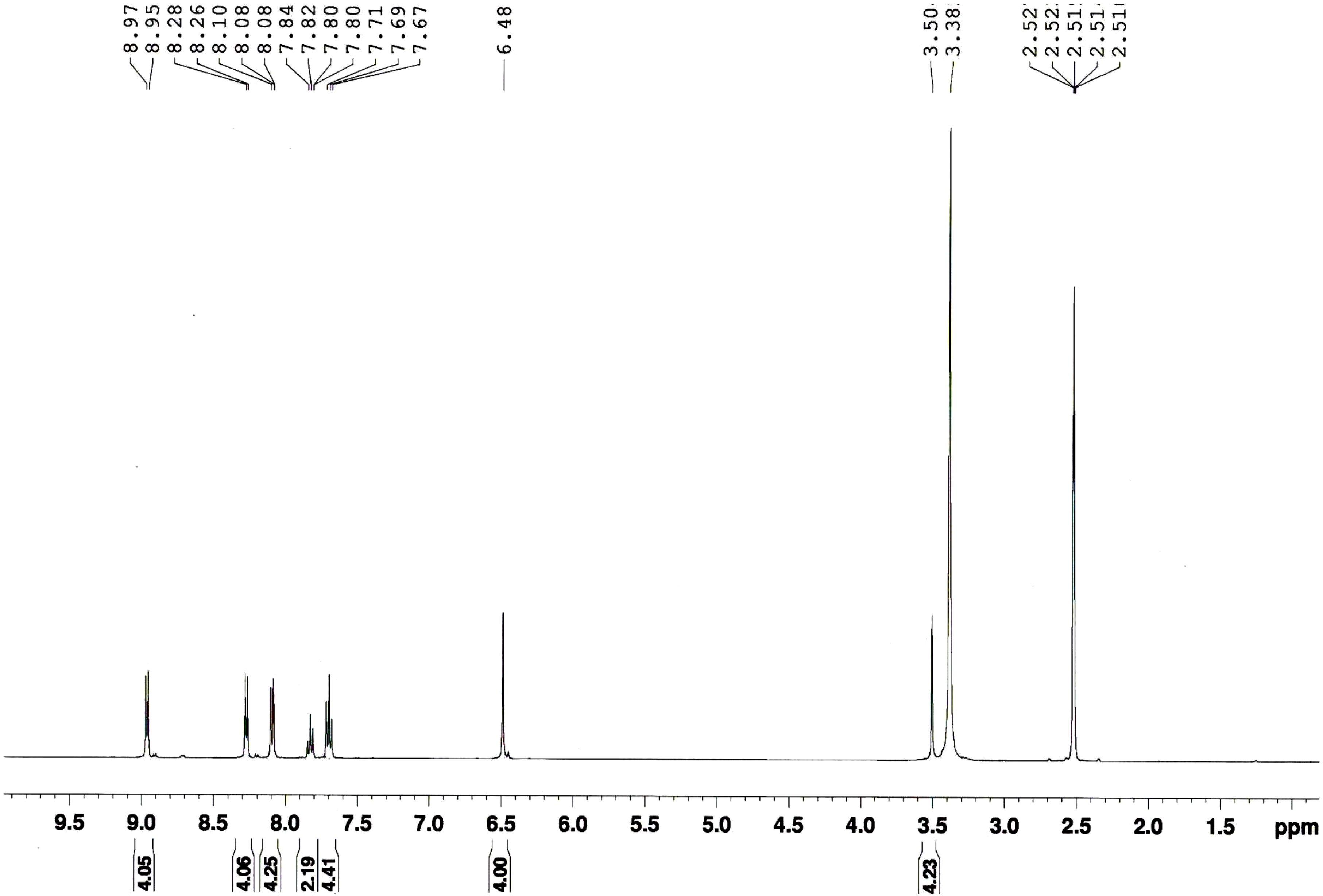

Figure S2. ^{13}C-NMR spectrum of compound **1**

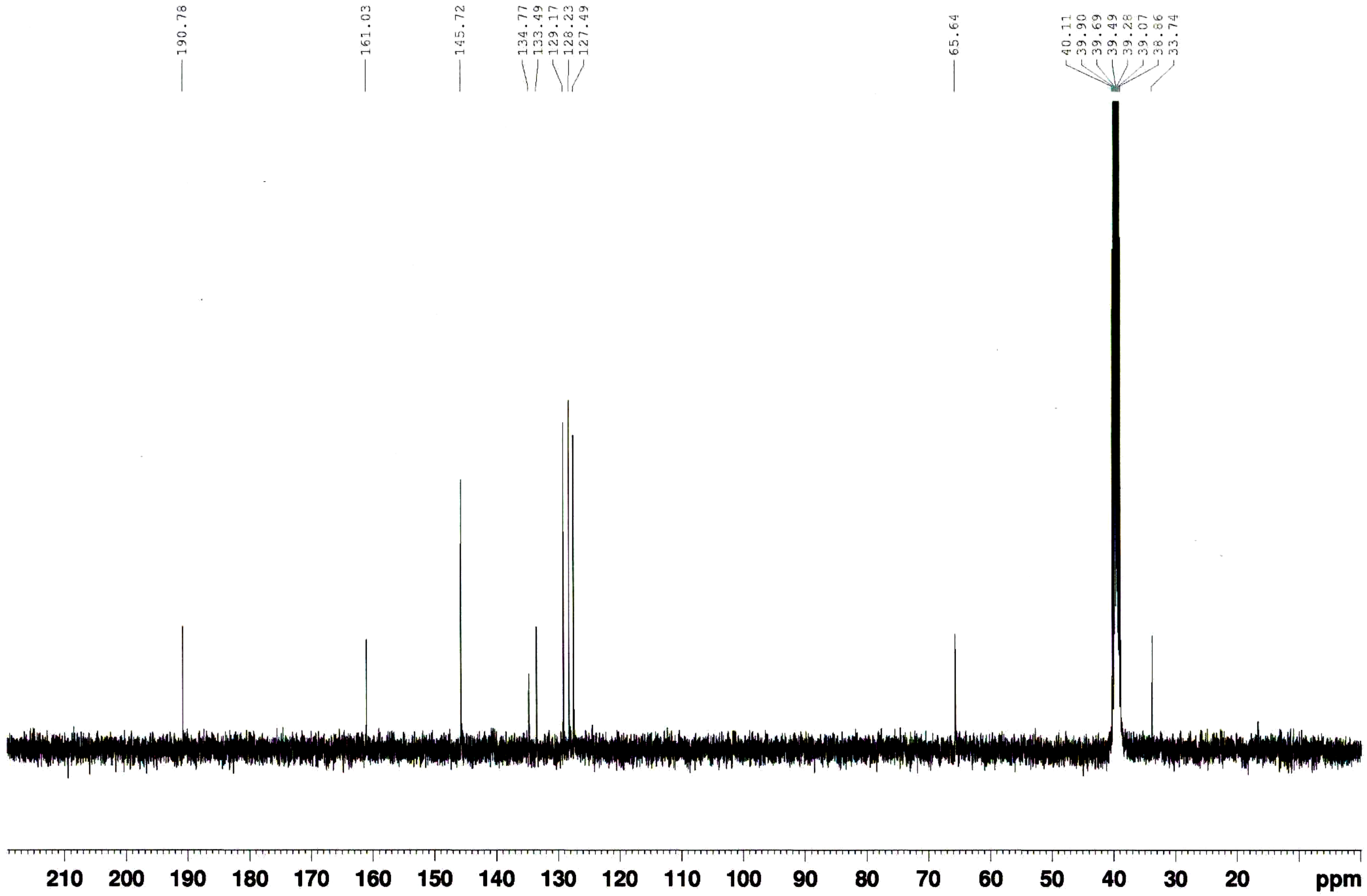

Figure S3. ^{1}H-NMR spectrum of compound **2**

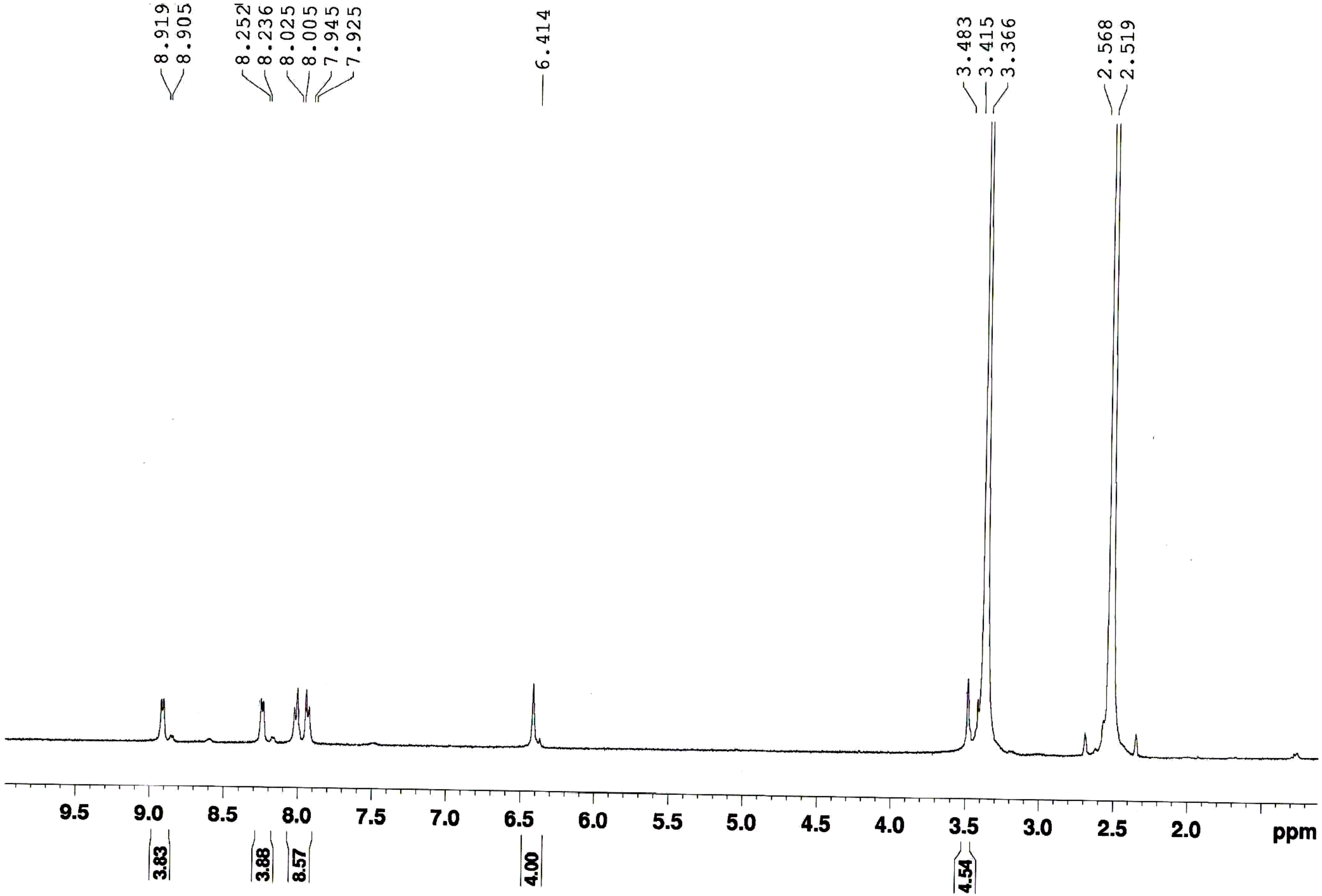

Figure S4. ^{13}C-NMR spectrum of compound **2**

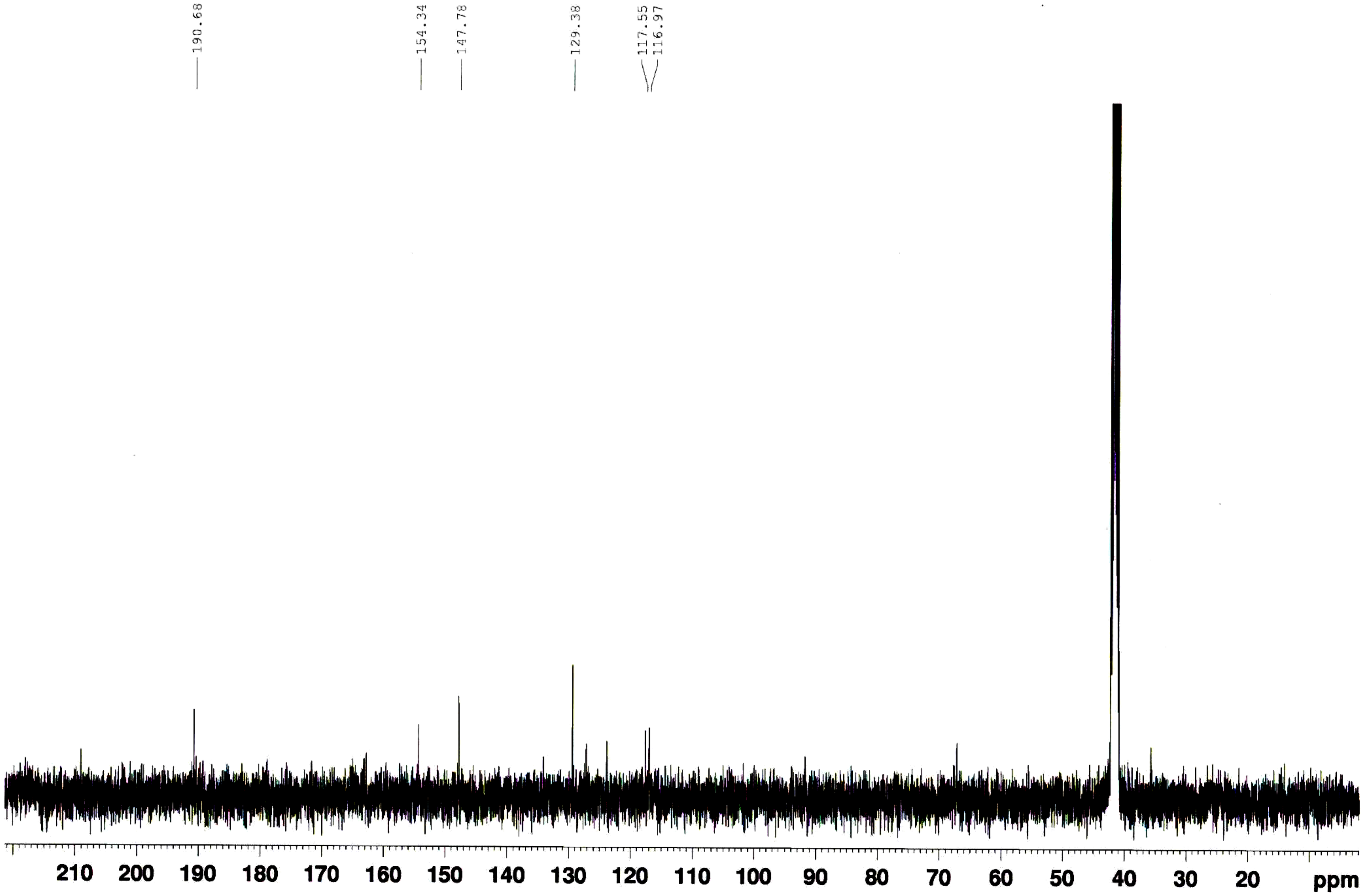

Figure S5. ^{1}H-NMR spectrum of compound **3**

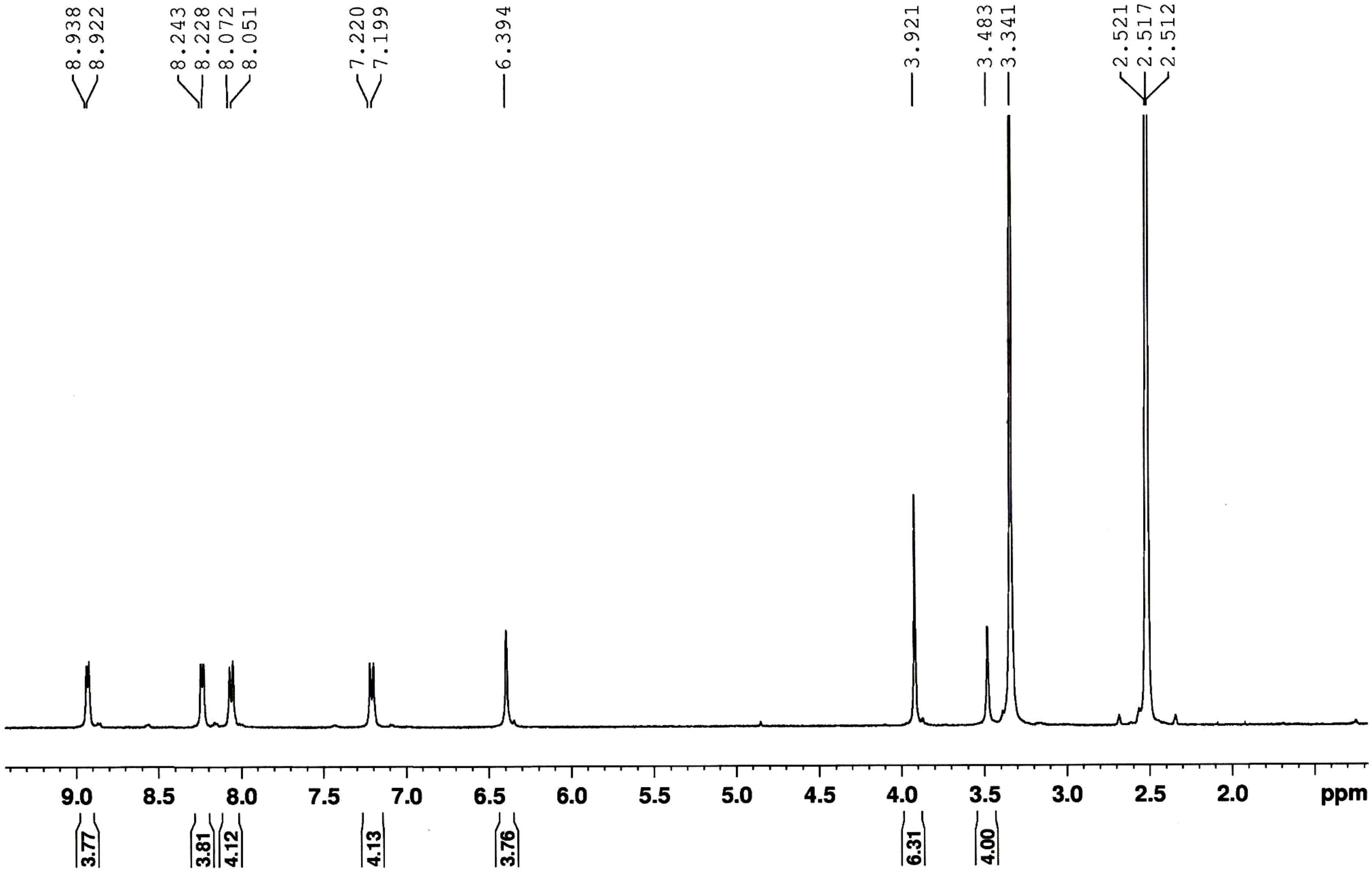

Figure S6. ^{13}C-NMR spectrum of compound **3**

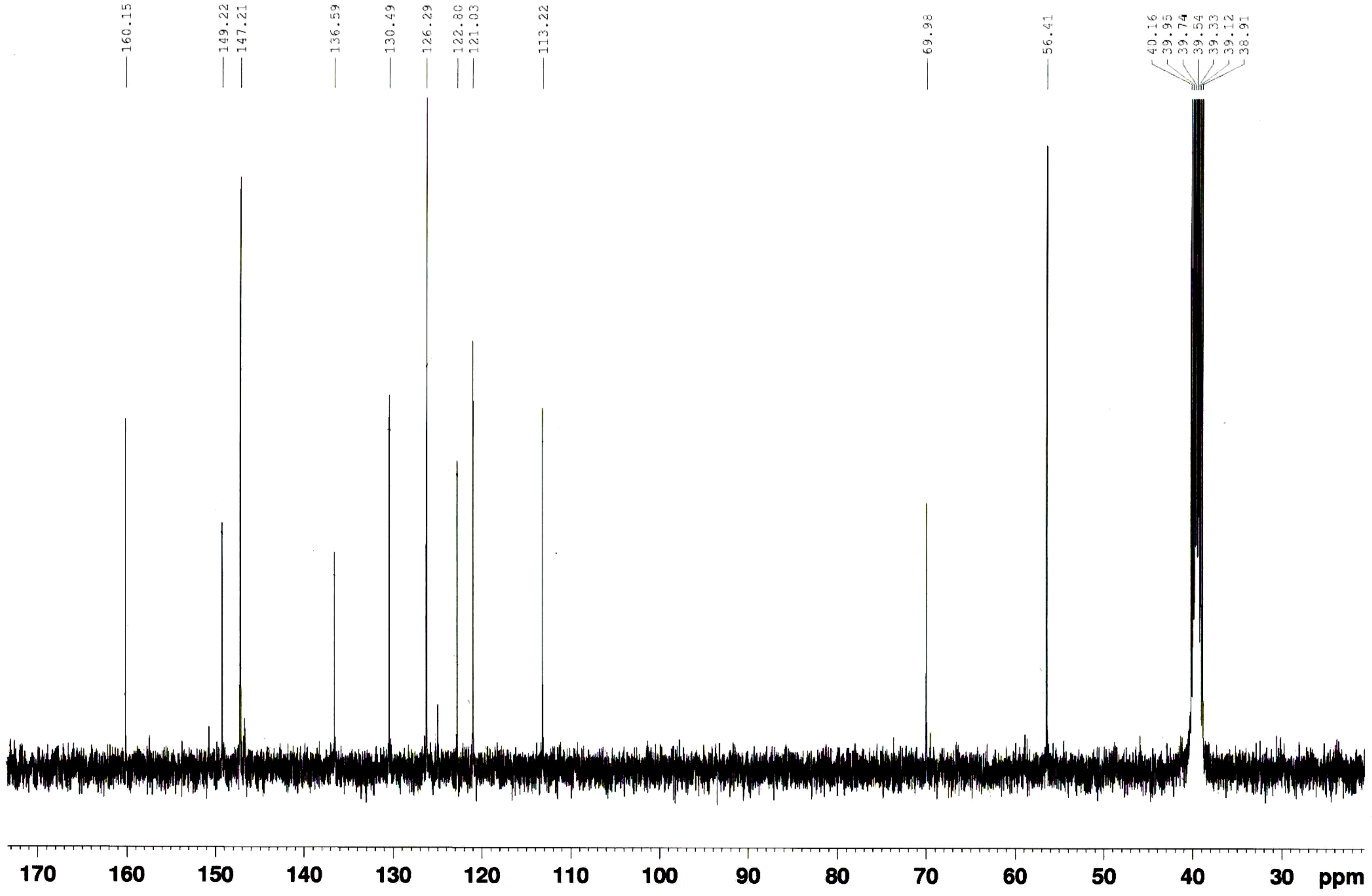

Figure S7. ^{1}H-NMR spectrum of compound **4**

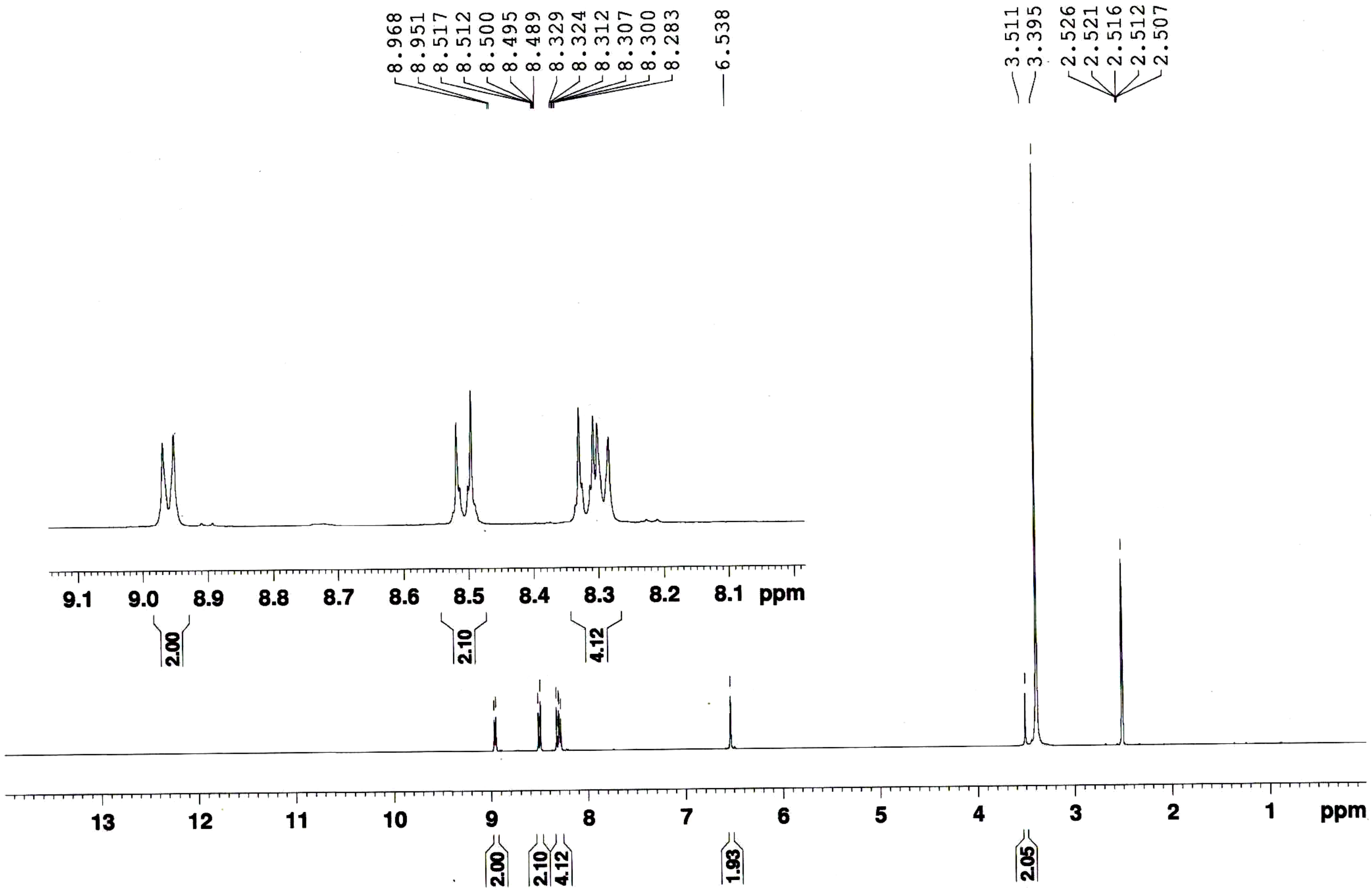

Figure S8. ^{13}C-NMR spectrum of compound **4**

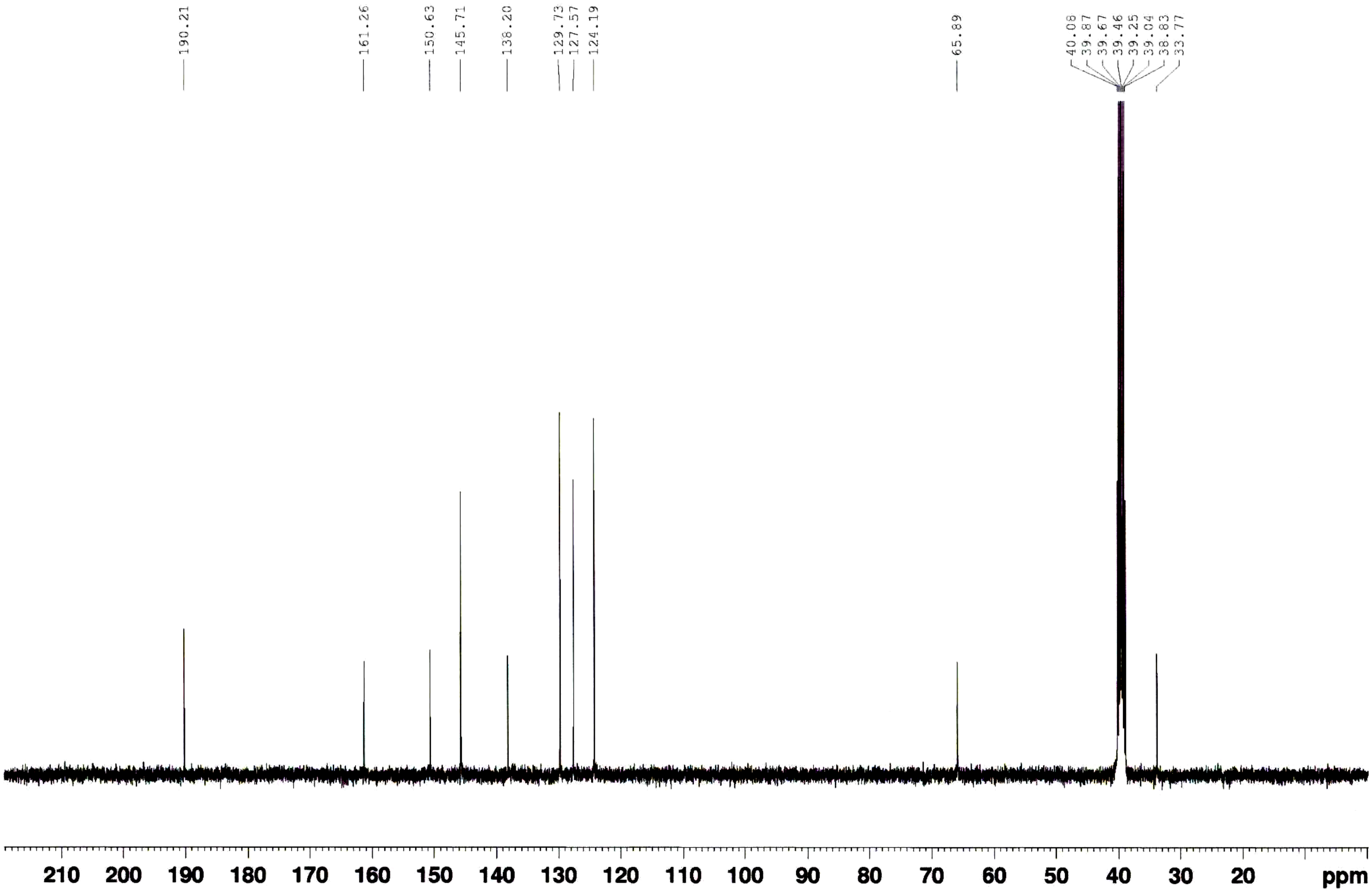

Figure S9. ^{1}H-NMR spectrum of compound **5**

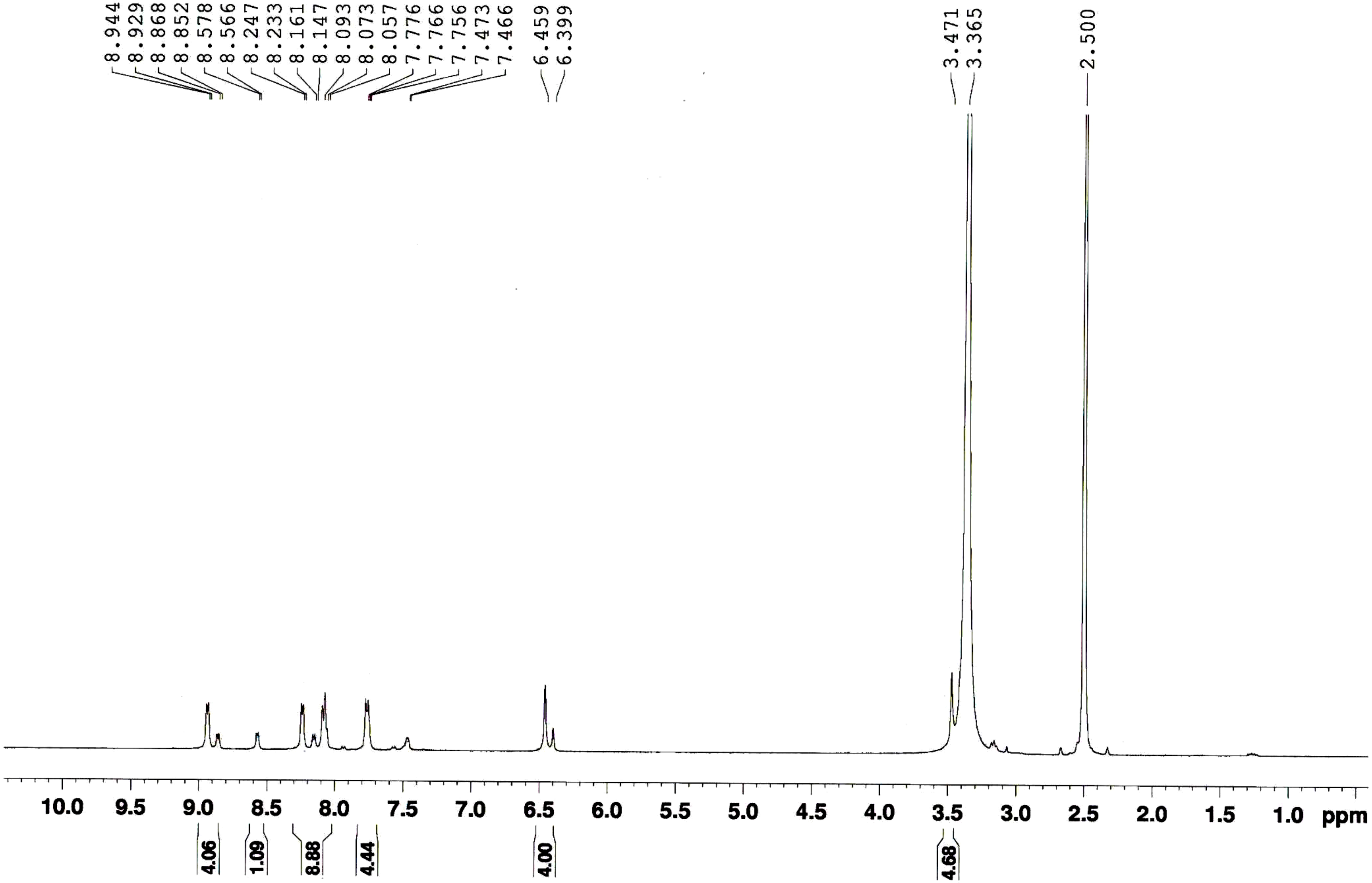

Figure S10. ^{13}C-NMR spectrum of compound **5**

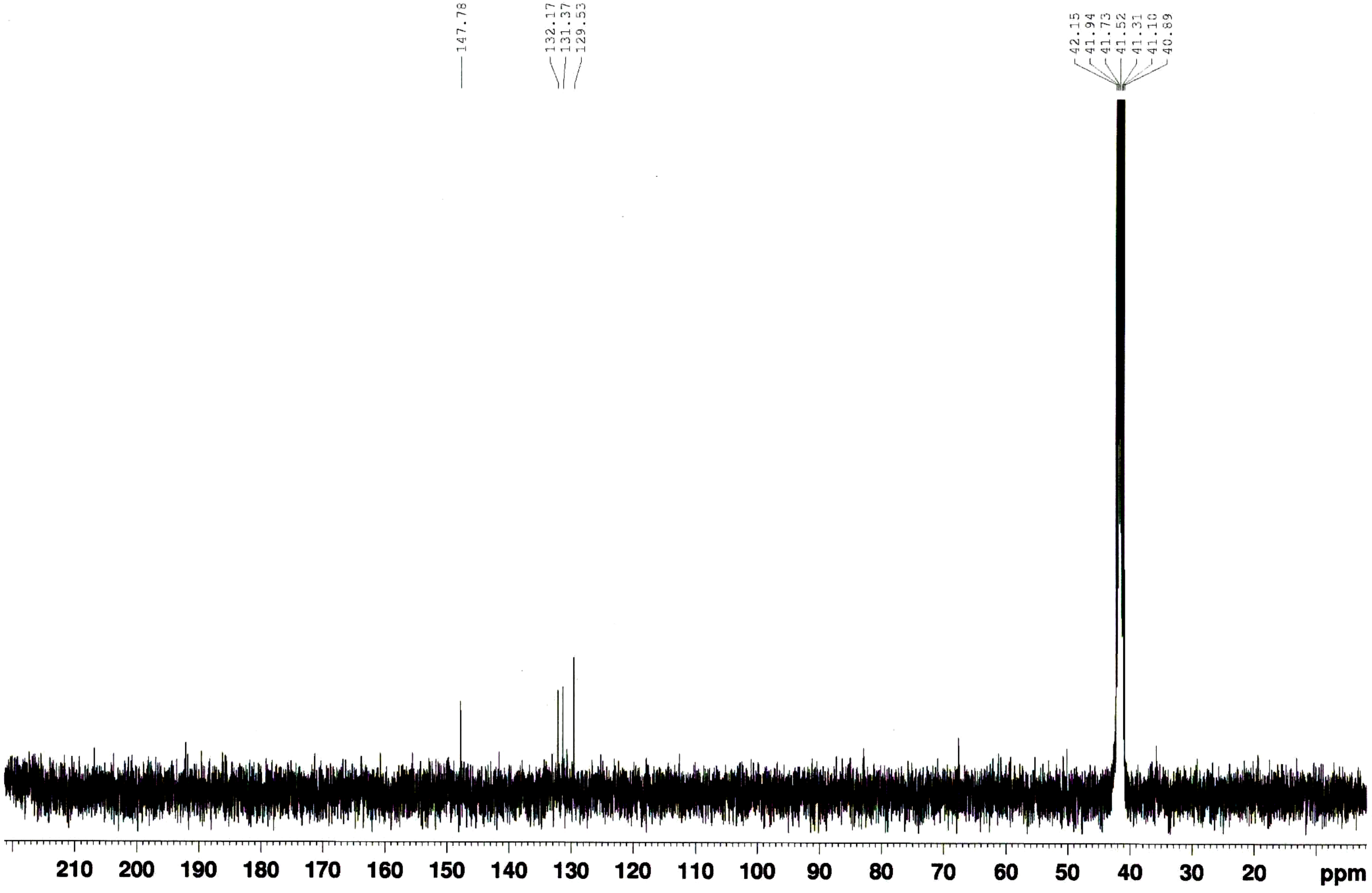

Figure S11. ^{1}H-NMR spectrum of compound **6**

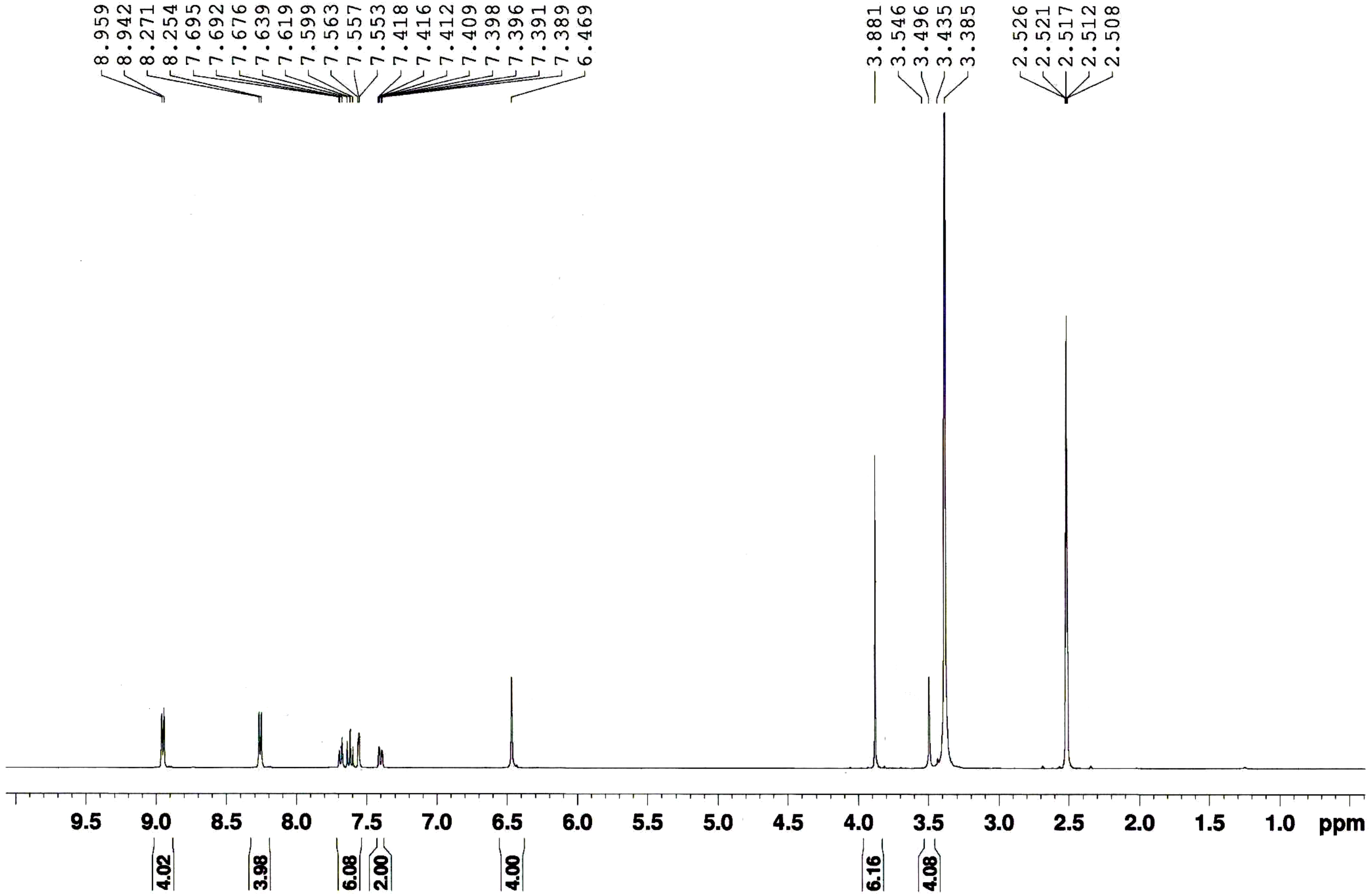

Figure S12. ^{13}C-NMR spectrum of compound **6**

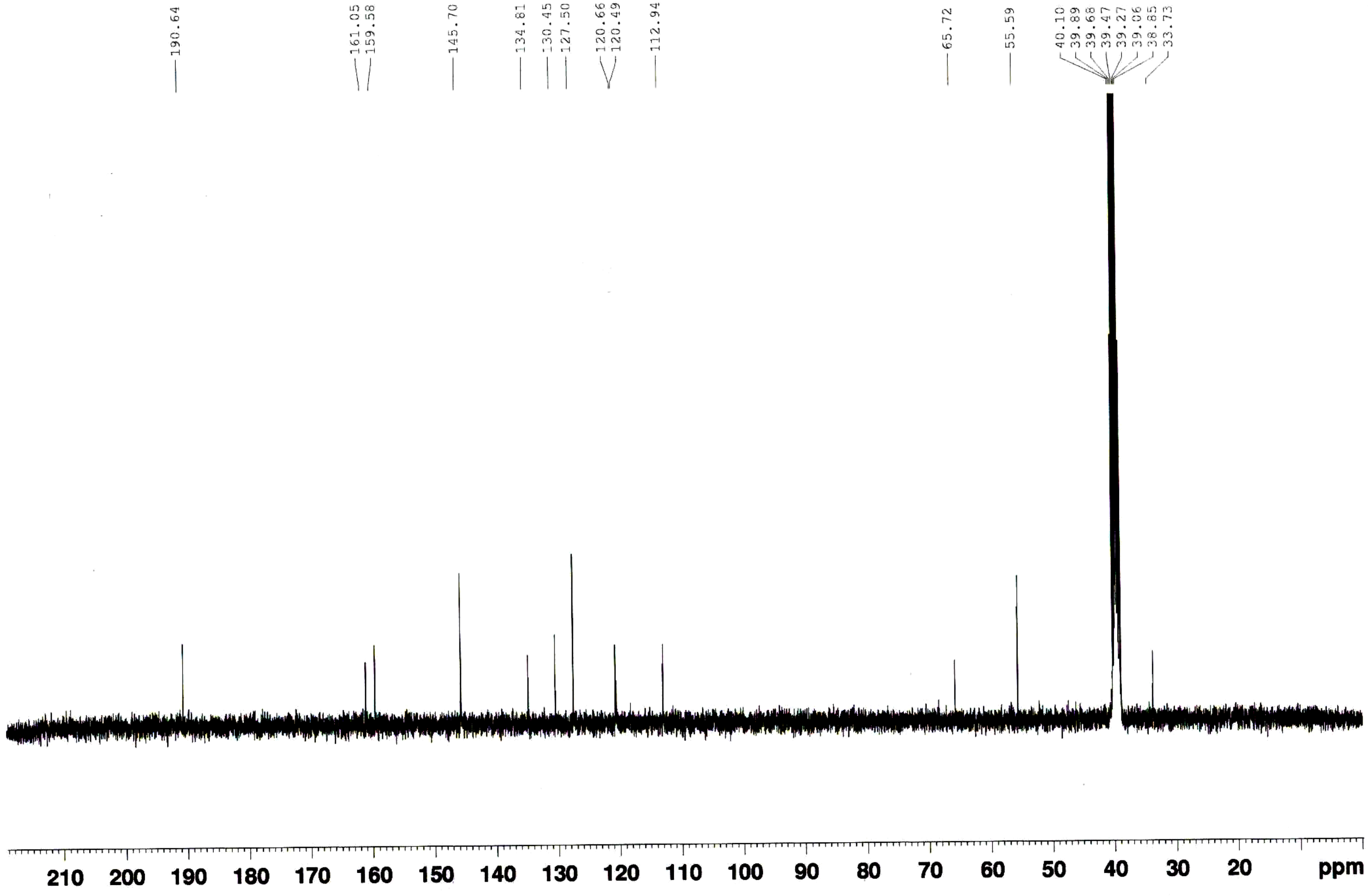

Figure S13. ^{1}H-NMR spectrum of compound **7**

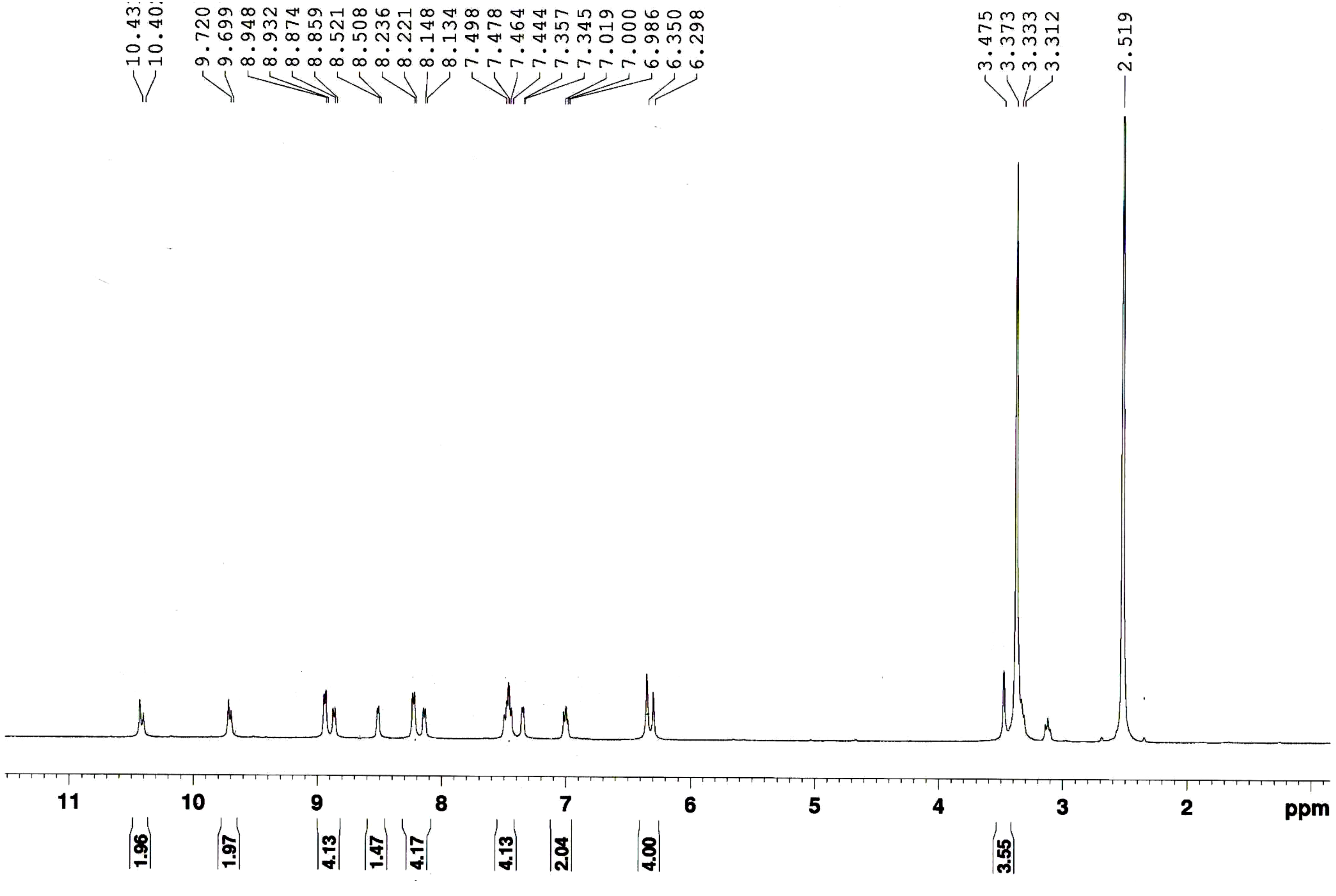

Figure S14. ^{13}C-NMR spectrum of compound **7**

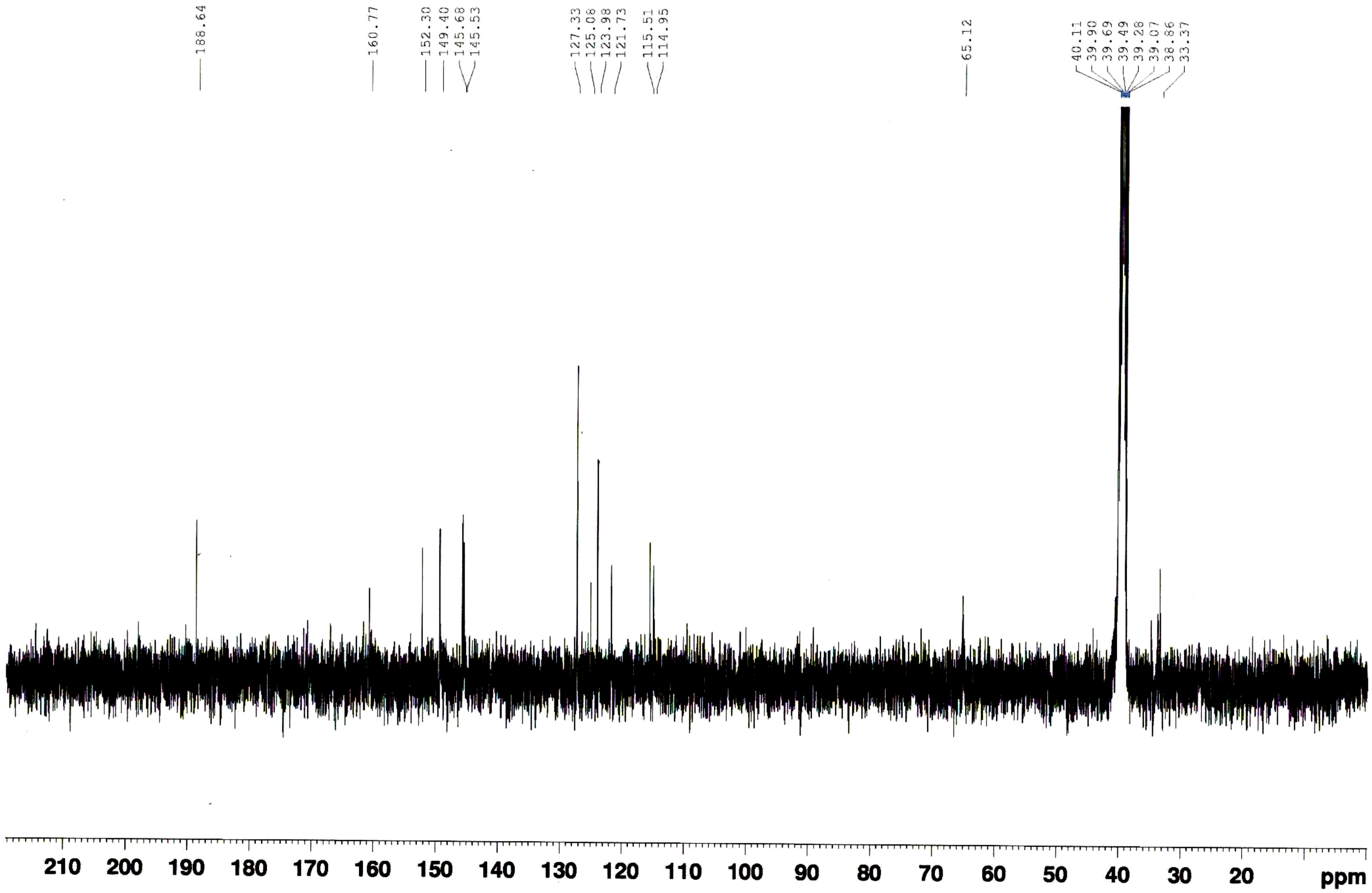

Figure S15. ^{1}H-NMR spectrum of compound **8**

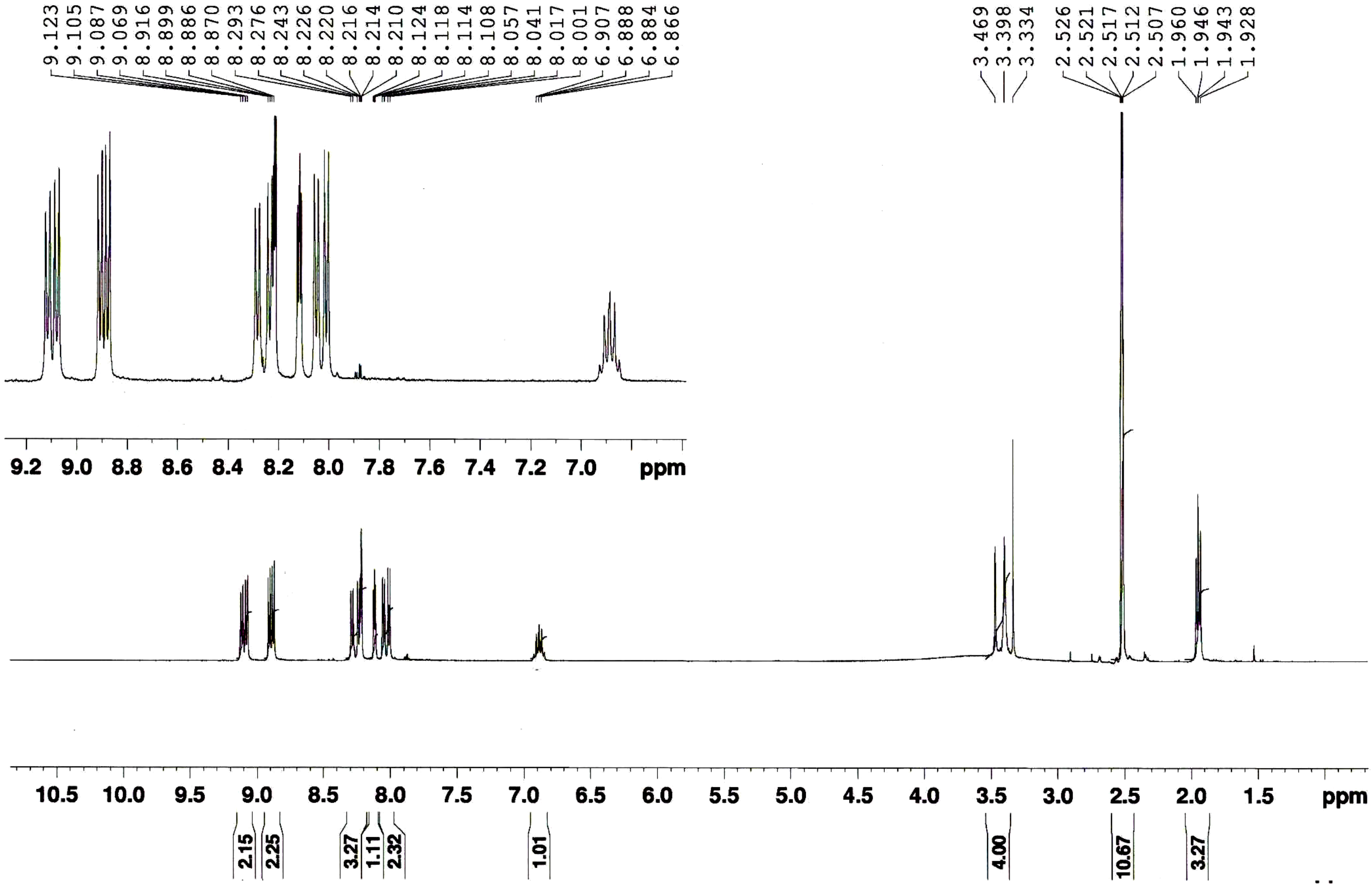

Figure S16. ^{13}C-NMR spectrum of compound **8**

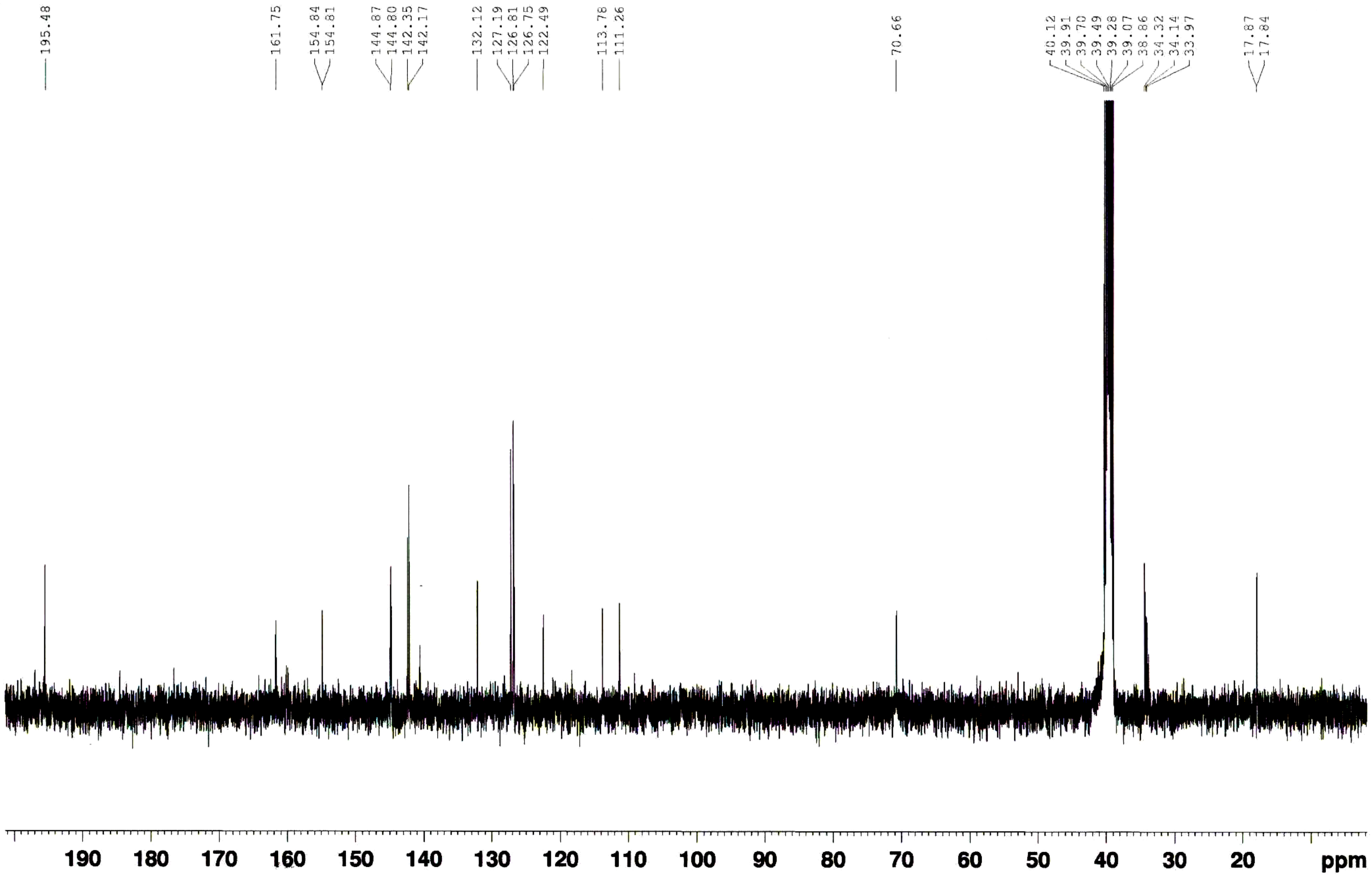

Figure S17. ^{1}H-NMR spectrum of compound **9**

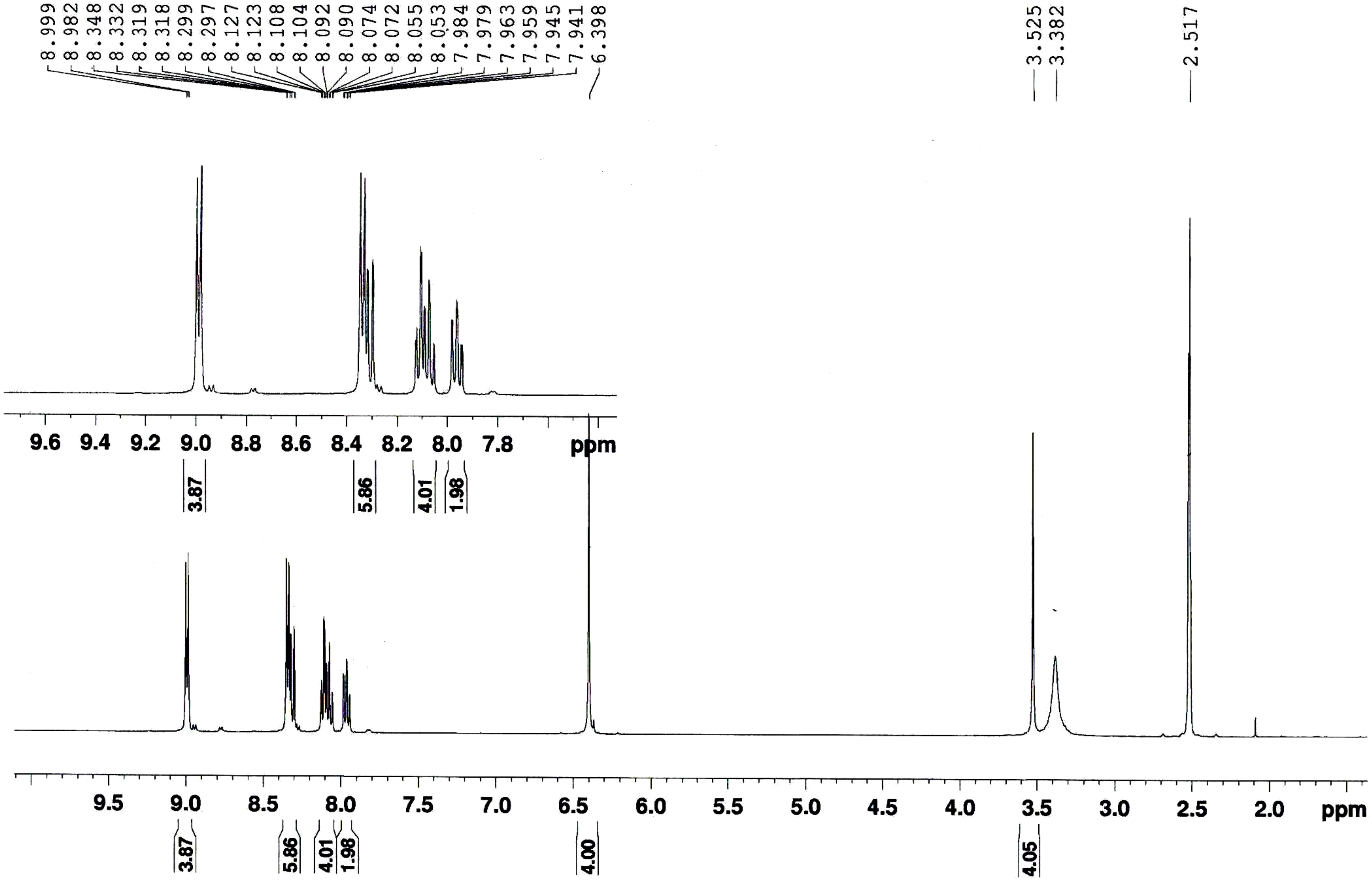

Figure S18. ^{13}C-NMR spectrum of compound **9**

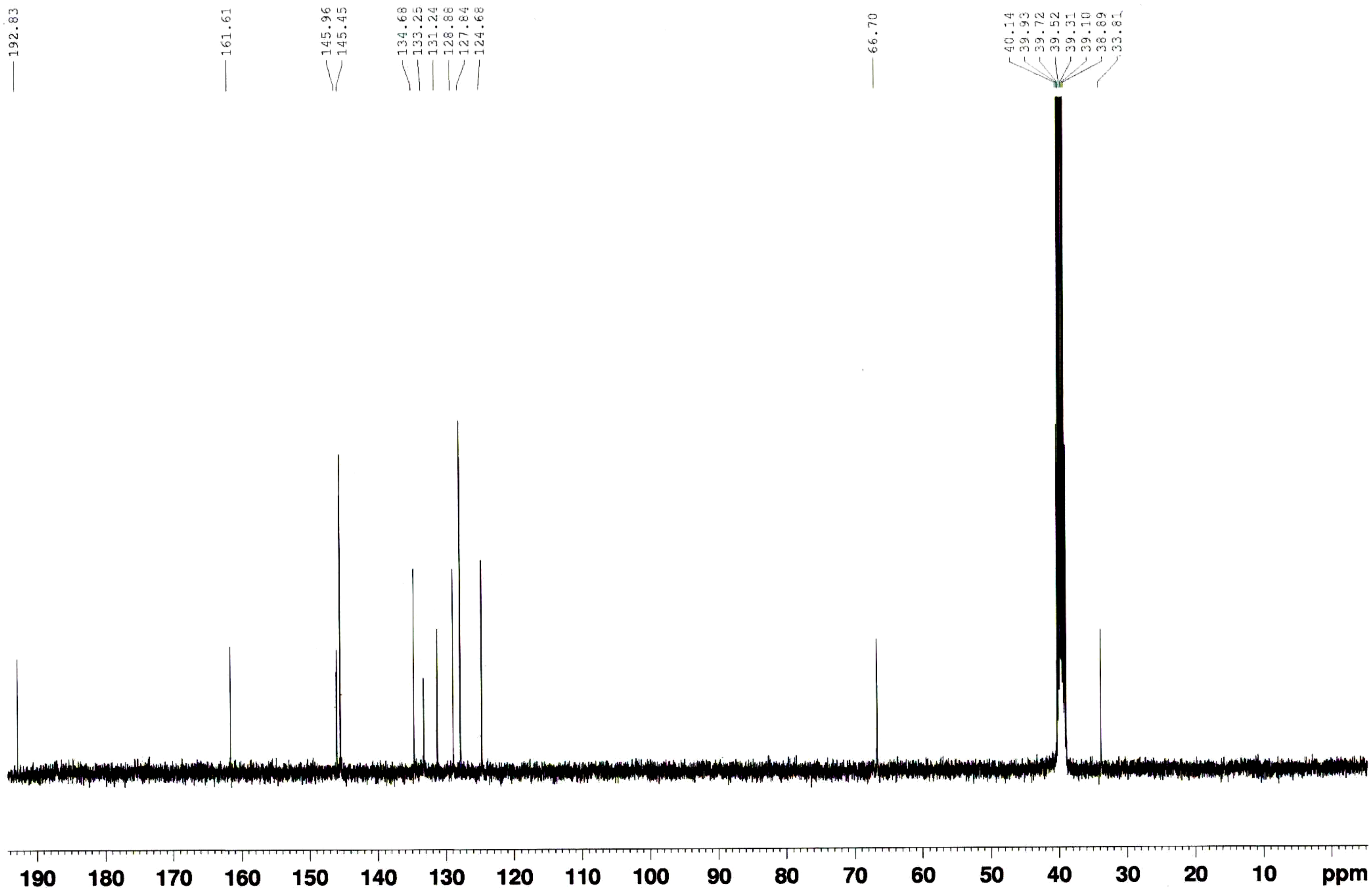

Figure S19. ^{1}H-NMR spectrum of compound **10**

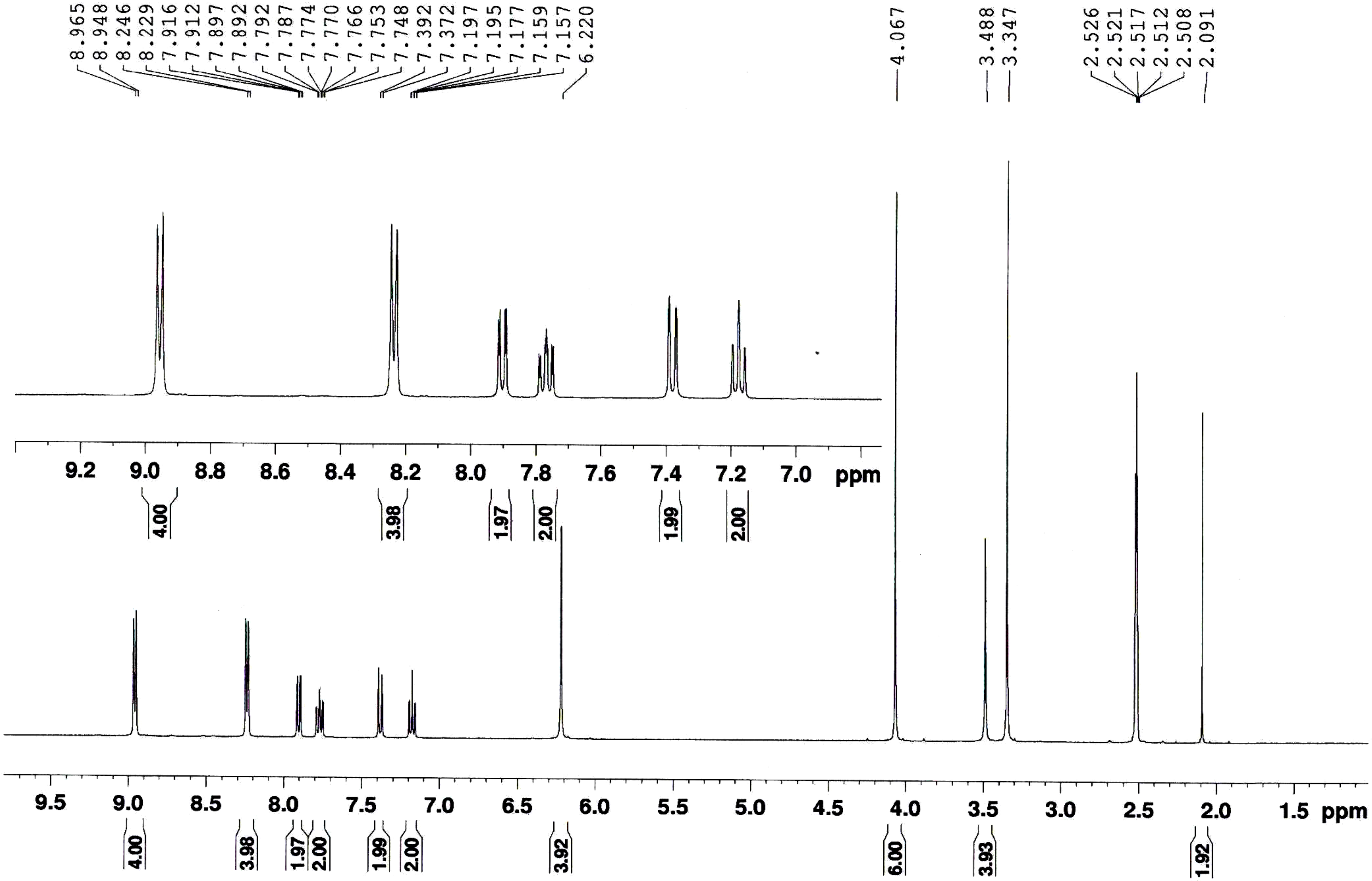

Figure S20. ^{13}C-NMR spectrum of compound **10**

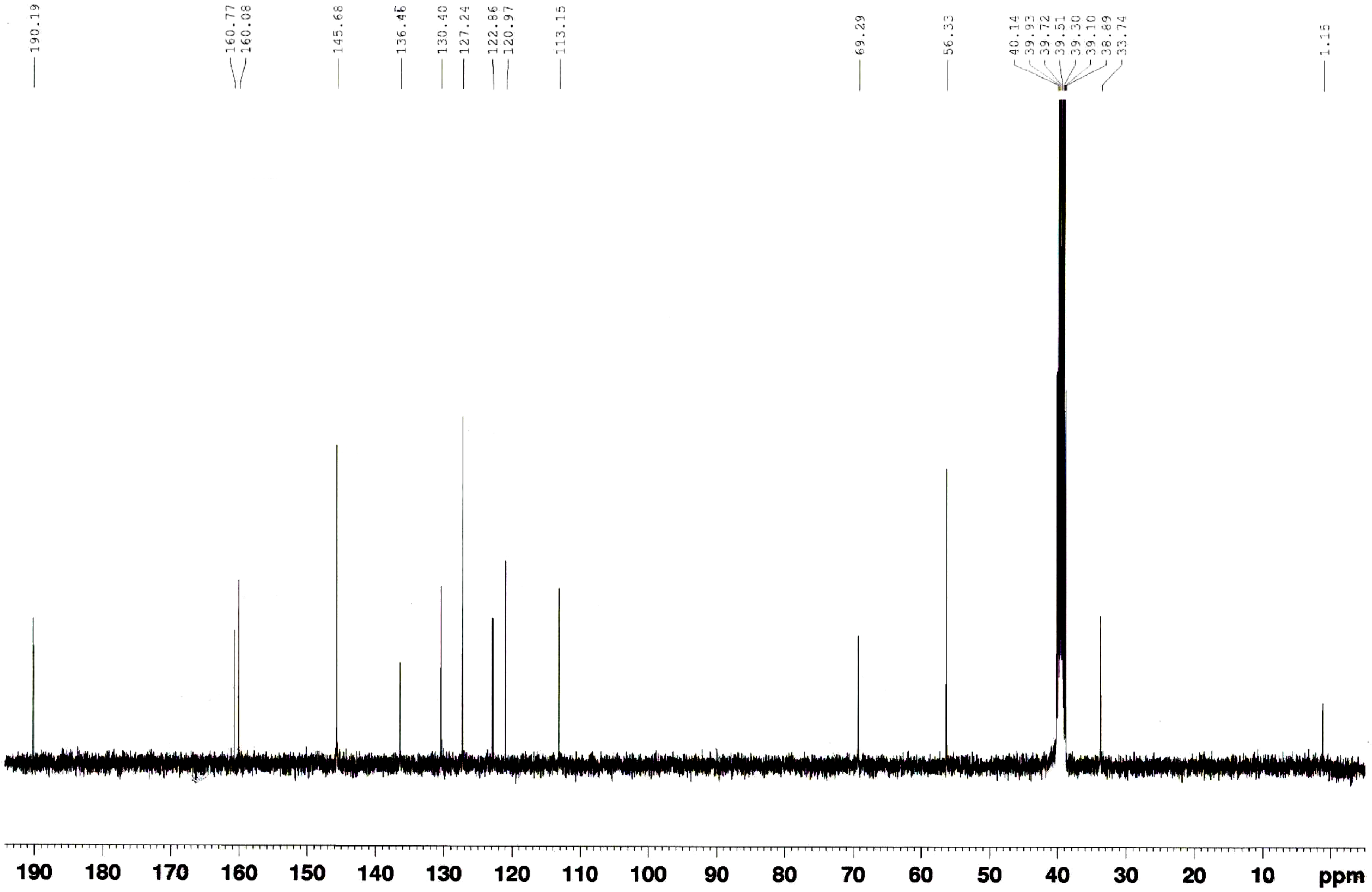

Figure S21. ^{1}H-NMR spectrum of compound **11**

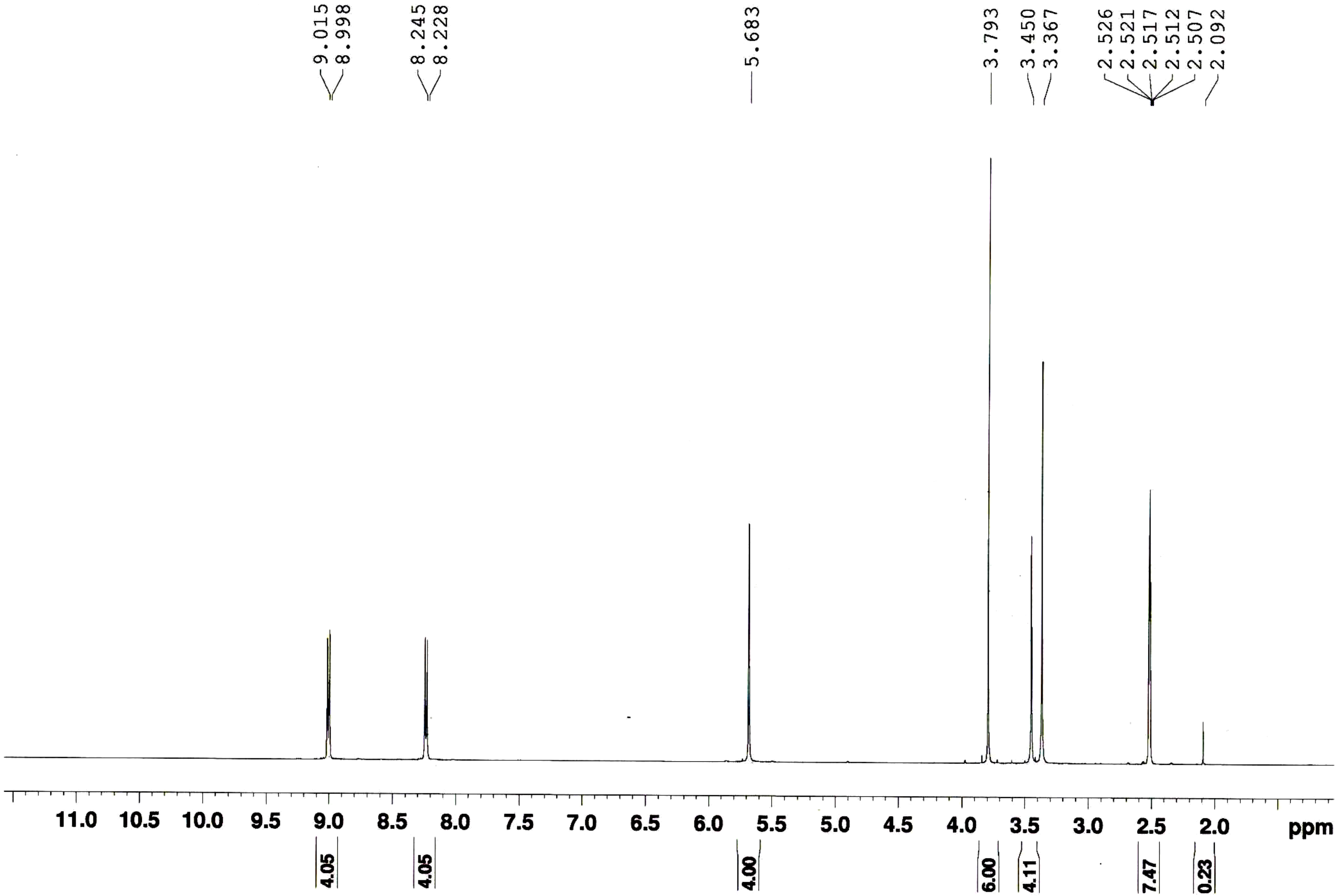

Figure S22. ^{13}C-NMR spectrum of compound **11**

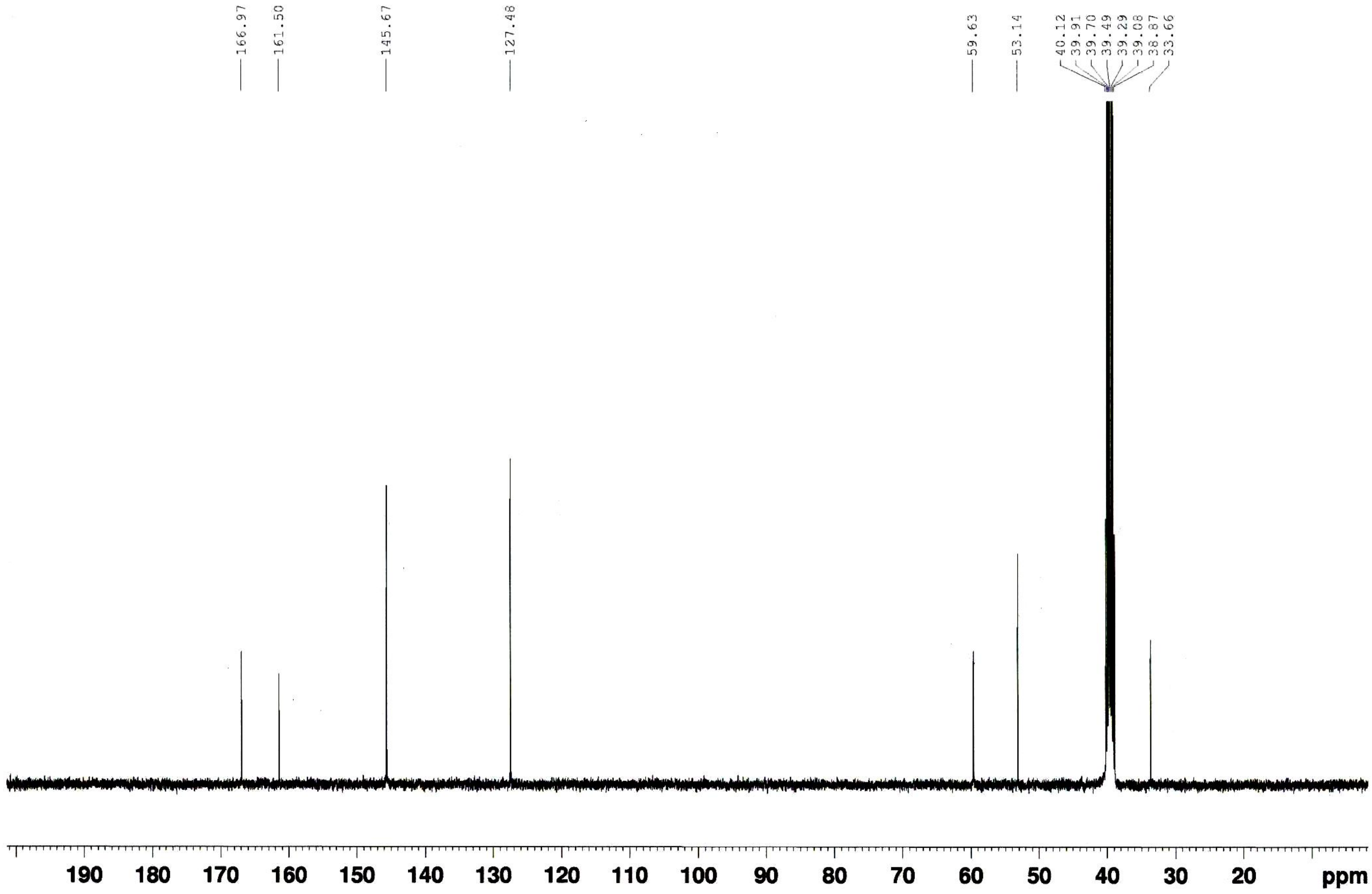

Figure S23. ^{1}H-NMR spectrum of compound **12**

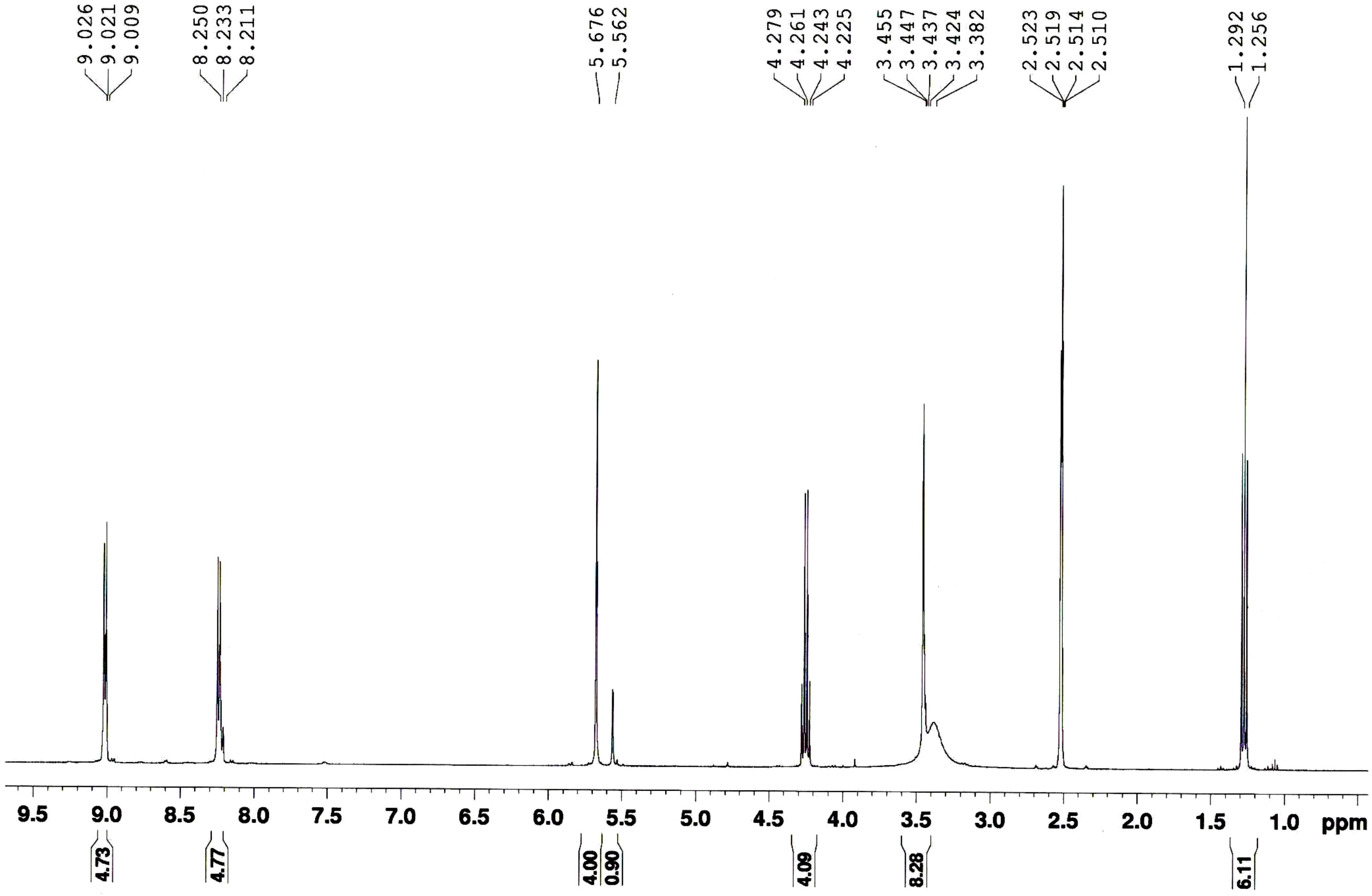

Figure S24. ^{13}C-NMR spectrum of compound **12**

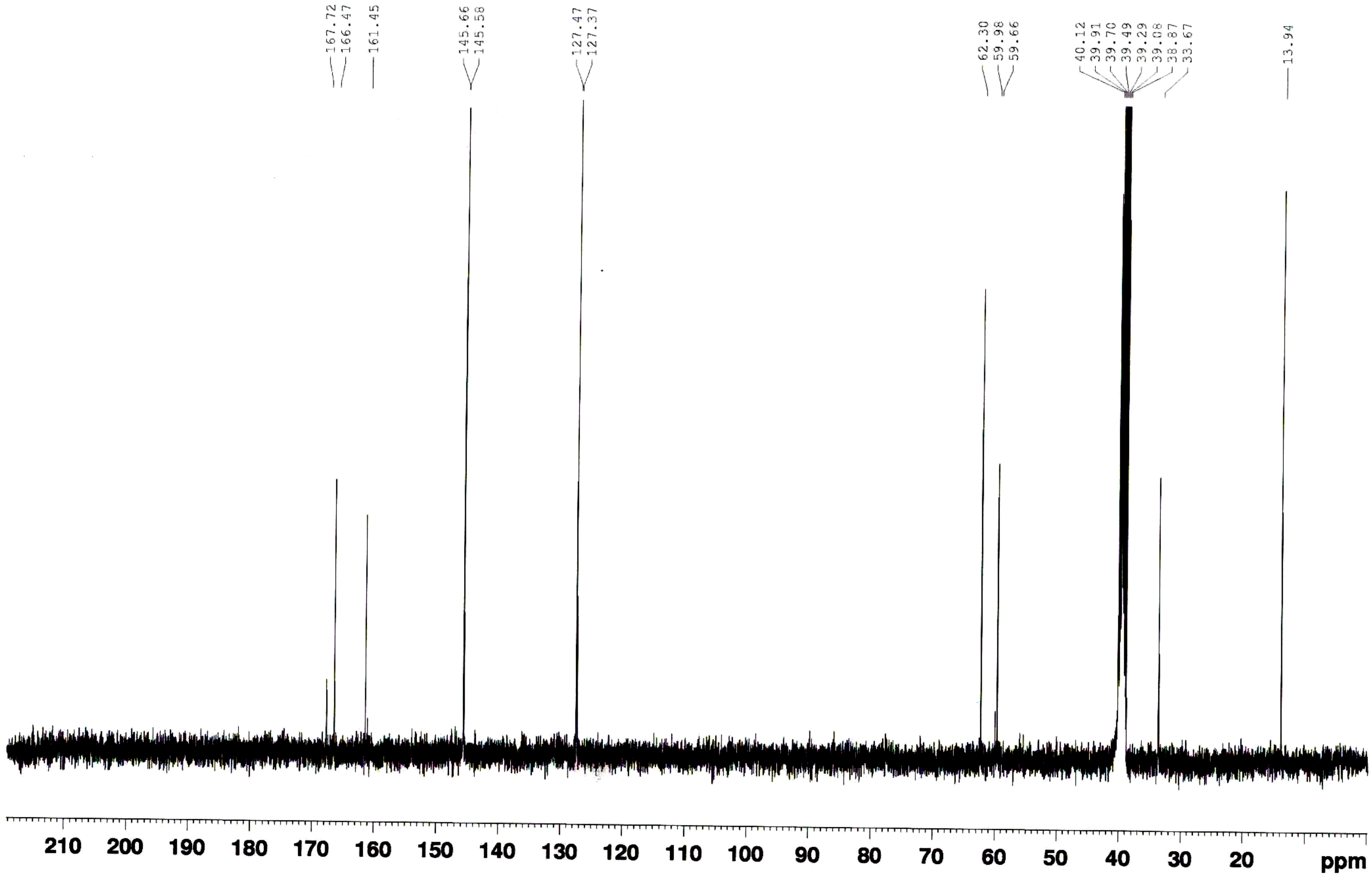

Figure S25. ^{1}H-NMR spectrum of compound **13**

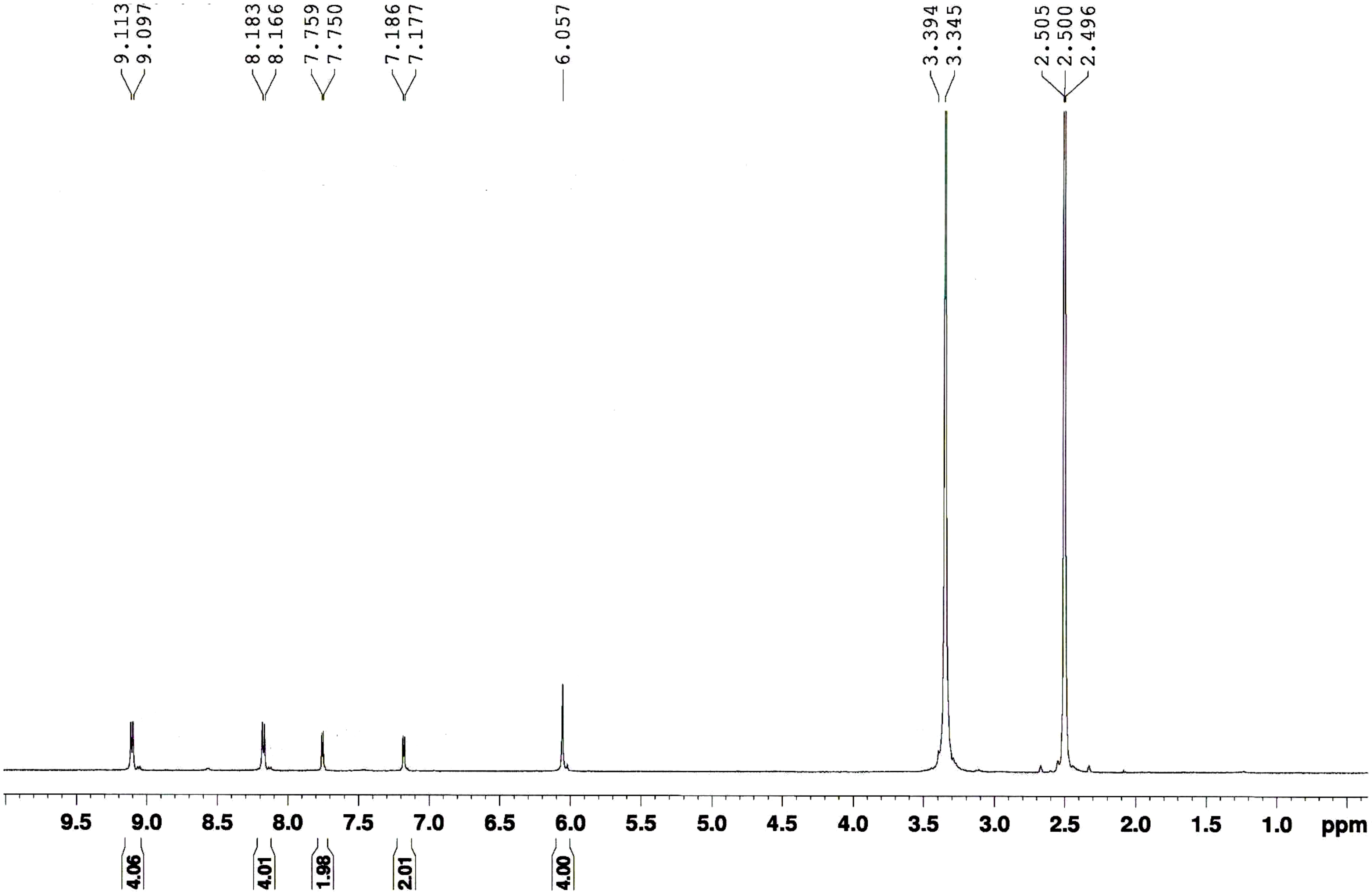

Figure S26. ^{13}C-NMR spectrum of compound **13**

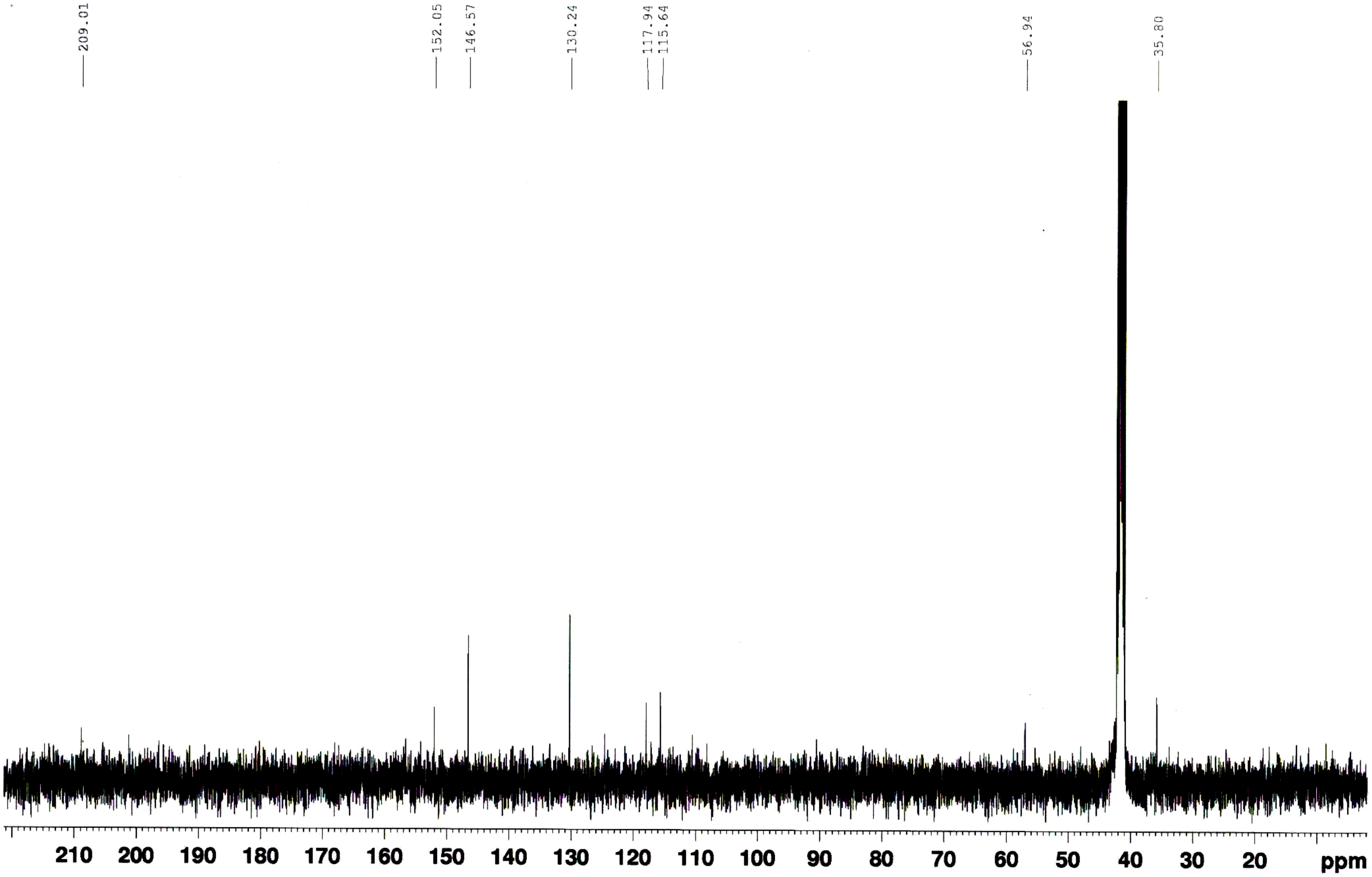

Figure S27. ^{1}H-NMR spectrum of compound **14**

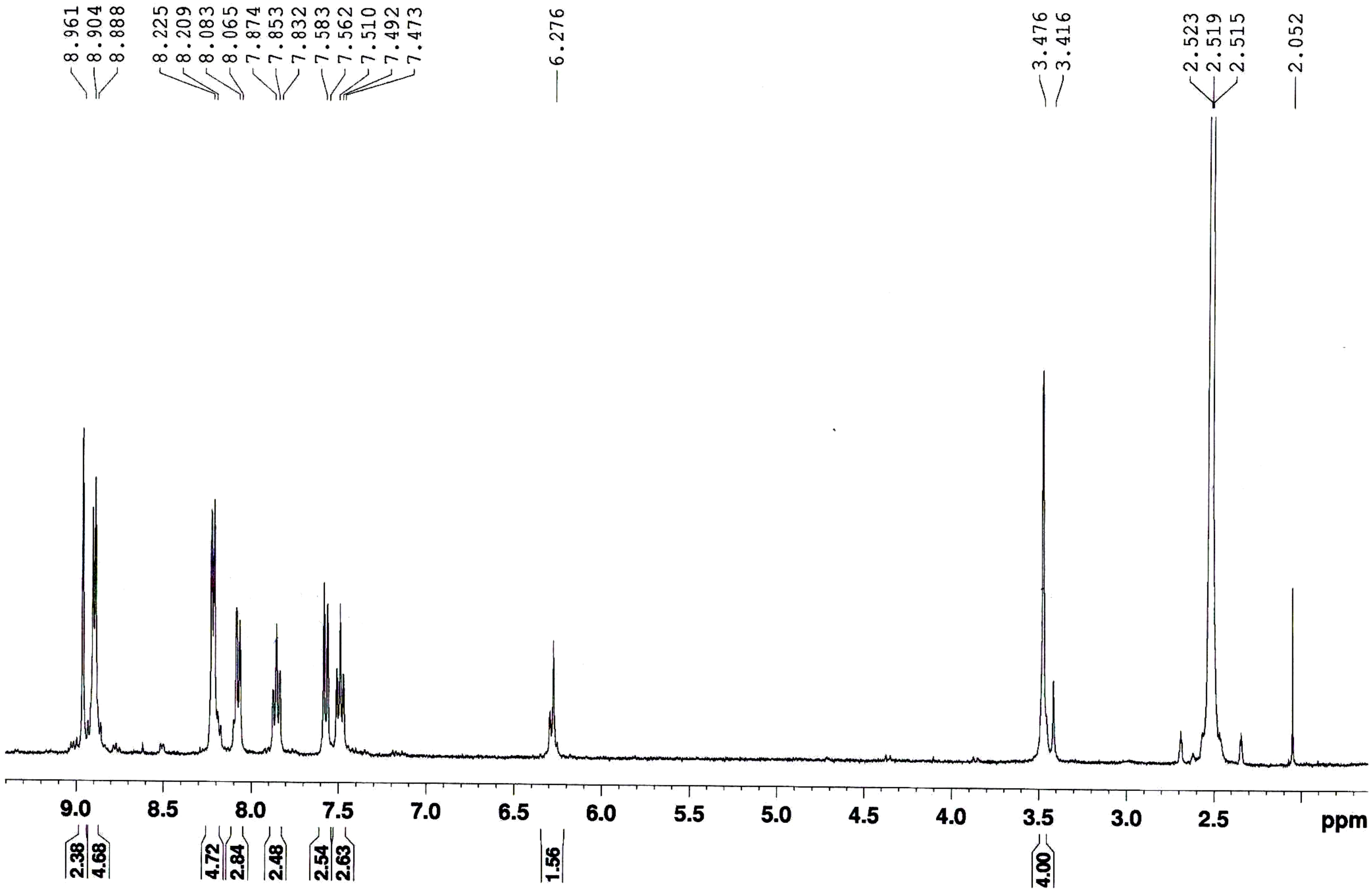

Figure S28. ^{13}C-NMR spectrum of compound **14**

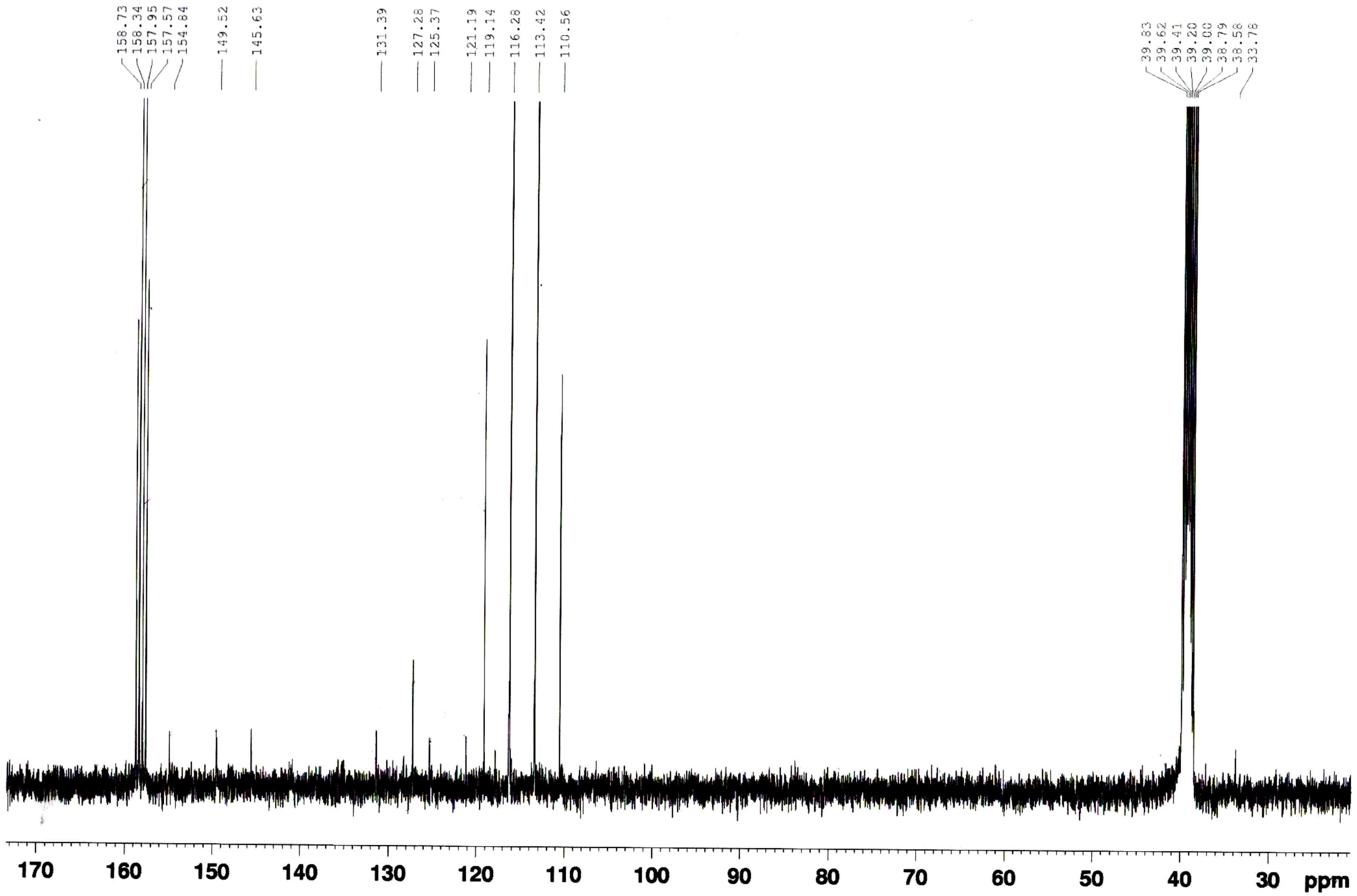

Figure S29. ^{1}H-NMR spectrum of compound **15**

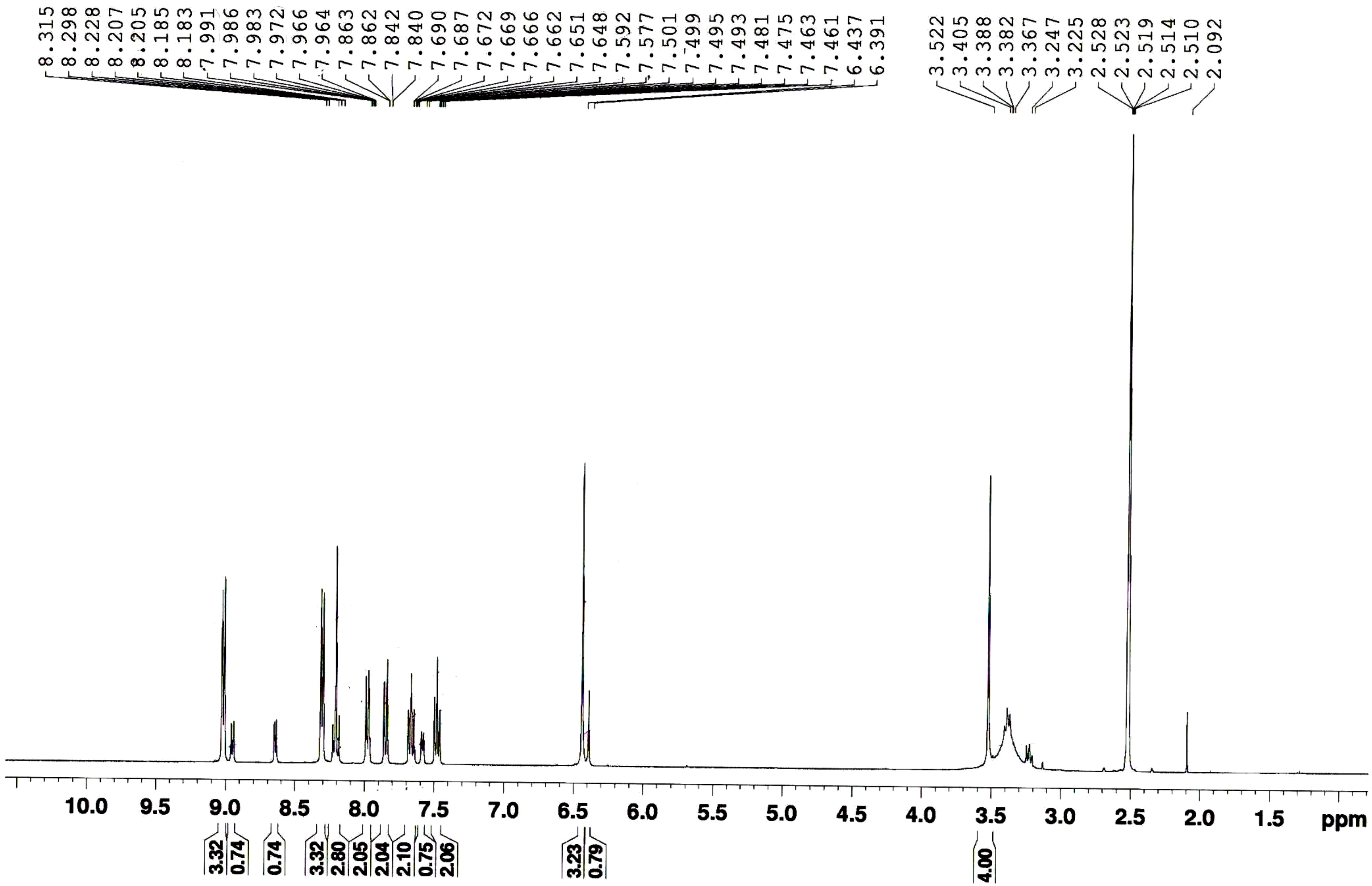

Figure S30. ^{13}C-NMR spectrum of compound **15**

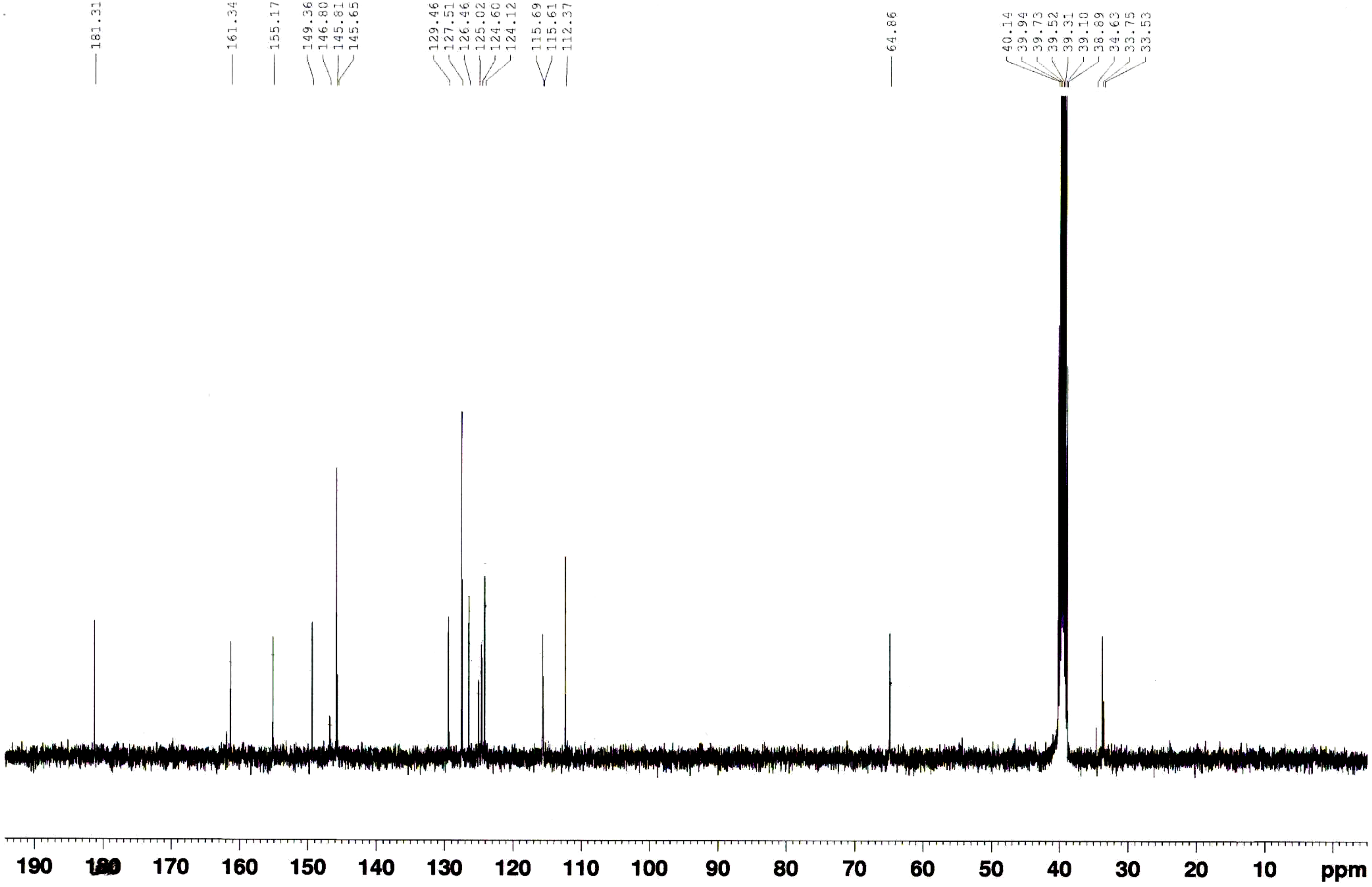

Figure S31. FTIR spectrum of compound **1**

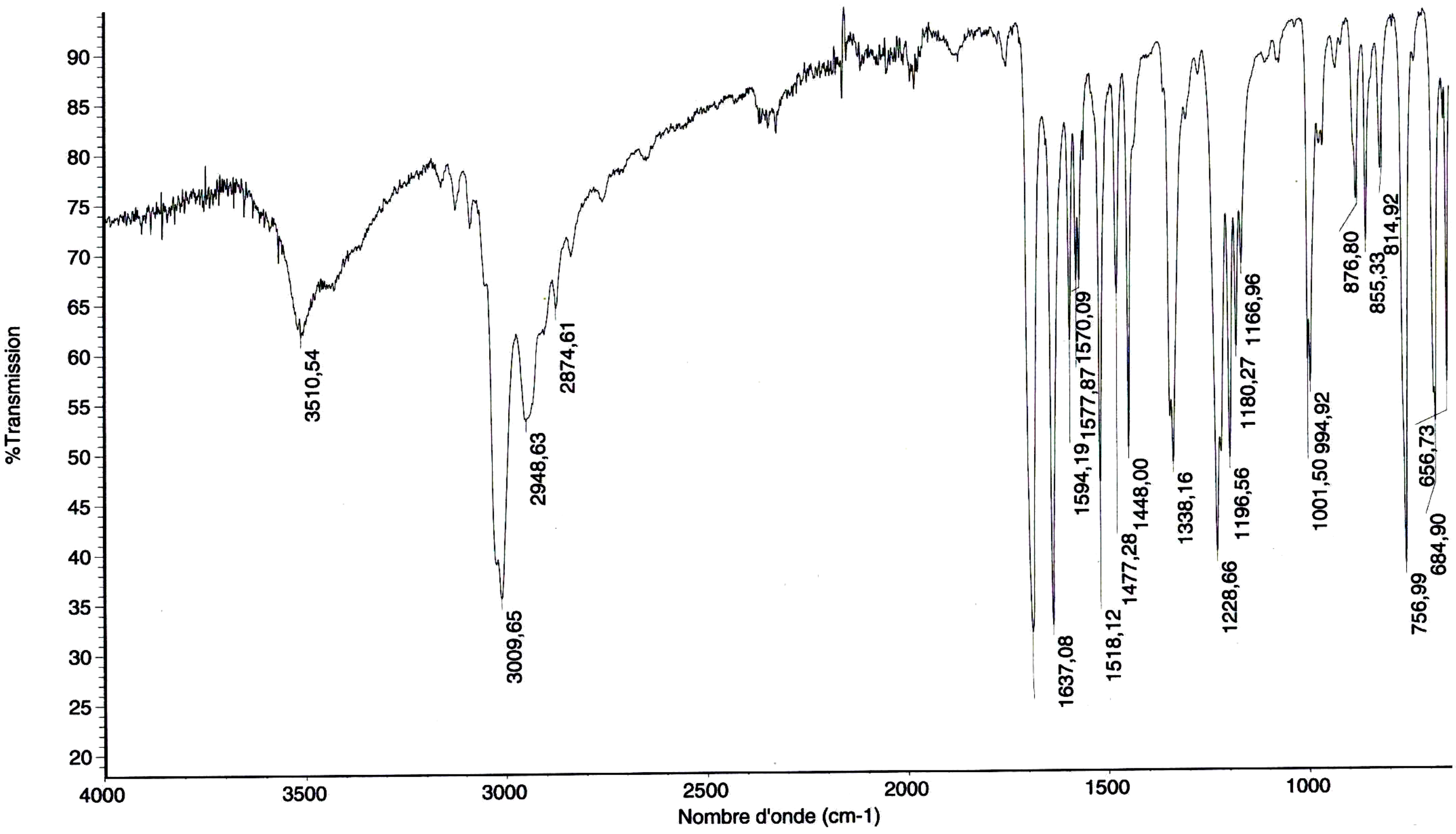

Figure S32. FTIR spectrum of compound **2**

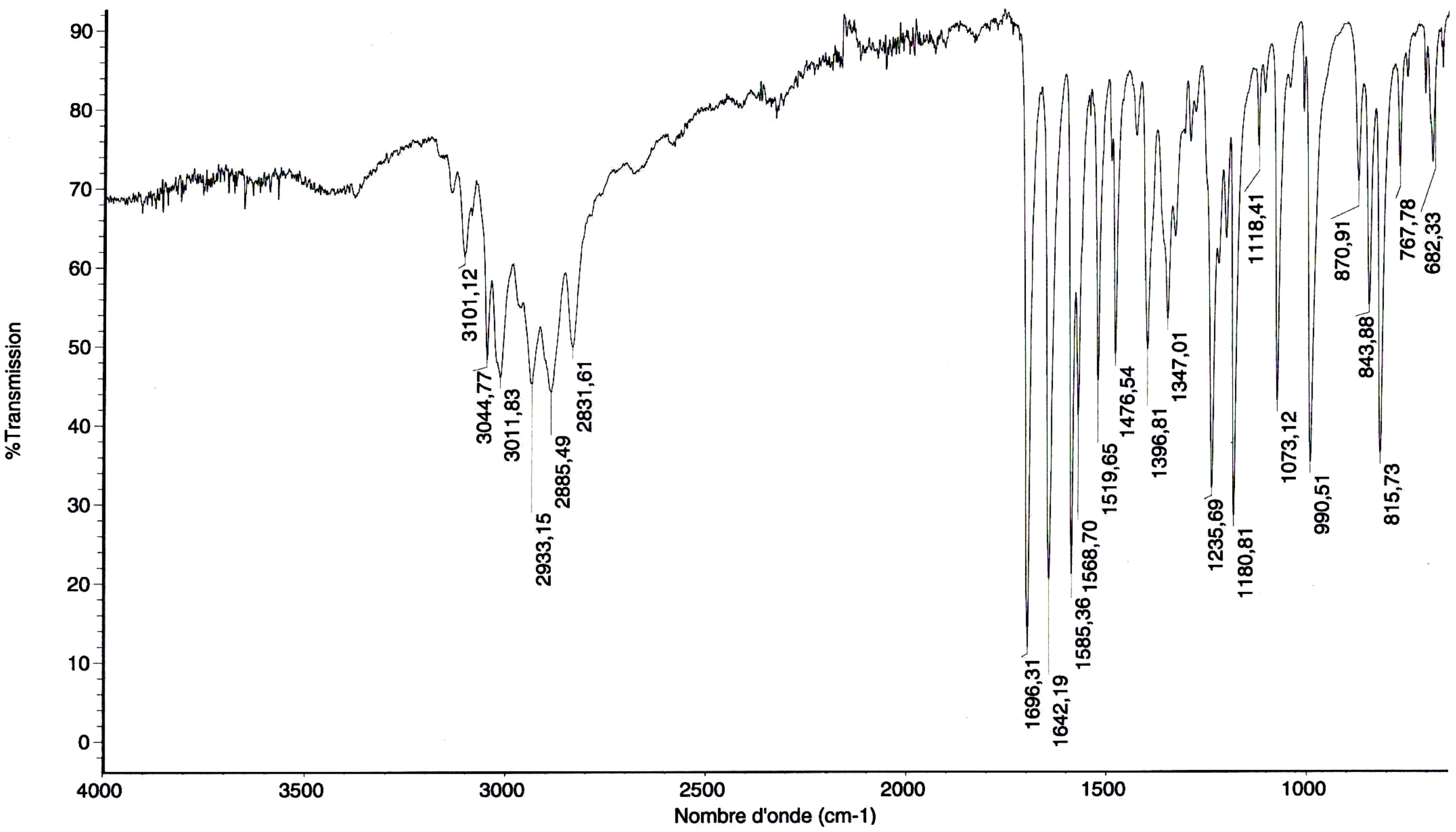

Figure S33. FTIR spectrum of compound **3**

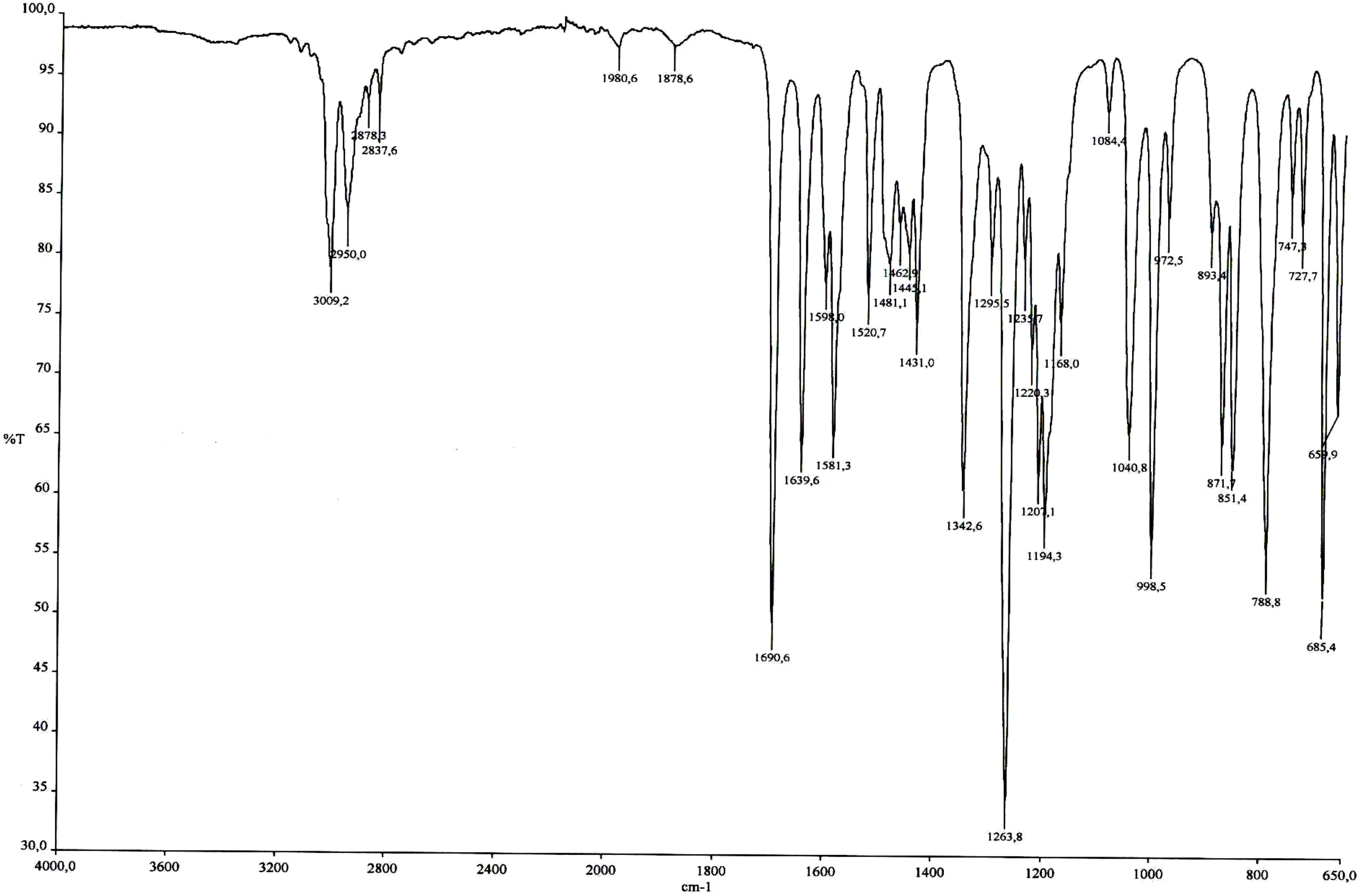

Figure S34. FTIR spectrum of compound **4**

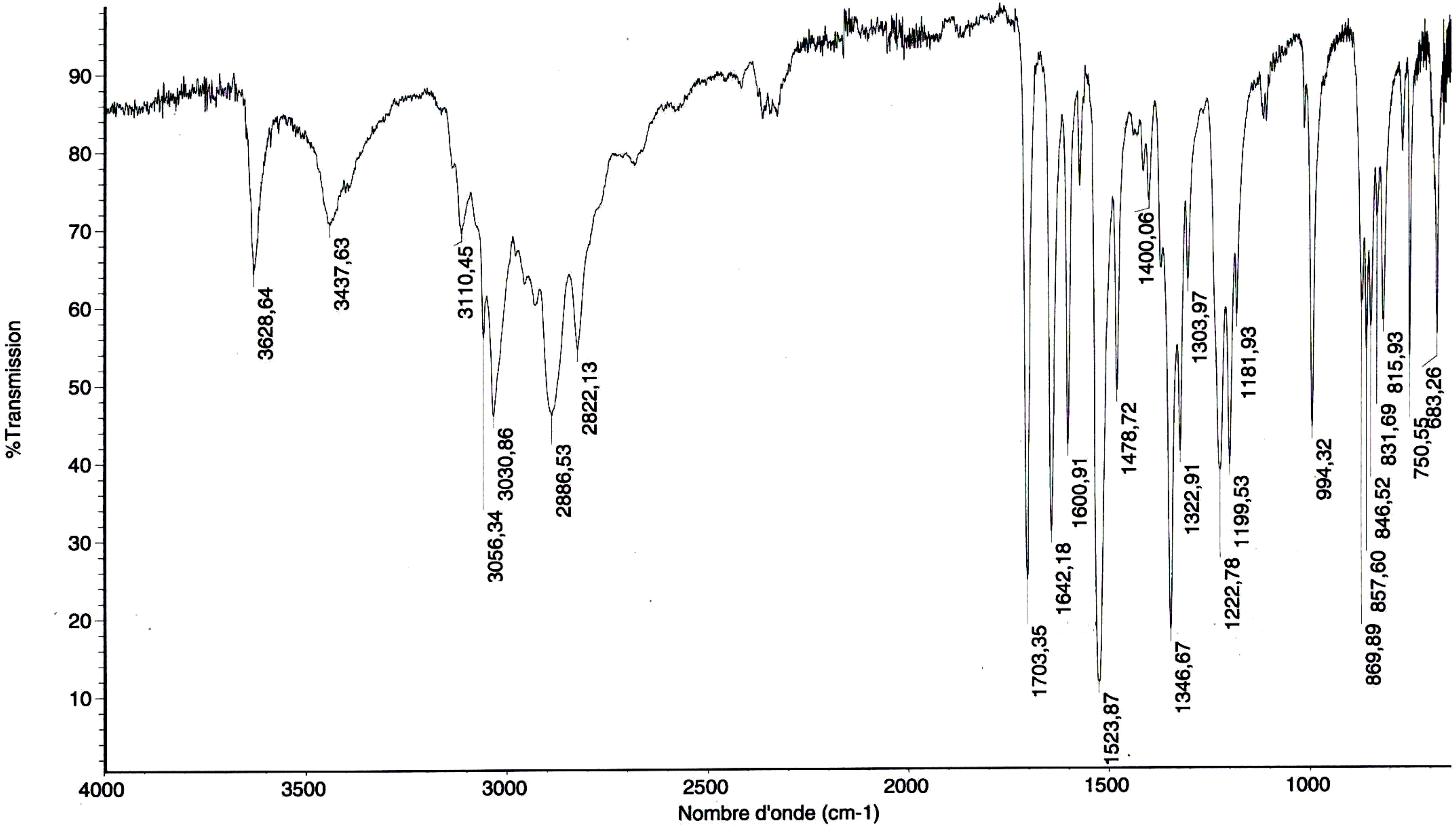

Figure S35. FTIR spectrum of compound **5**

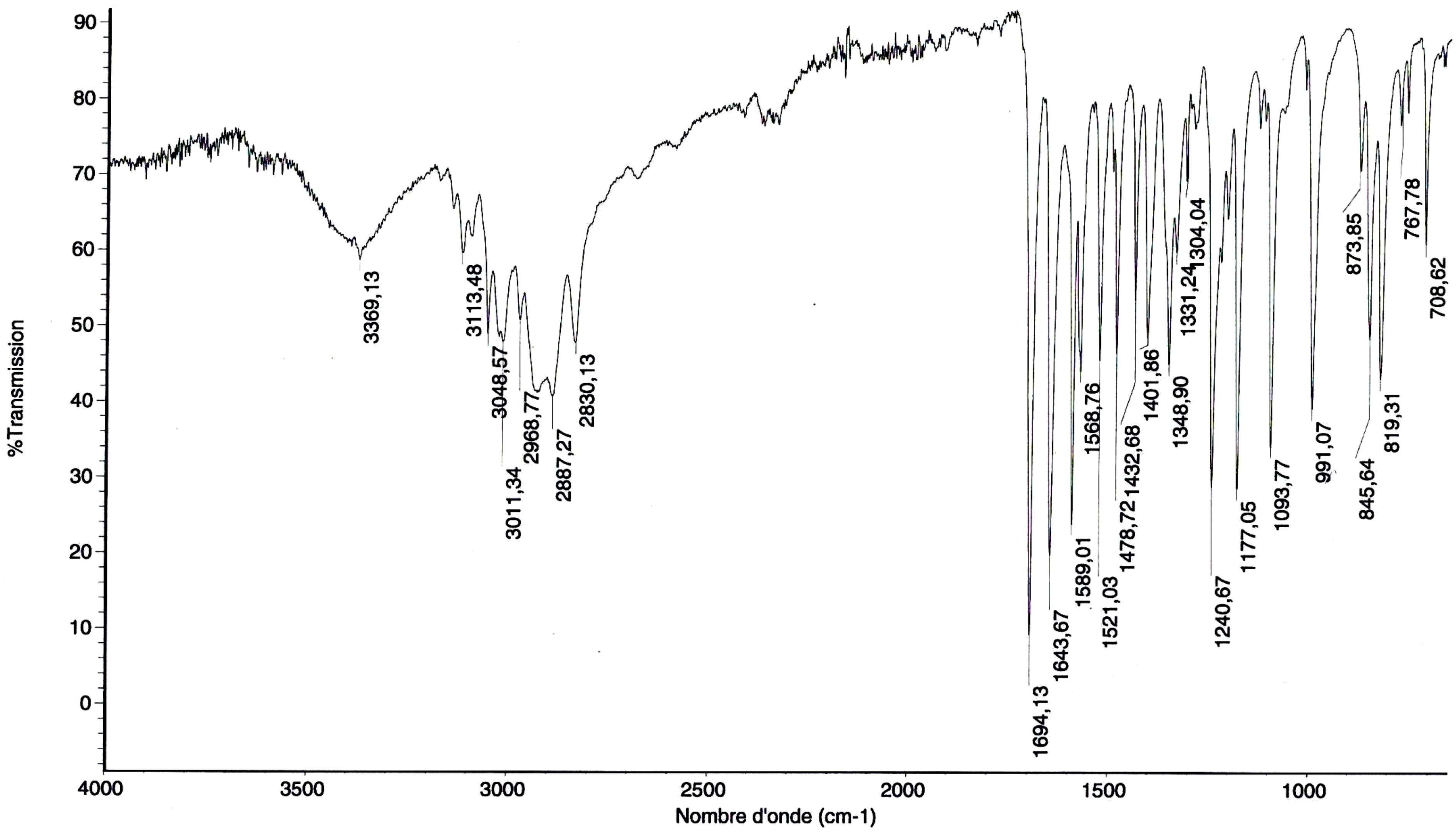

Figure S36. FTIR spectrum of compound **6**

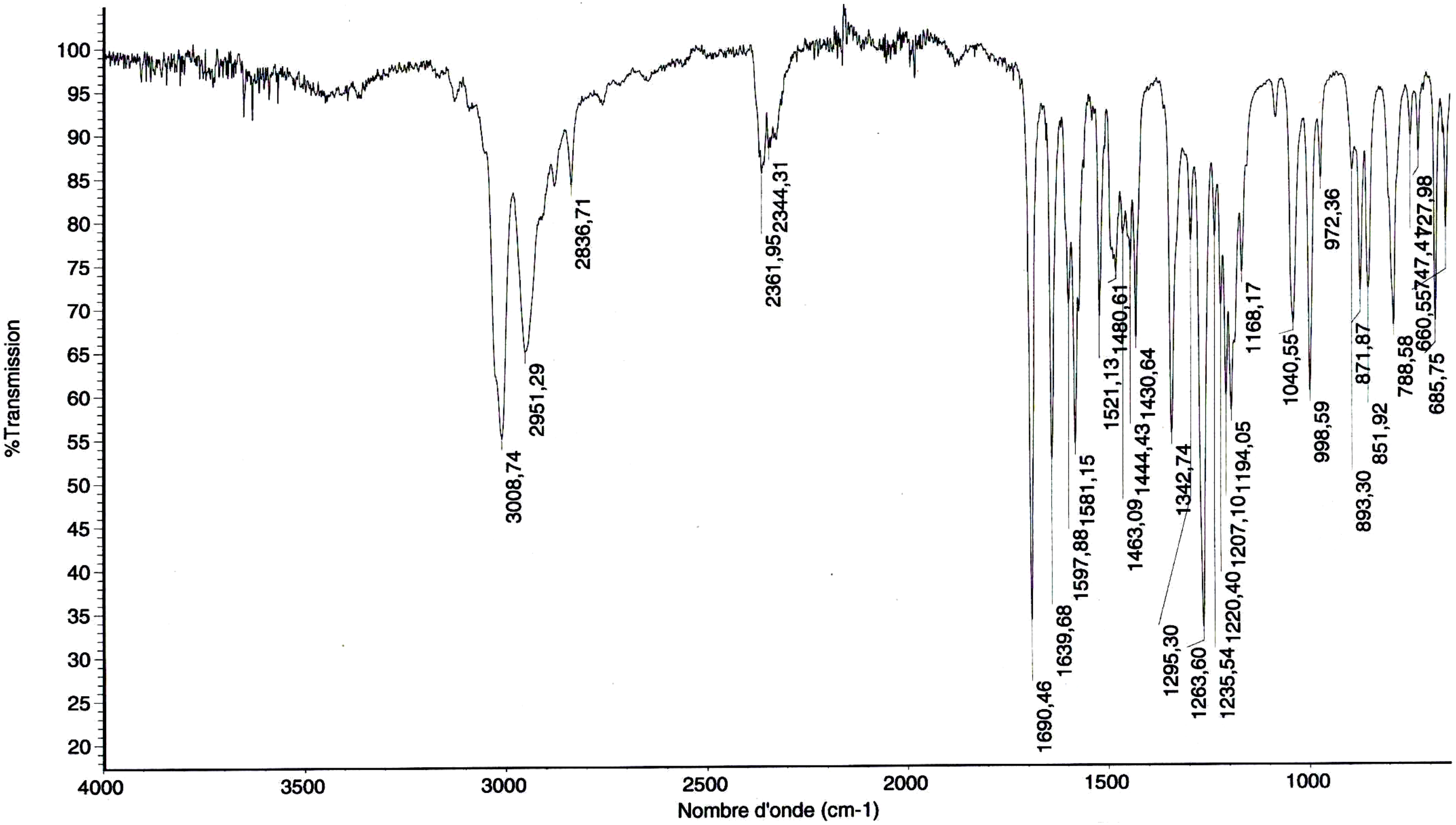

Figure S37. FTIR spectrum of compound **7**

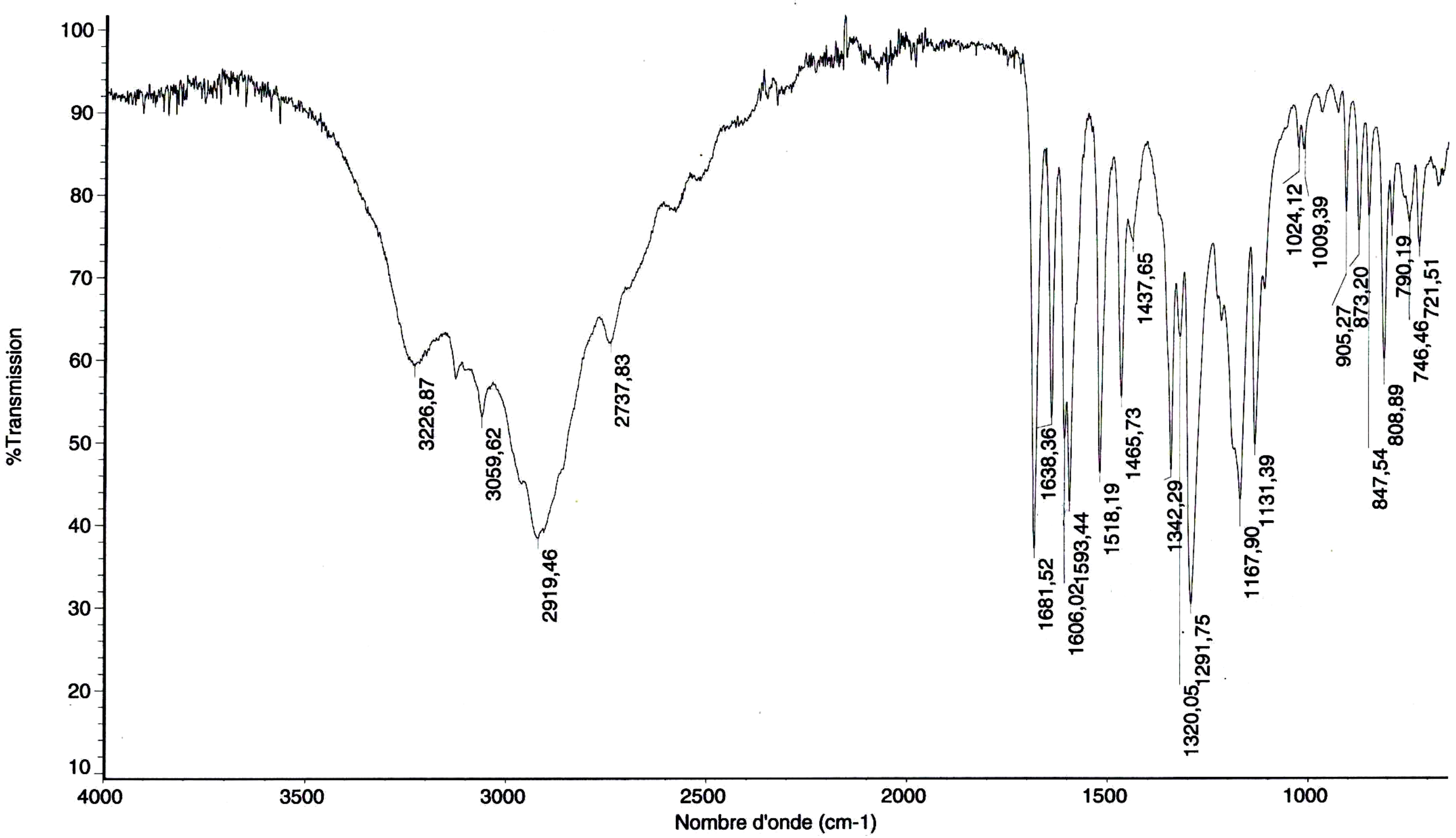

Figure S38. FTIR spectrum of compound **8**

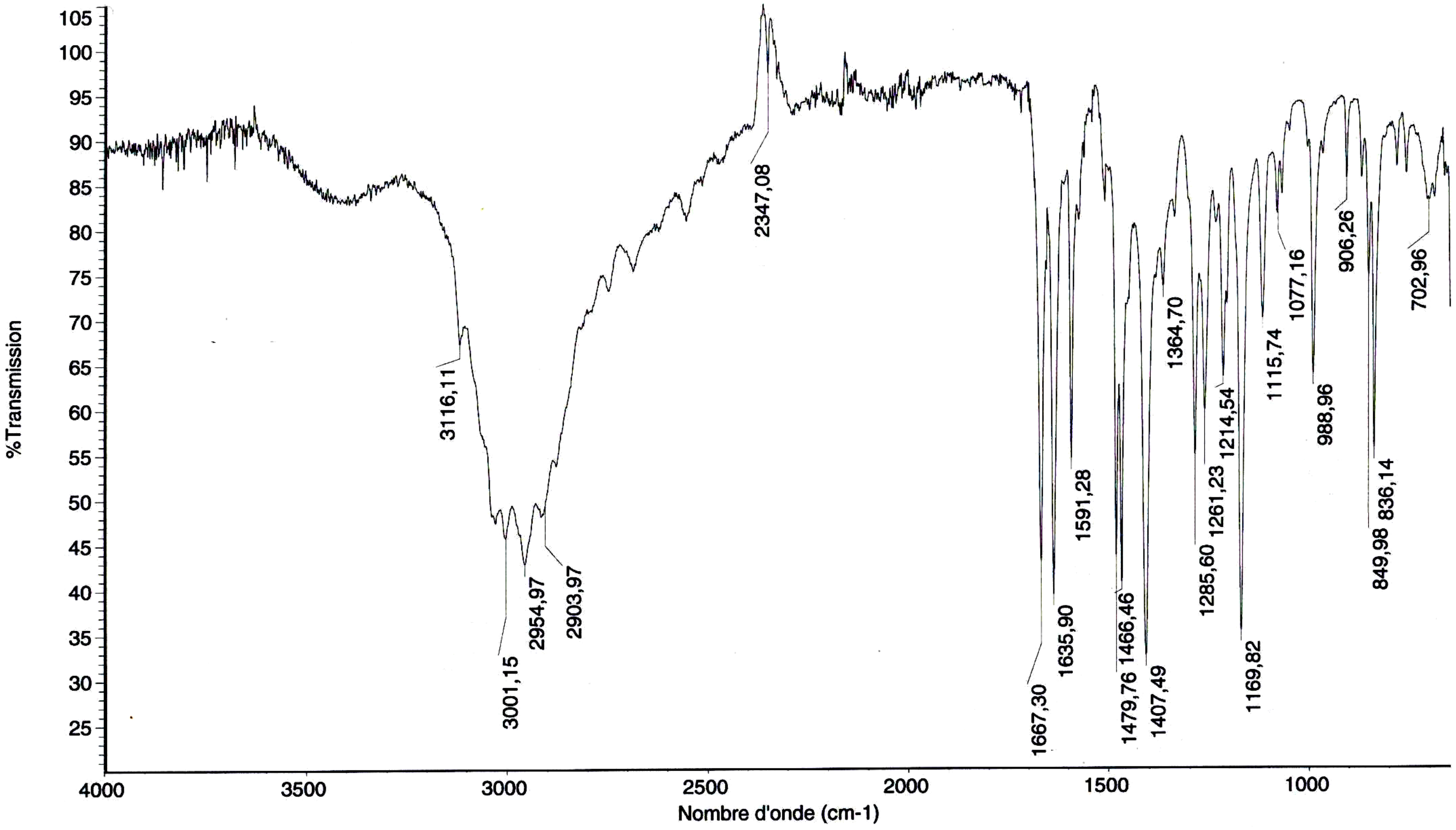

Figure S39. FTIR spectrum of compound **9**

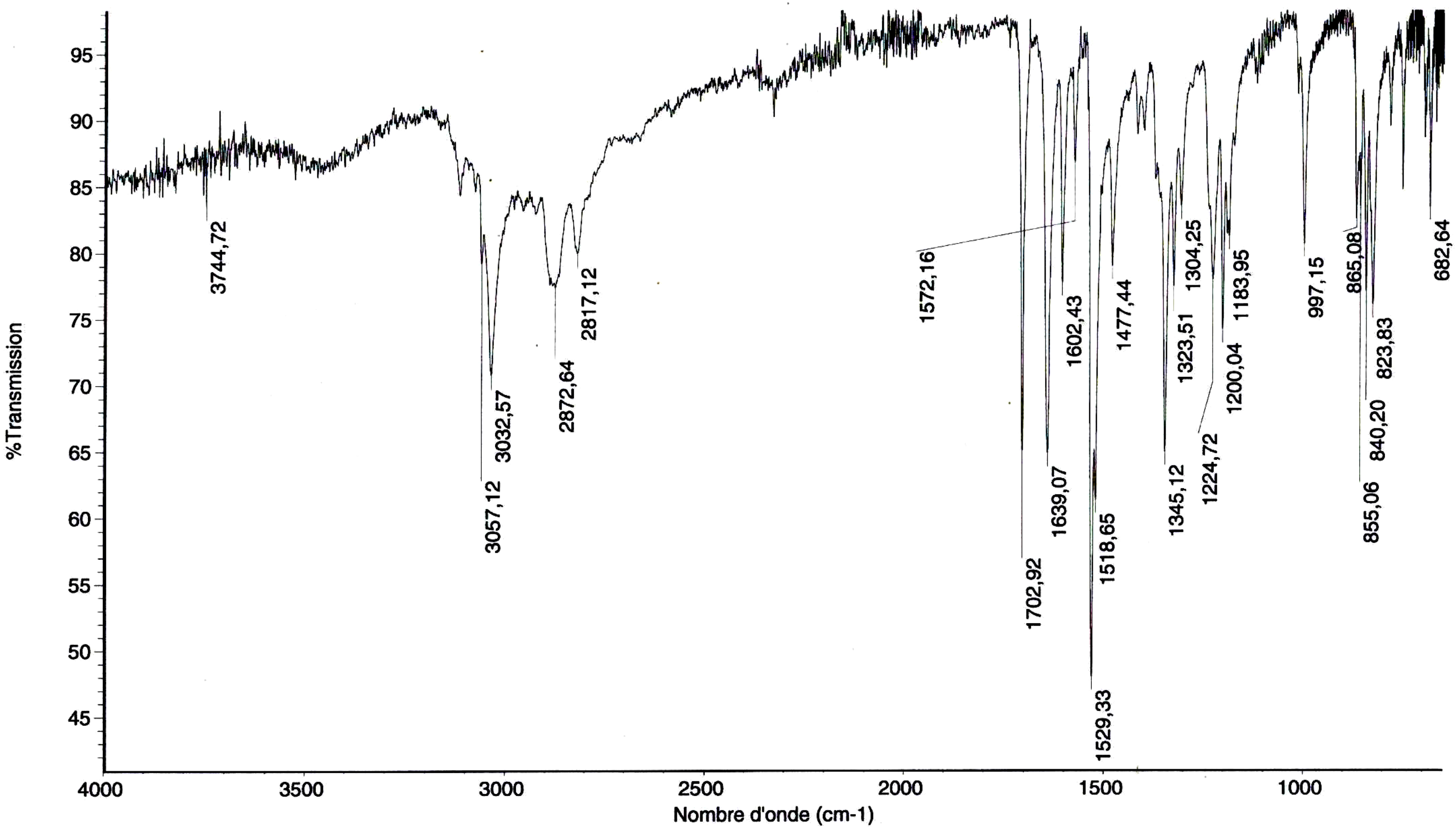

Figure S40. FTIR spectrum of compound **10**

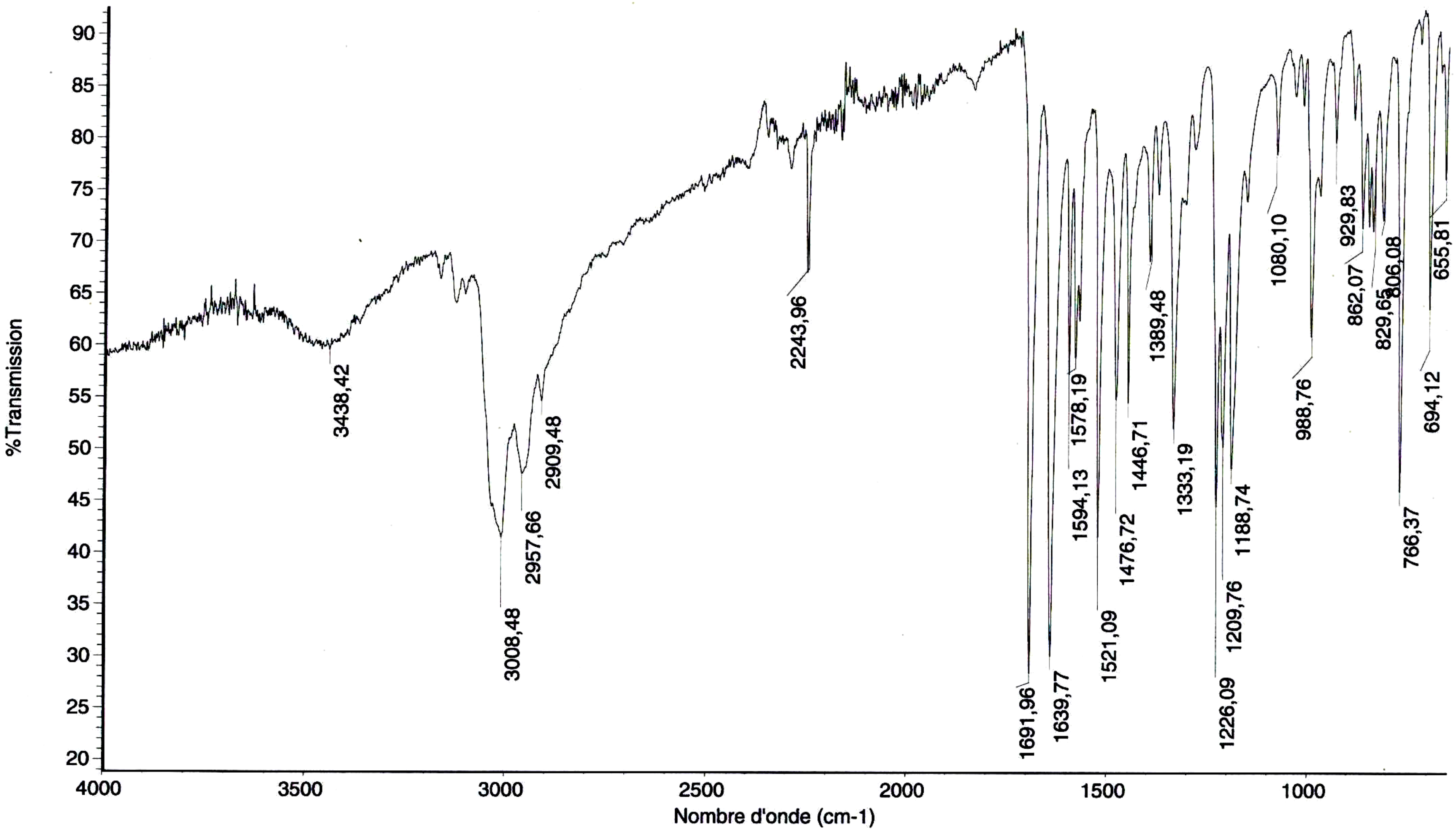

Figure S41. FTIR spectrum of compound **11**

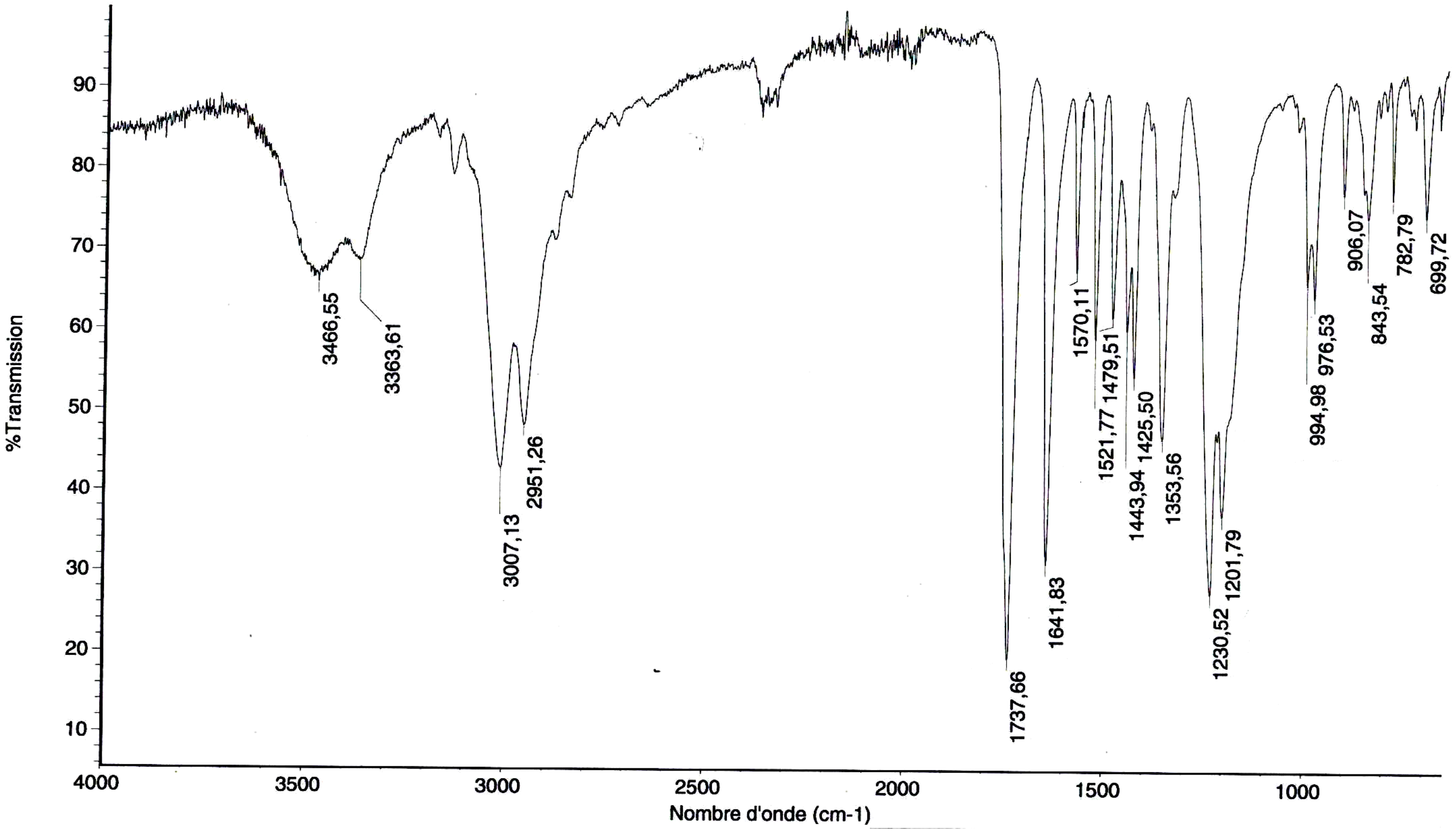

Figure S42. FTIR spectrum of compound **12**

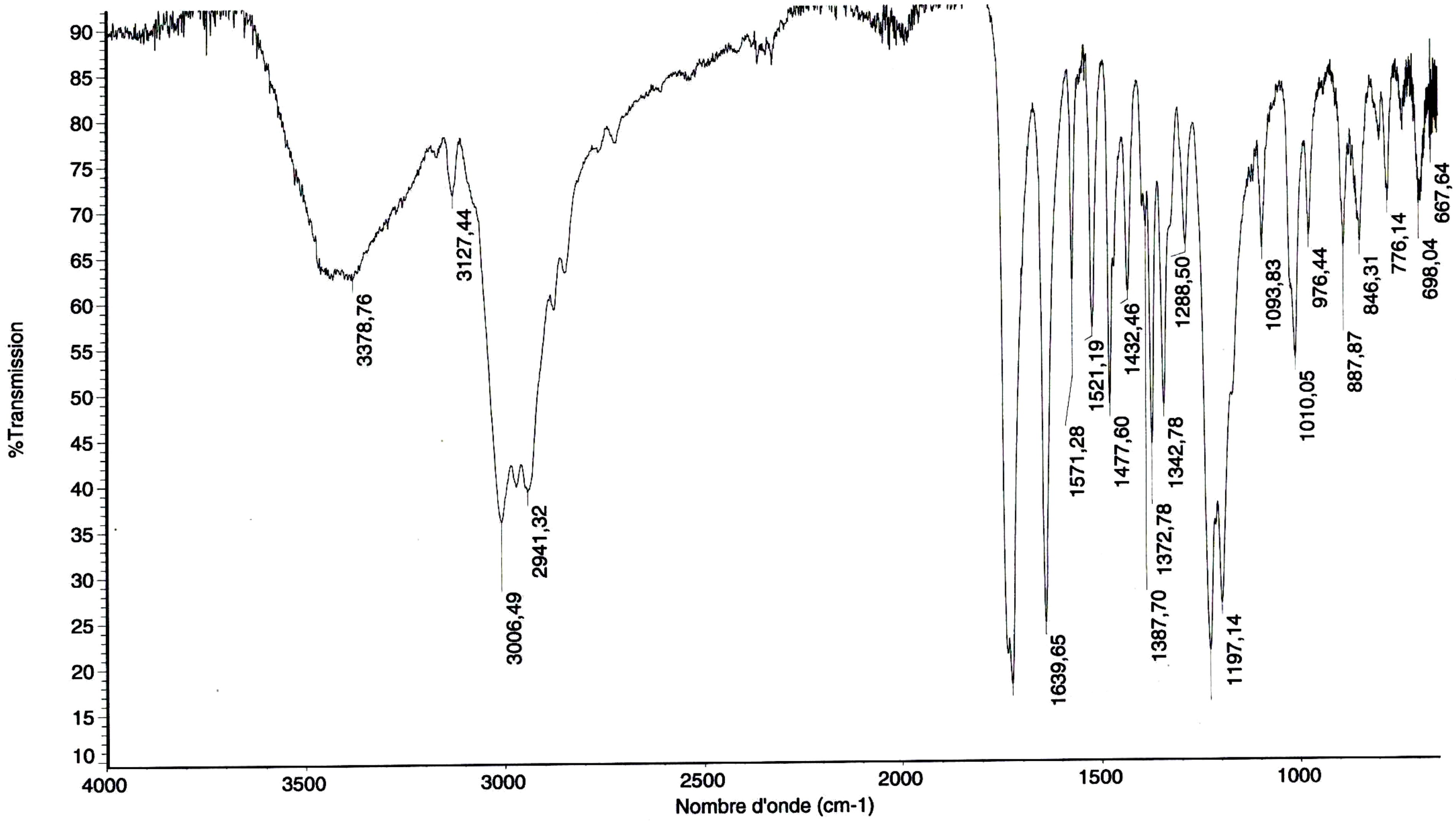

Figure S43. FTIR spectrum of compound **13**

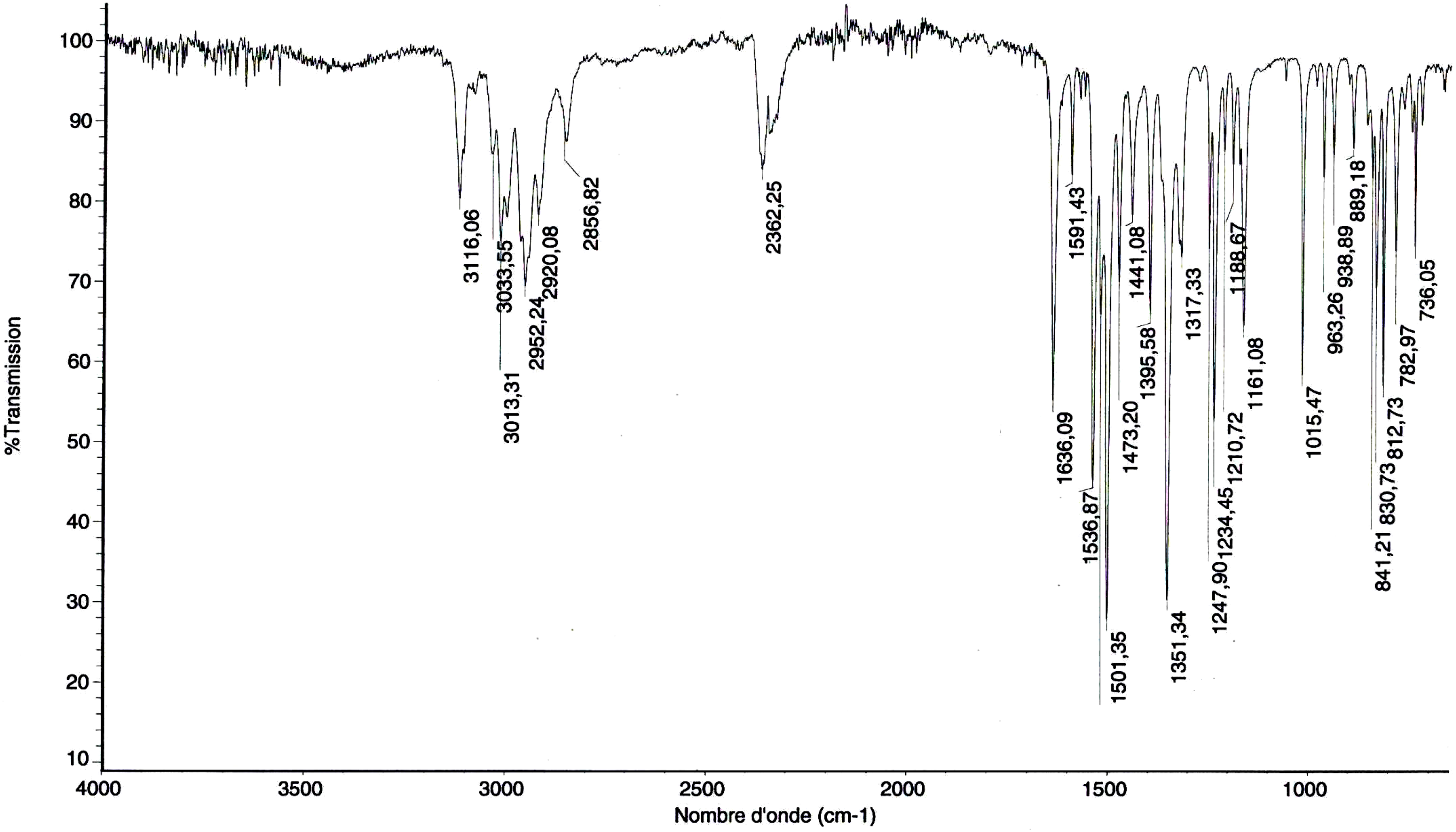

Figure S44. FTIR spectrum of compound **14**

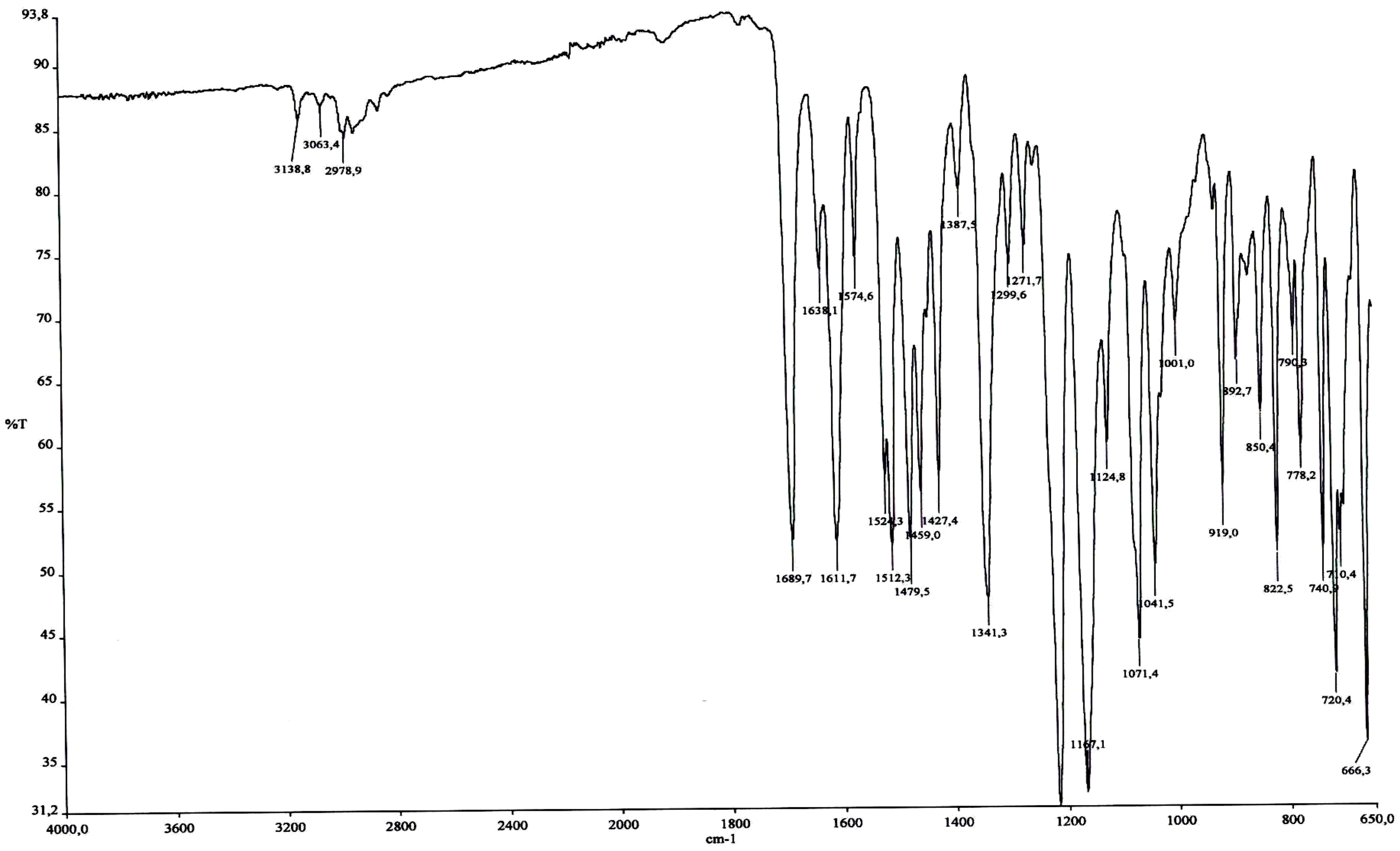

Figure S45. FTIR spectrum of compound **15**

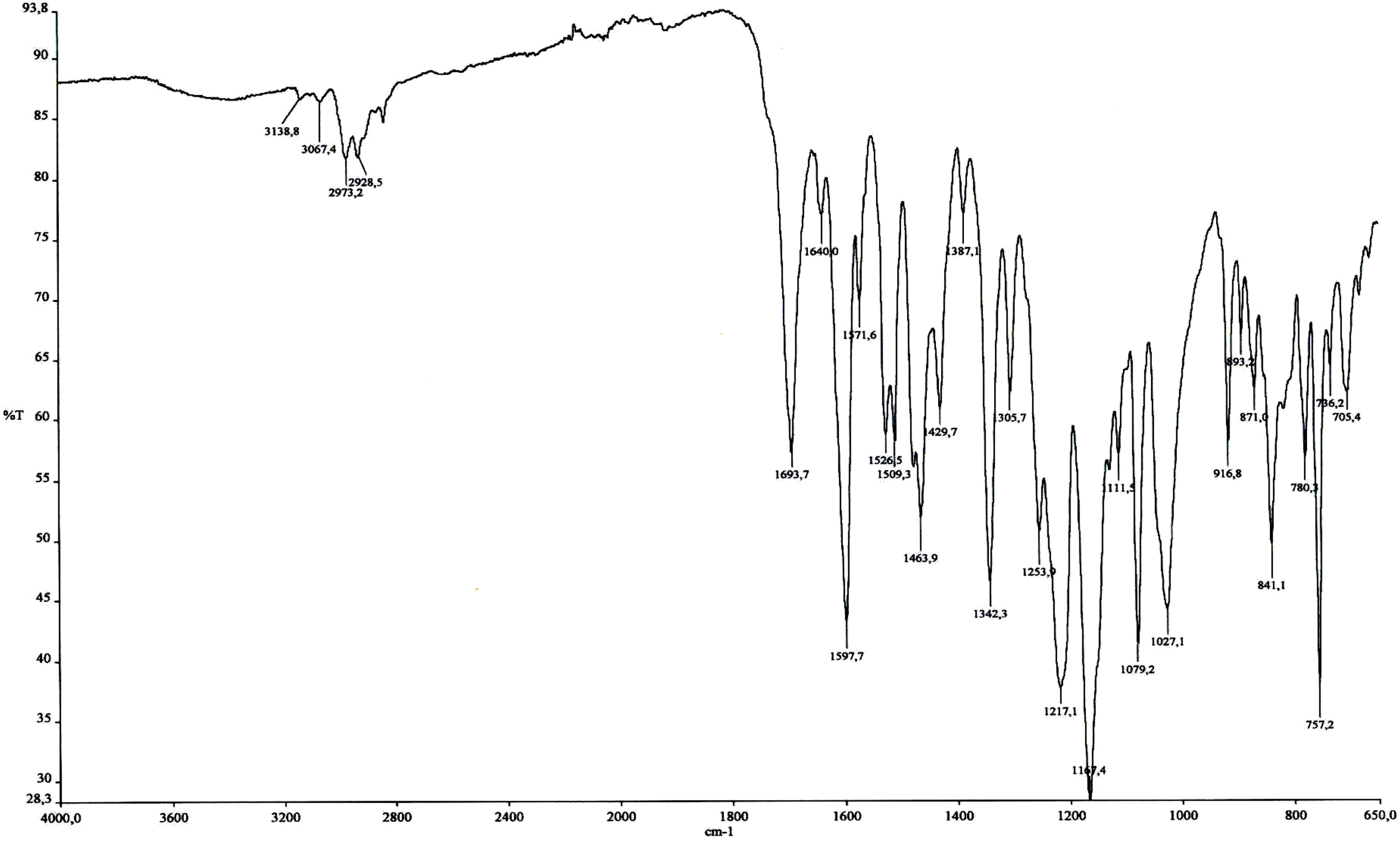

Supplement: Supplementary file 1 [file molecules-24-02424-s001.pdf]
